# Synthesis, Reactivity and Coordination Chemistry of Group 9 PBP Boryl Pincer Complexes: [(PBP)M(PMe_3_)*_n_*] (M = Co, Rh, Ir; *n* = 1, 2)

**DOI:** 10.3390/molecules28176191

**Published:** 2023-08-22

**Authors:** Philipp M. Rutz, Jörg Grunenberg, Christian Kleeberg

**Affiliations:** 1 Institute of Inorganic and Analytical Chemistry, Technische Universität Braunschweig, Hagenring 30, 38106 Braunschweig, Germany; 2 Institute of Organic Chemistry, Technische Universität Braunschweig, Hagenring 30, 38106 Braunschweig, Germany

**Keywords:** boron, diborane(4), boryl complex, PBP pincer ligand

## Abstract

The unsymmetrical diborane(4) derivative [(d(CH_2_P(*i*Pr)_2_)abB)–Bpin] (**1**) proved to be a versatile PBP boryl pincer ligand precursor for Co(I) (**2a**, **4a**), Rh(I) (**2**–**3b**) and Ir(I/III) (**2**–**3c**, **5**–**6c**) complexes, in particular of the types [(d(CH_2_P(*i*Pr)_2_)abB)M(PMe_3_)_2_] (**2a**–**c**) and [(d(CH_2_P(*i*Pr)_2_)abB)M–PMe_3_] (**2b**–**c**). Whilst similar complexes have been obtained before, for the first time, the coordination chemistry of a homologous series of PBP pincer complexes, in particular the interconversion of the five- and four-coordinate complexes **2a**–**c**/**3a**–**c**, was studied in detail. For Co, instead of the mono phosphine complex **2a**, the dinitrogen complex [(d(CH_2_P(*i*Pr)_2_)abB)Co(N_2_)(PMe_3_)] (**4a**) is formed spontaneously upon PMe_3_ abstraction from **2a** in the presence of N_2_. All complexes were comprehensively characterised spectroscopically in solution via multinuclear (VT-)NMR spectroscopy and structurally in the solid state through single-crystal X-ray diffraction. The unique properties of the PBP ligand with respect to its coordination chemical properties are addressed.

## 1. Introduction

**Scheme 1:**
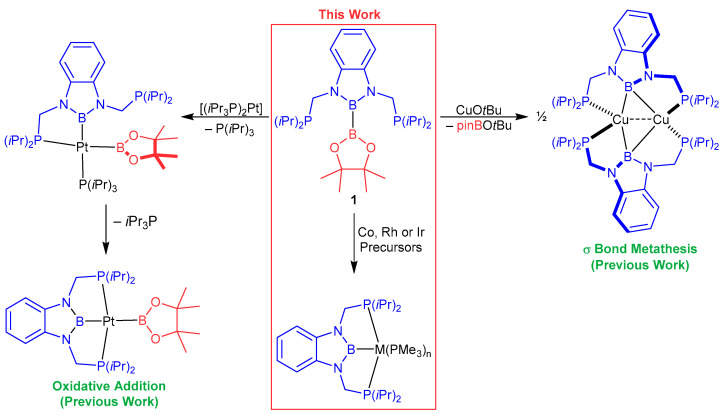
Formation of PBP pincer boryl complexes from a diborane(4) precursor [15].

Since their first report in 2009 by Nozaki, Yamashita and a co-worker, PBP pincer ligands with a diaminoboryl framework have been explored with respect to their coordination chemistry with various transition metals, in particular cobalt, rhodium and iridium, as well as with respect to potential applications in different catalytic and stoichiometric processes [1,2,3,4,5,6,7]. Whilst the majority of boryl pincer complexes are of this PBP diaminoboryl type, a number of boryl pincer complexes with other ligands frameworks, often quite unique ones, have also been reported [8,9,10,11,12,13,14]. Transition metal PBP diaminoboryl pincer complexes are fundamentally accessible through the oxidative addition of a hydridoborane ligand precursor, possibly followed by further modifications, a route already developed by Nozaki and Yamashita in their seminal work [1,2,3,4,5,6,7]. To overcome the inherent obstacles by this ‘B–H oxidative addition route’, we recently developed an unsymmetrical diborane(4), pinB–B(d(CH_2_P(*i*Pr)_2_)ab) (**1**) (pin = (OCMe_2_)_2_, d(CH_2_P(*i*Pr)_2_)ab = 1,2-(N(CH_2_P(*i*Pr)_2_))_2_C_6_H_4_), as a versatile PBP ligand precursor. This precursor provides direct access to PBP complexes through σ bond metathesis, as exemplified with the copper boryl complex [(d(CH_2_P(*i*Pr)_2_)abB)Cu]_2_, and, alternatively, oxidative addition, as exemplified with the platinum *bis*-boryl complexes *cis*-[(d(CH_2_P(*i*Pr)_2_)abB)(*i*Pr_3_P)Pt–Bpin] and trans-[(d(CH_2_P(*i*Pr)_2_)abB)Pt–Bpin] (Scheme 1) [15].

In the present work, we endeavoured to explore the use of pinB–B(d(CH_2_P(*i*Pr)_2_)ab) (**1**) as a precursor for a series of group 9 PBP boryl pincer complexes and study their fundamental coordination chemistry. To facilitate the access to a range of PBP boryl pincer complexes, we chose three easily available group 9 metal complexes [(Me_3_P)_4_Co–Me], [(Me_3_P)_3_Rh–Cl] and [(cod)Ir–Cl]_2_ as precursors [16,17,18].

## 2. Results

### 2.1. Cobalt Complexes

**Scheme 2:**
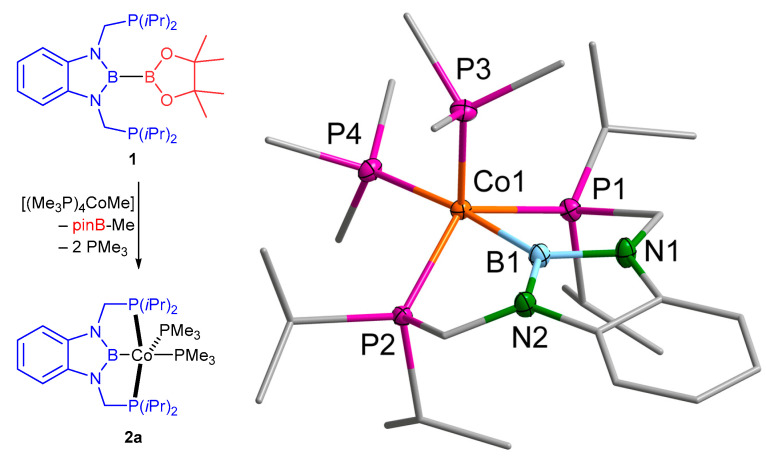
Formation of PBP cobalt boryl complexes **2a** (**left**) and its molecular structure (**right**). Selected distances [Å] and angles [°]: Co1–B1 1.936(2), Co1–P1 2.2063(4), Co1–P2 2.1859(3), Co1–P3 2.1643(4), Co1–P4 2.1968(4), P1–Co1–P2 125.55(2), P2–Co1–P3 112.03(1), P1–Co1–P3 110.56(2), B1–Co1–P4 173.85(5), B1–Co1–P3 83.24(5), Co1–[P1,P2,P3] 0.4372(3).

The reaction of **1** with [(Me_3_P)_4_Co–Me] results in the mono boryl complex [(d(CH_2_P(*i*Pr)_2_)abB)Co–(PMe_3_)_2_] (**2a**) (Scheme 2), presumably via an oxidative addition/reductive elimination pathway [19,20,21]. The reaction delivers **2a** after 24 h at 50 °C as dark orange crystals in a 66% isolated yield. A single crystal X-ray diffraction study on **2a** revealed a five coordinate 18-valence electron Co(I) complex (Scheme 2). The complex **2a** crystallises in an achiral non-centrosymmetric space group of the type *Pca*2_1_ with four molecules in the unit cell (Z = 4, Z’ = 1) (Supplementary Materials) [22].

The coordination environment at the cobalt atom in **2a** is best described as distorted trigonal bipyramidal with the boryl ligand and one PMe_3_ ligand in the apical positions, and the angle between these positions deviates by 7° from linearity. Moreover, the strong σ donor properties of the boryl ligand result in an elongation of the Co1–P4 distance of the apical PMe_3_ ligand, compared to distance Co1–P3 of the equatorial PMe_3_ ligand by 0.03 Å.

The equatorial positions are occupied by the two pincer phosphine donors and a second PMe_3_ ligand, resulting in a sum of angles in the equatorial plane [P1,P2,P3] of 348.14°, whereby the angle P1–Co1–P2, involving the two pincer phosphorus atoms, is slightly larger than the other angles. For the deviation of the sum of angles, from 360° accounts for the significant displacement of Co1 from the [P1,P2,P3] plane by 0.4372(3) Å towards the P4 atom. This distortion of the trigonal bipyramidal coordination environment at the cobalt atom is due to the restraints imposed by the five-ring chelates in **2a**. Whilst the solid-state molecular structure of **2a** does not exhibit any crystallographic symmetry, it is virtually *C_s_* symmetric, with a mirror plane through the atoms [B1,P3,P4,Co1] (Figure S37). 

An analogous unrestraint mono boryl complex [(PMe_3_)_4_Co–Bcat] (cat = 1,2-O_2_C_6_H_4_) exhibits a slightly longer B–Co distance of 1.9545(4) Å and a slightly shorter trans-B P–Co distance of 2.1897(1) Å, together with a less pronounced displacement of the cobalt atom from the equatorial ligand plane [21]. The equatorial Co–P distance in [(PMe_3_)_4_Co–Bcat], however, is more equally distributed around 2.17 Å. The closely related square planar PBP complex [(d(CH_2_P(*t*Bu)_2_)abB)Co–N_2_], reported to undergo reversible H_2_ activation by Peters and a co-worker, exhibits similar Co–B and Co–P distances of 1.946(1) Å and 2.1884(4)/2.1901(3) Å and also significant deviation of the P–Co–P angle from linearity of 156.26(1)° as a result of the five-ring chelation [3].

**Figure 1:**
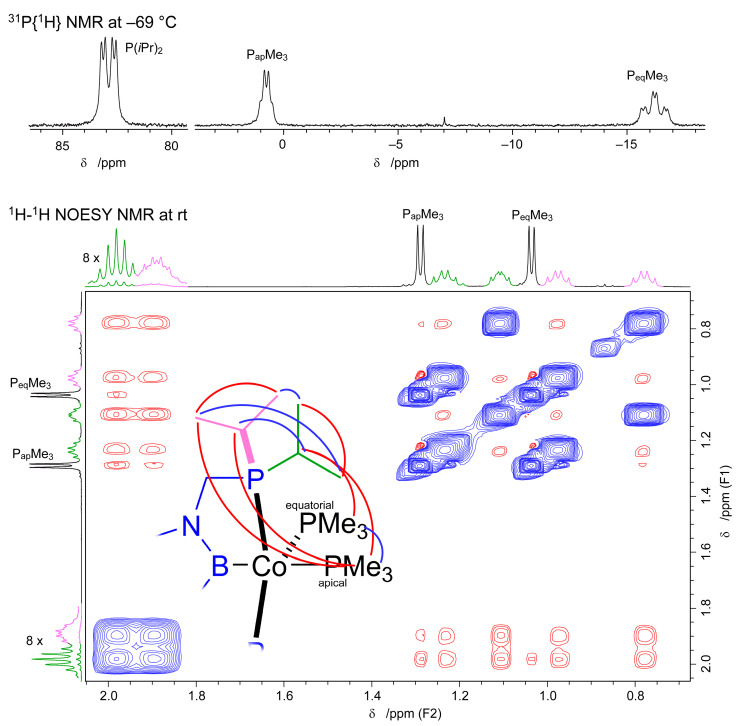
^31^P{^1^H} NMR spectrum of **2a** at –69 °C (**top**), and a section of the ^1^H-^1^H NOESY NMR spectrum of **2a** (**bottom**), selected exchange (blue) and NOE (red) correlations are depicted (PhMe-d_8_, 400.4/162.1 MHz, rt).

The ^31^P{^1^H} NMR spectrum of **2a** comprises three distinct signals, two signals of the two distinct PMe_3_ ligands, one in the apical—trans boryl—position around 0 ppm and the second around –16 ppm for the equatorial PMe_3_ ligand. The third signal around 83 ppm is assigned to the two equivalent PBP pincer ligand P(*i*Pr)_2_ groups (Figure 1(top) and Figure S4). Whilst these signals do not exhibit any fine structure at room temperature (Figure S4), at lower temperatures, the signals split in a doublet of doublets at 82.9 ppm (–69 °C) for the P(*i*Pr)_2_ groups, an apparent broadened quartet at 0.7 ppm (–69 °C) for the apical PMe_3_ ligand and a triplet of doublets at –16.2 ppm (–69 °C) for the equatorial PMe_3_ ligand (Figure 1(top) and Figure S4). This is in agreement with the mutual couplings within an A_2_MN spin system. This agrees with a conformation of the complex in solution similar to the one found in the solid state. 

However, the temperature-dependent broadening is indicative of dynamic processes present in solution. A ^1^H-^1^H NOESY NMR spectrum at room temperature gives a fitting picture. Distinct NOE contacts between the PMe_3_ signals and the methine C*H*Me_2_ signals allow for the assignment of the PMe_3_ ligands to the apical and equatorial positions, respectively. Exchange signals are observed between the two PMe_3_ ligands, but also between pairs of methyl groups of the two distinct isopropyl moieties and the methine protons of these groups (Figure 1 (bottom)). This is fundamentally in agreement with two possible exchange mechanisms: (i) via the dissociation of a PMe_3_ ligand with a transient four coordinate 16-electron complex [(d(CH_2_P(*i*Pr)_2_)abB)Co–PMe_3_] and the re-association of a PMe_3_ ligand; (ii) a concerted mechanism exchanging the PMe_3_ ligand positions via a (distorted) square pyramidal intermediate is feasible. Note also that the NMR data do not suggest any appreciable dissociation of PMe_3_ from **2a**, contrary to the heavier rhodium homolog **2b** (vide infra). 

**Scheme 3:**
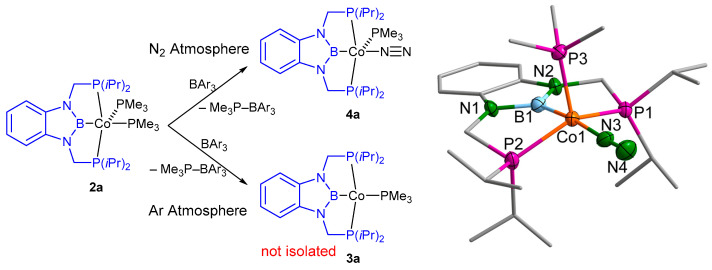
Reaction of **2a** with BAr_3_ (Ar = Ph, C_6_F_5_) and molecular structures of [(d(CH_2_P(*i*Pr)_2_)abB)Rh–(N_2_)(PMe_3_)] (**4a**). Selected distances [Å] and angles [°]: Co1–B1 1.942(6), Co1–P1 2.1949(15), Co1–P2 2.2175(16), Co1–P3 2.1798(16), Co1–N3 1.816(5), N3–N4 1.118(7), P1–Co1–P2 134.51(7), P2–Co1–P3 110.78(6), P1–Co1–P3 104.85(6), B1–Co1–P3 89.5(2), B1–Co1–N3 172.2(2), Co1–[P1,P2,P3] 0.3915(9).

The reaction of **2a** with an equimolar amount of BAr_3_ as a Lewis acid should lead to abstraction of a PMe_3_ ligand and, after reorganisation, to the complex [(d(CH_2_P(*i*Pr)_2_)abB)Co–PMe_3_] (**3a**). However, whilst one PMe_3_ ligand can indeed be abstracted by BPh_3_, the complex **3a** is not isolated. Instead, in a dinitrogen atmosphere, its dinitrogen adduct [(d(CH_2_P(*i*Pr)_2_)abB)Co–(N_2_)(PMe_3_)] (**4a**) crystallises in minute amounts after several days at –40° C (Scheme 3).

The N_2_ complex **4a** crystallises in a space group of the type *P*2_1_/*c* with two independent molecules in the asymmetric unit (Z = 8, Z’ = 2) (Supplementary Materials) [22]. Both molecules exhibit only a marginal geometric difference, and only one is discussed exemplarily (Figure S40). 

As for **2a**, the coordination geometry of **4a** is best described as distorted trigonal bipyramidal with the boryl ligand and the N_2_ ligand in the apical positions. The B–Co distance remains virtually unchanged by this substitution of the trans boryl ligand and is also identical to the distance found in the closely related four-coordinate PBP complex [(d(CH_2_P(*t*Bu)_2_)abB)Co–N_2_] reported by Peters et al. of 1.946(1) Å [3]. This indicates again that the B–Co distance is largely determined by the geometrical restraints of the five-ring chelates (vide infra). The close to linear B–Co–N, the angle deviates only by less than 7° from the value found in **2a** and in Peter’s N_2_ complex [3]. The equatorial ligands experience more substantial changes, although their sum of angles around the cobalt atom increases only slightly by 2° to 350.14°, and consistently, the deviation of the cobalt atom from the [P1,P2,P3] plane decreases by 0.05 Å. The angle between the pincer P atoms deviates significantly by 9°; hence, **4a** is more distorted from an ideal trigonal bipyramidal geometry towards a square pyramidal arrangement than **2a**. However, the reduced steric demand of the ligand in the apical position trans to the boryl ligand in **4a** as compared to **2a** leads to a relaxation of the B1–Co1–P3 angle by about 7°. The N–N distance in the N_2_ ligand in **4a** compares well with the distance of 1.119(2) Å found in Peter’s N_2_ complex; the N–Co distance, however, is in **4a** slightly—by 0.035 Å—enlarged [3].

**Figure 2:**
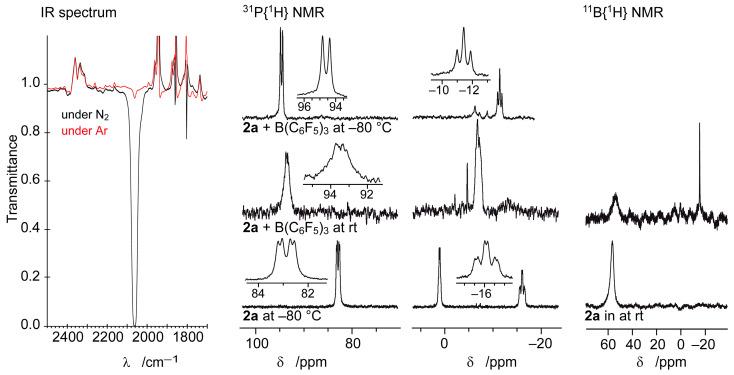
IR (in PhMe), ^31^P{^1^H} NMR and ^11^B{^1^H} spectra of **2a** + B(C_6_F_5_)_3_ at rt and –80 °C and of isolated **2a** (PhMe-d_8_, 162.1/96.3 MHz, rt).

As we failed to isolate **4a** in any appreciable amounts, we resorted to its spectroscopic in situ characterisation (Figure 2). Performing the reaction of **2a** with B(C_6_F_5_)_3_ in toluene and monitoring this reaction via IR spectroscopy gives clear evidence of the immediate formation of an N_2_ complex, based on the appearance of a strong IR band at 2061 cm^−1^ if the reaction is conducted under an N_2_ atmosphere, whereas only a minute signal is observed under an argon atmosphere, presumably due to the presence of adventitious N_2_ (Scheme 3). This compares well to the N≡N stretching frequency of 2013 cm^−1^ reported for the related complex [(d(CH_2_P(*t*Bu)_2_)abB)Co–N_2_] (vide supra) by Peters et al. [3]. 

Following the reaction of **2a** with B(C_6_F_5_)_3_ under an N_2_ atmosphere via ^31^P and ^11^11B NMR spectroscopy (Figure 2) gives a consistent picture: upon addition of the Lewis acid, the chemical shifts change from those of **2a** (Figure 2). Whilst the ^11^B{^1^H} NMR signal shifts only by about 3 ppm, it gives evidence for the presence of the PBP boryl ligand. The changes in the ^31^P{^1^H} NMR spectrum are more substantial. The two ^31^P NMR signals of the PMe_3_ ligands in **2a** change to a broad signal at −13 ppm and a second comparably narrow signal at −7 ppm without an appreciable fine structure. Upon cooling, however, the latter signal broadens, and its intensity reduces, whist the former signal changes into a well-developed triplet (−11.4 ppm, ^2^*J*_PP_ = 73 Hz) at −80 °C (Figure 2). The latter triplet corresponds to the doublet at higher chemical shifts (94.6 ppm, ^2^*J*_PP_ = 73 Hz). This is readily explained by the abstraction of one PMe_3_ ligand to give the Lewis acid base adduct Me_3_P–B(C_6_F_5_)_3_ (*δ*_31P_ = −6.1 ppm, *δ*_11B_ = −14.7 ppm in CD_2_Cl_2_) and a PBP pincer cobalt complex bearing only one additional PMe_3_ ligand Me_3_P–B(C_6_F_5_)_3_ is only sparingly soluble and to a large extend removed prior to the measurement. The remaining dissolved adduct, however, precipitates upon cooling resulting in a reduced ^31^P NMR signal at lower temperatures. The chemical shift of −11.4 ppm and the P–P coupling constant of around 80 Hz suggest that this PMe_3_ ligand occupies an equatorial position in a trigonal bipyramidal complex, as it resembles the chemical shift, but in particular, the higher P_eq_–P_PBP_ coupling constant found in **2a**. In other words, the complex that is quantitatively formed is not the four-coordinate complex **3a** but a five coordinate complex with a single PMe_3_ ligand in an equatorial position—the nitrogen complex **4a**. 

Gas-phase DFT computations on the thermodynamics of the complexes **3a** and **4a** and their heavier homologues (vide infra) as central atoms indeed show that for cobalt as the central atom, the formation of a five-coordinate N_2_ complex of the type [(d(CH_2_P(*i*Pr)_2_)abB)M–(N_2_)(PMe_3_)] is strongly favoured over the four coordinate complex [(d(CH_2_P(*i*Pr)_2_)abB)M–(PMe_3_)] by Δ*G*_298_ = −22.4 kJ mol^−1^ (Δ*E*_0_ = −70.6 kJ mol^−1^), despite the entropic penalty occurring from the coordination of gaseous N_2_. However, for the rhodium and iridium analogue, the coordination of an N_2_ ligand to the latter four-coordinate complex is—in agreement with our observations (vide infra)—disfavoured by Δ*G*_298_ = 51.3 kJ mol^−1^ (Δ*E*_0_ = 8.2 kJmol^−1^) for rhodium and Δ*G*_298_ = 51.4 kJ mol^−1^ (Δ*E*_0_ = 8.4 kJ mol^−1^) for iridium (Supplementary Materials) [22]. The computed N≡N stretching frequency in **4a** of 2170 cm^−1^ is by about 100 cm^−1^ off the experimental values, but within the expected range considering the harmonic nature of the computation and other approximations [22]. 

Due to an initial computation of the force constant between Co and N_2_, the bonding in **4a** is quite strong (Co–N: 2.33 N cm^−1^), whilst the trans-B Co–P bond in **2a** shows the expected kinetic lability (Co–P: 1.36 N cm^−1^) of a spectator ligand. More importantly, the electronic coupling in **4a** between the N–N bond and the Co–N coordination is pronounced (Co–N/N–N coupling force constant: −0.02 cm N^−1^) and synergistic (negative sign), pointing to an effective back donation [23]. And indeed, the experimental N_2_ IR wavenumber of 2061 cm^−1^ is in line with a modest activation relative to free N_2_ (~2330 cm^−1^). Finally, the Co–B bond trans to the N_2_ ligand seems to be very strong (Co–B: 2.28 N cm^−1^), reducing the flexibility to access different coordination geometries [24].

### 2.2. Rhodium Complexes

**Scheme 4:**
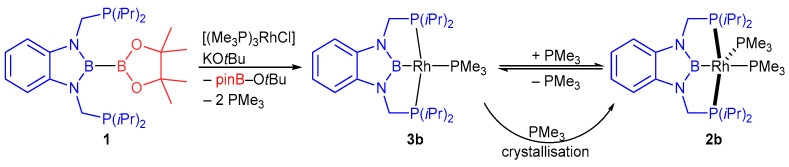
Formation of PBP rhodium boryl complexes **3b** and **2b**.

The reaction of **1** with [(Me_3_P)_3_Rh–Cl] in the presence of KO*t*Bu leads to the formation of a rhodium(I) boryl complex (Scheme 4). It may be speculated that the reaction proceeds via an intermediate rhodium alkoxido complex as discussed for the formation of the related complex [(dmabB)Rh(PMe_3_)_3_] (dmab = 1,2-(NMe)_2_C_6_H_4_) [20]. However, the reaction of **1** with [(Me_3_P)_3_Rh–Cl] in the presence of KO*t*Bu leads to the formation of an equilibrium mixture of the square planar complex [(d(CH_2_P(*i*Pr)_2_)abB)Rh–PMe_3_] (**3b**) and the five coordinate complex [(d(CH_2_P(*i*Pr)_2_)abB)Rh(PMe_3_)_2_] (**2b**) (Scheme 4), whilst in the absence of KO*t*Bu, no reaction is observed (Supplementary Materials) [22] (Figures S12 and S13). After recrystallisation from diethyl ether, the four coordinate complex **3b** is obtained as bright orange crystals at a 70% yield, whereas crystallisation from *n*-pentane in the presence of an excess PMe_3_ leads to the isolation of the five-coordinate complex **2b** as crystalline material at a 43% yield. The spontaneous dissociation of one PMe_3_ ligand from **2b** to give **3b** is not contradicting gas-phase DFT computational data (Table S10), suggesting an endothermic (15 kJ mol^−1^) dissociation from **2b** to **3b** + PMe_3_, but overall, an entropy driven exergonic process (−47 kJ mol^−1^) (Supplementary Materials) [22].

**Figure 3:**
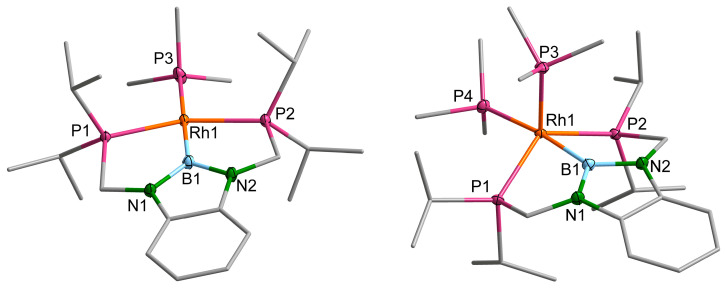
Molecular structures of the complexes [(d(CH_2_P(*i*Pr)_2_)abB)Rh–PMe_3_] (**3b**) (**left**) and [(d(CH_2_P(*i*Pr)_2_)abB)Rh(PMe_3_)_2_] (**2b**) (**right**). Selected distances [Å] and angles [°], **3b**: Rh1–B1 2.0221(5), Rh1–P1 2.2658(1), Rh1–P2 2.2794(1), Rh1–P3 2.3555(1), P1–Rh1–P2 152.622(5), B1–Rh1–P3 177.02(2), Rh1–[P1,P2,P3,B1] 0.0264(3); **2b**: Rh1–B1 2.0256(7), Rh1–P1 2.3262(2), Rh1–P2 2.3364(2), Rh1–P3 2.3167(2), Rh1–P4 2.3705(5), P1–Rh1–P2 127.894(6), P2–Rh1–P3 108.475(7), P1–Rh1–P3 110.722(7), B1–Rh1–P4 172.54(2), Rh1–[P1,P2,P3] 0.4833(3).

Both complexes **2b** and **3b** crystallise in monoclinic space groups of the type *P*2_1_/*n* and *P*2_1_/*c*, respectively, and contain one complex molecule in the asymmetric unit (Z = 4, Z’ = 1) (Supplementary Materials) [22]. The molecular structure of complex **2b** is analogous to that of the cobalt homologue **2a** (Figure S38). The rhodium ion is distorted trigonal bipyramidaly coordinated by the boryl pincer ligand and one PMe_3_ ligand in the apical positions (Figure 3, right). The sum of angles in the equatorial plane [P1,P2,P3] comprising the pincer phosphorus atoms and one PMe_3_ ligand is with 347° only insignificantly smaller than in **2a**, whereby the angle P1–Co1–P2, involving the pincer phosphorus atoms, is by about 2° larger than in **2a**. The displacement of Rh1 from the [P1,P2,P3] plane is by 0.05 Å larger than in **2a**, an effect of the increased radius of the rhodium ion within the restraining pincer coordination environment. 

Complex **3b** is best described as a distorted square planar complex with a nearly linear B1–Rh1–P3 angle and a significantly (by 27°), from linearity, deviating P1–Rh1–P2 angle. However, this angle is significantly closer to linearity than the respective angle in the five-coordinate complex **2b** (Figure 3, left). The change in the Rh···P/B distances between **2a** and **3b** is comparably small, despite the change in the coordination number. Most pronounced is a decrease in the pincer phosphorus atoms to rhodium distances in comparison to **2b** by about 0.06 Å, which may be attributed to the less strained ligand conformation in the more planar **3b**. 

**Figure 4:**
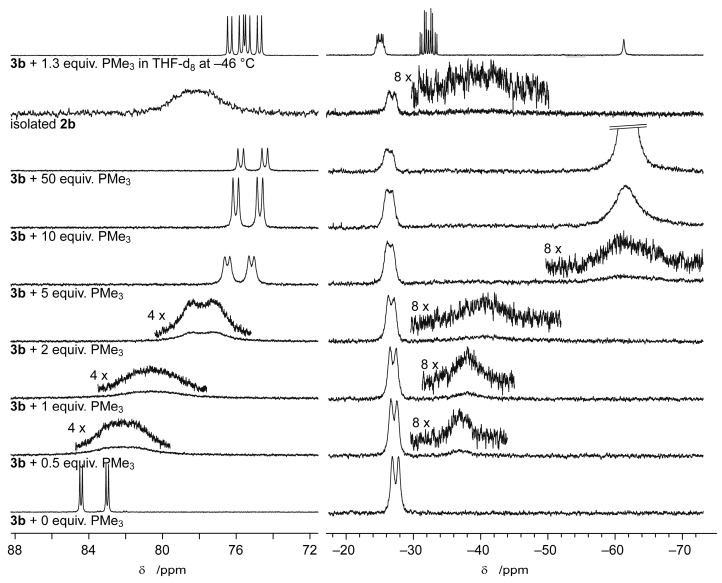
In situ ^31^P{^1^H} NMR spectra of the reaction of **3b** with different amounts of PMe_3_ (121.6 MHz, C_6_D_6_, rt), isolated **2b** and **3b** with 1.3 equiv. PMe_3_ at −46 °C (162.1 MHz, THF-d_8_).

The equilibrium between **2b** and **3b**, as a fundamental aspect of their coordination chemistry, was further studied via NMR spectroscopy. NMR titration of **3b** with increasing amounts of PMe_3_ shows a highly dynamic behaviour in the ^31^P{^1^H} NMR spectra at room temperature (Figure 4 and Figures S17–S19). Only one set of signals of the PBP ligand and the trans-B PMe_3_ ligand is observed, respectively. Whilst the ^31^P NMR signal of the PBP ligand changes appreciably from 84 ppm to 75 ppm with increasing amounts of PMe_3_ added, the signal of the trans-B PMe_3_ ligand, in **2b**, is only marginally influenced (−27.3 to −26.4 ppm). An additional signal is observed shifting from −37 ppm at low amounts of PMe_3_ to −62 ppm after the addition of an excess of PMe_3_. This is readily explained by a rapid exchange among **3b**, **2b** and free PMe_3_ on the NMR time scale and consequently, the observation of an averaged chemical shift of the exchanging PMe_3_ moieties throughout this process. In agreement with that, the spectrum observed for isolated **2b** is very virtually identical to the spectrum of **3b** after the addition of an equimolar amount of PMe_3_.

At low temperatures, however, the exchange among **3b**, **2b** and free PMe_3_ becomes slow on the NMR timescale, and well-resolved signals for **2b** and free PMe_3_ are observed (Figure 4,Figures S14 and S15). The ^31^P{^1^H} NMR spectrum of **2b** itself at −46 °C comprises three signals (A, M and N) of an A_2_MNX spin system with the expected ^31^P-^31^P and ^31^P-^103^Rh couplings (Figure S16, Table S3). Following the reaction of **3b** with different amounts of PMe_3_ via UV-Vis spectroscopy corroborates the rapid equilibrium between **3b** and **2b** being rather on the side of **3b** and free PMe_3_ (Figures S20 and S21).

In conclusion, it may be stated that the five-coordinate trigonal bipyramidal complex **2b**, in contrast to the Co analogue, easily dissociates one PMe_3_ ligand to give the distorted square planar complex **3b**. The virtual indifference in the ^31^P NMR chemical shift (and line shape) of the apparently not-exchanging trans-B PMe_3_ ligand around 27 ppm suggests that this exchange does not affect this ligand but involves only the equatorial PMe_3_ ligand. 

### 2.3. Iridium Complexes

**Scheme 5:**
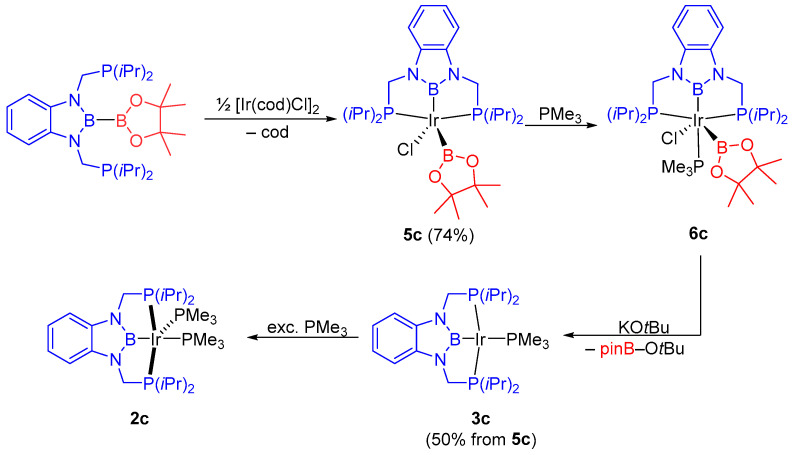
Consecutive formation of the PBP iridium boryl compels **5c**, **6c**, **3c** and **2c**.

Whilst for the formation of the cobalt and rhodium PBP pincer complexes **2a** and **2b**/**3b**, it may be arguable whether activation of the diborane precursor **1** proceeds via a σ bond metathesis or an oxidative addition/reductive elimination pathway, the reaction of **1** with the iridium(I) complex [Ir(cod)Cl]_2_ (cod = 1,5-cyclooctadien) to give the *bis*-boryl complex [(d(CH_2_P(*i*Pr)_2_)abB)Ir(Bpin)(Cl)] (**5c**) is obviously an oxidative addition reaction (Scheme 5). This five-coordinate complex reacts with excess PMe_3_ to give the six-coordinate complex [(d(CH_2_P(*i*Pr)_2_)abB)Ir(Bpin)(PMe_3_)(Cl)] (**6c**). 

Both complexes **5c** and **6c** crystallise in monoclinic space groups of the type *P*2_1_/*c*. The solid-state structure of **5c** contains one complex molecule in the asymmetric unit (Z = 4, Z’ = 1), whereas **6c** comprises two independent molecules in the asymmetric unit (Z = 8, Z’ = 2). The Bpin moiety in **5c** shows some positional disorder that is neglected in the further discussion; for **6c**, however, one of the independent molecules shows severe disorder and is not considered for further geometrical analysis (Supplementary Materials) [22].

**Figure 5:**
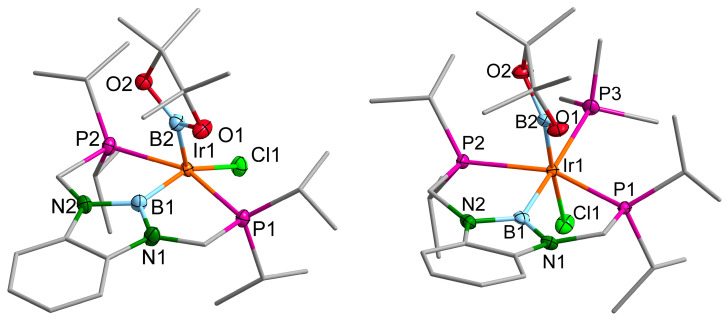
Molecular structures of the complexes [(d(CH_2_P(*i*Pr)_2_)abB)Ir(Bpin)(Cl)] (**5c**) (**left**, disorder omitted for clarity) and [(d(CH_2_P(*i*Pr)_2_)abB)Ir(Bpin)(Cl)(PMe_3_)] (**6c**) (**right**, one of two independent molecules shown) (Supplementary Materials) [22]. Selected distances [Å] and angles [°], **5c**: Ir1–B1 1.986(2), Ir1–B2 2.012(2), Ir1–P1 2.3354(4), Ir1–P2 2.3306(4), Ir1–Cl1 2.4144(4), P1–Ir1–P2 156.45(2), B1–Ir1–B2 72.16(8), B1–Ir1–Cl1 153.23(6), B2–Ir1–Cl1 134.49(6), ∠[P1,P2,B1,Ir1][B1,N1,N2,C_6_H_4_] 4.3(2), B1–B2 2.354(3); **6c**: Ir1–B1 2.052(4), Ir1–B2 2.050(4), Ir1–P1 2.3391(8), Ir1–P2 2.3627(9), Ir1–P3 2.4155(9), Ir1–Cl1 2.5667(9), P1–Ir1–P2 153.28(3), B1–Ir1–B2 78.9(1), B1–Ir1–Cl1 107.6(1), B1–Ir1–P3 172.6(1), B2–Rh1–Cl1 173.4(1), B2–Rh1–P3 94.0(1), ∠[P1,P2,P3,B1,Ir1][B1,N1,N2,C_6_H_4_] 24.78(8), B1–B2 2.605(2).

The trigonal bipyramidal geometry of **5c** may be considered typical for a five-coordinate Ir *bis*-boryl complex with phosphine ligands (Figure 5). All five structurally characterised complexes of this type adopt a trigonal bipyramidal geometry with the two phosphine ligands in the axial positions (P–Ir–P angle 157–172°, for PXP pincer ligands P–Ir–P angle 157–162°) and small B···B distances and B–Ir–B angles in the ranges of 2.22–2.41 Å and 65.8–76.7°, respectively [25,26,27,28,29]. 

In **6c**, the PMe_3_ ligand adopts a position trans to the PBP pincer boryl ligand, whilst the chlorido ligand occupies a position trans to the Bpin ligand (Figure 5). As a result, **6c** may best be described as a strongly distorted octahedral complex with the Bpin and chlorido ligand in the axial positions. Structurally, the extension of the coordination sphere to the distorted octahedral complex **6c** is accompanied by some ligand reorganisation. The P1–Ir–P2 angle reduces upon coordination by about 3° to deviate more from linearity, whereas the B–Ir–B angle deviates in **6c** by about 6° less from 90° than in **5c** (in accordance with the B···B distance increasing from **5c** to **6c** by 0.25 Å). The d(CH_2_P(*i*Pr)_2_)abB ligand backbone in **6c** (mean plane [B1,N1,N2,C_6_H_4_]) includes an angle of 24.8(8)° with the equatorial plane of the complex (mean plane [P1,P2,P3,B1,Ir1]), 20° more than in the five-coordinate **5c**. This is a result of the increased steric encumbrance induced by the extension of the coordination sphere in **6c**. The B–Ir distance increases slightly upon PMe_3_ coordination in **6c** because of the presence of trans ligands. This is more significant for B1, which is trans to the stronger trans influencing ligand PMe_3_ as opposed to the chlorido ligand for B2. The Cl–Ir distance increases accordingly, whereas the P1/P2–Ir1 distances remain virtually unaffected. Again, because of the strong trans influence of the boryl ligand, the P–Ir distance of the PMe_3_ ligand is longer than those of the pincer phosphine atoms by about 0.06 Å [30]. 

The solution-state ^1^H, ^31^P and ^13^C NMR spectroscopic data for **5c** and **6c** fulfil the expectations and can readily be explained by the solid-state structures. Surprising, however, are the ^11^B NMR chemical shifts. For both complexes, two very distinct, somewhat broadened singlets at chemical shifts of 39.7 ppm (Δ*w_½_* = 340 Hz) and 19.9 ppm (Δ*w_½_* = 330 Hz) for **5c** and of 48.8 ppm (Δ*w_½_* = 460 Hz) and 26.6 ppm (Δ*w_½_* = 450 Hz) for **6c** are observed in THF-d_8_ at room temperature. This chemical shift range is somewhat different from the ^11^B NMR data for the reported Ir(III) boryl in a range of 29–35 ppm for five-coordinate and of 30–43 ppm for six-coordinate complexes, respectively [1,2,7,25,26,28,31].

**Figure 6:**
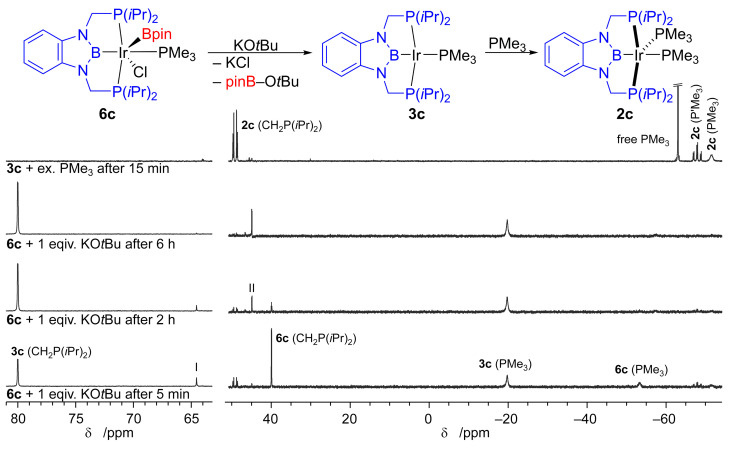
In situ ^31^P{^1^H} NMR spectra of the reaction of **6c** with KO*t*Bu (121.6 MHz, THF-d_8_, rt).

Whilst complex **6c** is stable under inert conditions, it reacts readily with an equimolar amount of KO*t*Bu to give the Ir(I) PBP pincer complex **3c** (Figure 6, Scheme 5). Monitoring this reaction via in situ NMR spectroscopy (Figure 6 and Figure S34–S36) shows an essentially clean conversion to **3c**, as indicated by its characteristic signals around 80 ppm (doublet, *J_P–P_* = 5 Hz) for the pincer phosphorus atoms and a broadened singlet for the PMe_3_ ligand around –20 ppm (Figure S35 Supplementary Materials) [22]. Only minor amounts of a so far unidentified side product with a ^31^P NMR signal at 45 ppm (II) are observed. However, upon closer evaluation, two transient species are observed during this reaction. At one hand side, the five-coordinate complex **2c** (vide infra) is formed in small amounts in the beginning but is later on fully consumed (Figure 6). On the other side, a species with a ^31^P{^1^H} NMR singlet signal at 64.5 ppm (I) is observed. In agreement with this, the ^11^B NMR data suggest the presence of a transient boryl intermediate at 40 ppm, whereas **3c** itself exhibits a moderately broad ^11^B{^1^H} NMR signal around 56 ppm (Figure S36 Supplementary Materials) [22,32]. It may be assumed that the conversion of **6c** to **3c** proceeds via the initial coordination of a O*t*Bu ligand followed by (possibly after some reorganisation) the reductive elimination to an Ir(I) PBP complex, **3c** or a closely related species. The intermediate presence of **2c** may be explained by the intermediate liberation of PMe_3_ and its transient addition to **3c** during this process. An in situ ^31^P{^1^H} NMR spectrum of a mixture of **3c** and excess PMe_3_ corroborates the facile formation of **2c** (Figure 6, top). Moreover, it must be emphasised that the system **2c**/**3c**/PMe_3_ exhibits much less dynamic behaviour than the homologous rhodium system **2b**/**3b**/PMe_3_ (vide supra). Contrary to the latter, even in the presence of excess PMe_3_ at room temperature, a well-resolved, ^31^P{^1^H} NMR spectrum (A_2_MN spin system) with a narrow linewidth is observed, indicating only comparably slow exchange of a coordinated PMe_3_ ligand with free PMe_3_. Contrary to **2b**, distinct signals for both PMe_3_ ligands are observable for **2c** at room temperature in the presence of free PMe_3_ (Figure 6, top). One of these signals (around −70 ppm), however, sharpens upon only moderate cooling to an apparent quartet (Figure S27 Supplementary Materials) [22]. In agreement with that, in situ UV-Vis spectroscopic data of **3c** in the presence of different amounts of PMe_3_ indicate a rapid equilibration, rather on the side of **2c** (Figures S30 and S31). The ^11^B NMR shift of **2c** of around 55 ppm is virtually unaffected by the change in the coordination number.

In conclusion, it may be stated that the five-coordinate trigonal bipyramidal complex **2c**, similarly to the cobalt analogue **2a** and opposed to the rhodium homologue, shows only little dynamic behaviour in solution and does not readily dissociate a PMe_3_ ligand to give the distorted square planar complex **3c**. However, gas-phase DFT computational data suggest similar thermodynamic data for the dissociation of PMe_3_ from **2c** (Δ*E_0_* = 16 kJ mol^−1^, Δ*G_298_* = −48 kJ mol^−1^) as for the rhodium analogue **2b** (Supplementary Materials) [22].

**Figure 7:**
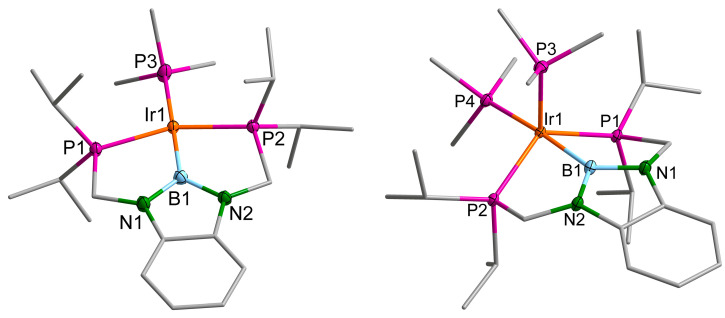
Molecular structures of the complexes [(d(CH_2_P(*i*Pr)_2_)abB)Ir–PMe_3_] (**3c**) and (**left**) [(d(CH_2_P(*i*Pr)_2_)abB)Ir(PMe_3_)_2_] (**2c**) (**right**). Selected distances [Å] and angles [°], **3c**: Ir1–B1 2.034(2), Ir1–P1 2.2764(4), Ir1–P2 2.2662(2), Ir1–P3 2.3355(5), P1–Ir1–P2 152.95(2), B1–Ir1–P3 176.73(6), Ir1–[P1,P2,P3,B1] 0.0386(3); **2c**: Ir1–B1 2.055(3), Ir1–P1 2.3113(6), Ir1–P2 2.3165(6), Ir1–P3 2.2911(6), Ir1–P4 2.3521(6), P1–Ir1–P2 127.71(2), P2–Ir1–P3 108.84(8), P1–Ir1–P3 111.21(2), B1–Ir1–P4 172.02(8), Ir1–[P1,P2,P3] 0.4666(3).

The complexes **2c** and **3c** crystallise isostructurally with the homologous rhodium complexes in monoclinic space groups of the types *P*2_1_/*n* and *P*2_1_/*c*, respectively (Z = 4, Z’ = 1) (Supplementary Materials) [22]. As a consequence, the molecular structure of **2c** (Figure 7, right) differs only marginally form the structure of the lighter homologue **2b** and from the cobalt homologue **2a** (Figure S38). 

The sum of angles in the equatorial plane [P1,P2,P3] of the distorted trigonal bipyramidal complex **2c** is, with 347.76°, only insignificantly different from that in **2a** and **2b**. The angle P1–Ir1–P2, involving the pincer phosphorus atoms, is larger than that in **2a** by about 2° and, hence, virtually identical to that in **2b**. The displacement of Ir1 from the [P1,P2,P3] plane is in the middle between the values for two lighter homologues, by 0.03 Å larger than in **2a** and by 0.02 Å smaller than in **2b**. Generally, the M–P distances, however, increase from **2a** to **2b** and **2c** by about 0.12 Å, most significantly between the cobalt and the rhodium complex. 

Overall, the PBP pincer ligand shows, within the series **2a**, **2b 2c**, a high ability to coordinate different metal ions. The high flexibility of this ligand is also illustrated by a comparison of the five-coordinate complexes **5c** and **2c**. For both complexes, a trigonal bipyramidal geometry is observed; however, whilst in **2c**, the phosphorus atoms of the PBP pincer ligand occupy two equatorial positions and the boryl moiety is bound in an axial position, in **5c**, two phosphorus atoms coordinate in the two axial positions and the boron atom in an equatorial position. This is illustrated by P–M–P angles included by the pincer phosphorus atoms decreasing by 30° from **5c** to **2c**. 

The solid-state structure of the distorted square planar complex **3c** is again very similar to that of its rhodium homologue **3b** (Figure S39) with a nearly linear B1–Ir1–P3 angle and a P1–Ir1–P2 angle of 152.95(2)° deviating significantly from linearity. Noteworthy is the Ir1–B1 distance in **3c** that is slightly (0.01 Å) longer, whereas the pincer P–M distances are identical, and the trans-B P–M distance is slightly shorter (0.02 Å) than the respective distance in the rhodium homologue **3b**.

## 3. Discussion

A series of either group 9 PBP diaminoboryl pincer complexes was synthesised using the unsymmetrical diborane(4) **1** as a PBP pincer precursor and fully characterised. In an extension of our earlier work [15], this exemplifies again the versatility of this compound as a PBP pincer ligand precursor. The Co^I^ and Rh^I^ complexes [(d(CH_2_P(*i*Pr)_2_)abB)Co–(PMe_3_)_2_] (**2a**) and [(d(CH_2_P(*i*Pr)_2_)abB)Rh–(PMe_3_)_n_] (**2b** (n = 2), **3b** (n = 1)), respectively, were obtained in a one-step reaction from the respective Co^I^ and Rh^I^ precursors (Scheme 2 and Scheme 4). Whilst an oxidative addition/reductive elimination pathway is, for both reactions, feasible, in the rhodium case, a σ bond metathesis pathway may be feasible, considering results based on a related non-pincer ligand [20]. The heavier Ir^I^ homologue, however, was obtained via the isolated intermediate Ir^III^ complexes [(d(CH_2_P(*i*Pr)_2_)abB)Ir(Bpin)(Cl)] (**5c**) and [(d(CH_2_P(*i*Pr)_2_)abB)Ir(Bpin)(PMe_3_)(Cl)] (**6c**). Complex **5c** is formed upon an oxidative addition reaction of the diborane(4) **1** with [Ir(cod)Cl]_2_ (cod = 1,5-cyclooctadien) and subsequently reacts via PMe_3_ addition to **6c**. The coordination chemistry of the resulting homologous complexes [(d(CH_2_P(*i*Pr)_2_)abB)M(PMe_3_)_2_] (**2a**–**c**) and [(d(CH_2_P(*i*Pr)_2_)abB)M–PMe_3_] (**3b**,**c**) was studied structurally in the solid state, as well as spectroscopically in solution. However, for M = Rh and Ir, both complexes are structurally very similar but differ in the dynamic behaviour and the relative accessibility of the four (**3b**,**c**) vs. the five (**2b**,**c**) coordinated complexes. For Co only the five-coordinate complex **2a** is accessible, whereas Lewis acid-promoted PMe_3_ abstraction under a dinitrogen atmosphere leads to the formation of the surprisingly stable N_2_ complex [(d(CH_2_P(*i*Pr)_2_)abB)Co–(N_2_)(PMe_3_)] (**4a**). 

Having, with the unsymmetrical diborane(4) [(d(CH_2_P(*i*Pr)_2_)abB)–Bpin] (**1**), a well accessible and versatile PBP ligand precursor that is capable of oxidative addition (Pt^II^, Co^I^, Rh^I^ (possibly), Ir^I^) and σ bond metathesis (Cu^I^ and possibly Rh^I^) reactions [15,20] will stimulate the further development of PBP pincer ligands. In conclusion, PBP diaminoboryl pincer ligands are a ligand class with remarkable ligand properties with respect to their high σ donor strength and weak π acceptor properties—leading to a strong trans effect and influence [30]—that provide stability for the inherently reactive B–M bond due to their pincer framework. Furthermore, PBP pincer ligands are tuneable based on the backbone and P atoms substituents, making them interesting for a broad range of applications from catalysis to the stabilisation of reactive intermediates. 

## 4. Materials and Methods

### 4.1. General Considerations

pinB–B(d(CH_2_P(*i*Pr)_2_)ab) (**1**), [(Me_3_P)_4_CoMe], [(Me_3_P)_3_RhCl] and [(cod)IrCl]_2_ were prepared according to literature procedures [15,16,17,18,33]. All other compounds were commercially available and were used as received; their purity and identity were checked using appropriate spectroscopic methods. Unless otherwise noted, all solvents were dried using an MBraun solvent purification system, deoxygenated using the freeze-pump-thaw method and stored under purified nitrogen. Unless noted otherwise, all manipulations were performed using standard Schlenk techniques under an atmosphere of purified nitrogen or in a nitrogen-filled glove box (MBraun). NMR spectra were recorded on Bruker Avance II 300, Avance III HD 300 and Avance III 400 spectrometers. NMR tubes equipped with screw caps (WILMAD) were used, and the solvents were dried over potassium/benzophenone and degassed. Chemical shifts (*δ*) are given in ppm, using the (residual) resonance signal of the solvents for calibration (C_6_D_6_: ^1^H NMR: 7.16 ppm, ^13^C NMR: 128.06 ppm; PhMe-d_8_: ^1^H NMR: 2.08 ppm, ^13^C NMR: 20.43 ppm; THF-d_8_: ^1^H NMR: 1.72 ppm, ^13^C NMR: 25.31 ppm) [34]. ^11^B and ^31^P NMR chemical shifts are reported relative to pseudo external BF_3_·Et_2_O and 85% H_3_PO_4(aq)_, respectively. ^13^C{^1^H}, ^11^B{^1^H} and ^31^P{^1^H} NMR spectra were recorded employing composite pulse ^1^H decoupling. ^11^B NMR spectra were processed applying a back linear prediction, in order to suppress the broad background signal due to the boron in the NMR tube and instrument. A Lorentz-type window function (LB = 10 Hz) was used, and the spectra were carefully evaluated to ensure that no genuinely broad signals of the sample were suppressed. Simulations were conducted with the TOPSPIN/DAISY program package (Bruker). Melting points were determined in flame-sealed capillaries under nitrogen using a Büchi 535 apparatus and are not corrected. Elemental analyses were performed at the Institut für Anorganische und Analytische Chemie of the Technische Universität Braunschweig using an Elementar vario MICRO cube instrument. A Bruker Vertex 70 spectrometer was used for recording IR spectra. The IR spectra were recorded in PhMe solutions in a cuvette of an approximately 1 mm optical path length equipped with NaCl windows.

X-ray Structure Determination. The single crystals were transferred into inert perfluoroether oil inside a nitrogen-filled glovebox and, outside the glovebox, rapidly mounted on top of a CryoLoop (Hampton Research) and placed on the diffractometer in the cold nitrogen gas stream of a Cryostream 800 cooling system (Oxford Cryosystems) [35]. The data were collected on a Rigaku Oxford Diffraction Synergy-S instrument using either mirror-focused MoKα or CuKα radiation (Rigaku PhotonJet microfocus sources). The reflections were indexed and integrated, and appropriate absorption corrections were applied as implemented in the CrysAlisPro software package [36]. The structures were solved employing the program SHELXT and refined anisotropically for all non-hydrogen atoms via full-matrix least squares based on all F^2^ values using SHELXL software [37,38,39]. Generally, hydrogen atoms were refined employing a riding model; methyl groups were treated as rigid bodies and were allowed to rotate about the E–CH_3_ bond. During refinement and analysis of the crystallographic data, the programs OLEX^2^, PLATON, Mercury and Diamond were used [40,41,42,43]. Unless noted otherwise non-C,H atoms are depicted as ellipsoids at the 50% probability level, whereas the carbon atom framework is depicted as a stick model (grey), and hydrogen atoms are omitted for clarity. Adapted numbering schemes may be used to improve the readability. Further crystallographic details can be found in the Supplementary Materials available.

### 4.2. Experimental Procedures and Analysis Data

#### 4.2.1. [(d(CH_2_P(*i*Pr)_2_)abB)Co(PMe_3_)_2_] (**2a**)

In a Schlenk-flask, d(CH_2_P(*i*Pr)_2_)abB–Bpin (**1**) (100 mg, 0.198 mmol, 1 equiv.) and [(Me_3_P)_4_CoMe] (75 mg, 0.198 mmol, 1 equiv.) were dissolved in toluene (50 mL) and stirred for 24 h at 50 °C whilst a reduced pressure was applied for about 50% of the time (the pressure was normalised overnight). The solvent was completely removed in vacuo and the brown residue was dissolved in *n*-pentane and recrystallised at −40 °C. The resulting dark orange crystals were washed with cold *n*-pentane (1 mL) and dried in vacuo (77 mg, 0.131 mmol, 66%). 

**^1^H NMR (PhMe-d_8_**, **400.4 MHz**, **rt)** *δ* = 6.96–6.91 (m, 2 H, 3-*H*C_Ar_), 6.74–6.79 (m, 2 H, 2-*H*C_Ar_), 3.50 (d, *^2^J_H-H_* = 11.0 Hz, 2 H, C*H*H’), 3.47 (d, *^2^J_H-H_* = 11.0 Hz, *^2^J_H-P_* = 4 Hz, 2 H, CH*H*’), 1.98 (app. sept., *^3^J_H-H_* = 7.6 Hz, *^3^J_H-H_* = 7.0 Hz, 2 H, C*H*(CH_3_)_2_), 1.89 (m, *^3^J_H-H_* = 7.0 Hz, *^3^J_H-H_* = 7.4 Hz, *J_H-P_* = 2.5, 4.0 Hz, 2 H, C’*H*(CH_3_)_2_), 1.29 (d, *^2^J_H-P_* = 5.0 Hz, 9 H, P_ap_(C*H*_3_)_3_), 1.23 (app. q, *^3^J_H-H_* = 7.6 Hz, *J_H-P_* = 6.8, 6.0 Hz, 6 H, CH(C*H*_3_)(C’H_3_)), 1.11 (m, *^3^J_H-H_* = 7.0 Hz, *J_H-P_* = 5.7, 3.6 Hz, 6 H, CH(CH_3_)(C’*H*_3_)), 1.04 (d, *^2^J_H-P_* = 4.8 Hz, 9 H, P_eq_(C*H*_3_)_3_), 0.98 (app. q, *^3^J_H-H_* = 7.0 Hz, *J_H-P_* = 6.9, 6.5 Hz, 6 H, C’H(C*H*_3_)(C’H_3_)), 0.78 (app. q, *^3^J_H-H_* = 7.4 Hz, *J_H-P_* = 6.5, 5.7 Hz, 6 H, C’H(CH_3_)(C’*H*_3_)). **^13^C{^1^H} NMR (PhMe-d_8_**, **100.7 MHz**, **rt)** *δ* = 140.6 (app. t, *J_C-P_* = 6 Hz, 1-*C*_Ar_), 117.2 (s, 3-H*C*_Ar_), 106.5 (s, 2-H*C*_Ar_), 44.5 (m, *C*HH’), 32.0 (app. dt, *J_C-P_* = 19, 3 Hz, *C*H(CH_3_)_2_), 29.1 (app. t, *J_C-P_* = 5 Hz, *C’*H(CH_3_)_2_), 27.8 (m, P_ap_(*C*H_3_)_3_), 25.7 (app. dq, *J_C-P_* = 15, 4 Hz, P_eq_(*C*H_3_)_3_), 21.9 (s, CH(*C*H_3_)(C’H_3_)), 20.3 (s, CH(CH_3_)(*C’*H_3_)), 19.4 (s, C’H(*C*H_3_)(C’H_3_)), 18.9 (app. t, *J_C-P_* = 3 Hz, C’H(C*H*_3_)(*C’*H_3_)). **^31^P{^1^H} NMR (PhMe-d_8_**, **162.1 MHz**, **rt)** *δ* = 83.0 (br. s, Δ*w_½_* = 217 Hz, CH_2_*P*(*i*Pr)_2_), −1.2 (br. s, Δ*w_½_* = 132 Hz, *P*(CH_3_)_3_), −17.4 (br. s, Δ*w_½_* = 245 Hz, *P*(CH_3_)_3_). **^11^B{^1^H} NMR (PhMe-d_8_**, **128.5 MHz**, **rt)** *δ* 57.0 (br. s, Δ*w_½_* = 360 Hz). **^1^H NMR (PhMe-d_8_**, **400.4 MHz**, **−69 °C)** *δ* = 7.20–7.14 (m, 2 H, C_Ar_), 6.95–6.89 (m, 2 H, *H*C_Ar_), 3.51–3.33 (m, 4 H, C*HH’*), 1.92 (br. s, 2 H, C*H*(CH_3_)_2_), 1.80 (br. app. sept., *J_H-H_* = 7 Hz, 2 H, C’*H*(CH_3_)_2_), 1.22 (d, *^2^J_H-P_* = 5 Hz, 9 H, P(C*H*_3_)_3_), 1.20 (br. s, 6 H, CH(C*H*_3_)(C’H_3_)), 1.06 (d, *^2^J_H-P_* = 5 Hz, 9 H, P(C*H*_3_)_3_), 1.03 (br. s, 6 H, CH(CH_3_)(C’*H*_3_)), 0.99–0.90 (m, 6 H, C’H(C*H*_3_)(C’H_3_)), 0.75 (br. s, 6 H, C’H(CH_3_)(C’*H*_3_)). **^31^P{^1^H} NMR (PhMe-d_8_**, **162.1 MHz**, **−69 °C)** *δ* = 82.9 (dd, *^2^J_P-P_* = 80, 30 Hz, CH_2_*P*(*i*Pr)_2_), 0.7 (app. q, *^2^J_P-P_* = 30, 28 Hz, P_ap_(CH_3_)_3_), −16.2 (td, q, *^2^J_P-P_* = 80, 28 Hz, *P*_eq_(CH_3_)_3_). **^1^H NMR (C_6_D_6_**, **300.1 MHz**, **rt)** *δ* = 7.10–7.03 (m, 2 H, *H*C_Ar_), 6.92–6.85 (m, 2 H, *H*C_Ar_), 3.50 (m, 4 H, C*HH*’), 2.06–1.85 (m, 4 H, C*H*(CH_3_)_2_), 1.29 (d, *^2^J_H-P_* = 5.0 Hz, 9 H, P(C*H*_3_)_3_), 1.28–1.19 (m, 6 H, CH(C*H*_3_)(C’H_3_)), 1.14–1.07 (m, 6 H, CH(C*H*_3_)(C’H_3_)), 1.07 (d, *^2^J_H-P_* = 4.8 Hz, 9 H, P(C*H*_3_)_3_), 1.03–0.94 (m, 6 H, C’H(C*H*_3_)(C’H_3_)), 0.87–0.76 (m, 6 H, C’H(CH_3_)(C’*H*_3_)). **^11^B{^1^H} NMR (C_6_D_6_**, **96.3 MHz**, **rt)** *δ* 57.2 (br. s, Δ*w_½_* = 460 Hz). **^31^P{^1^H} NMR (C_6_D_6_**, **121.5 MHz**, **rt)** *δ* 82.9 (br. s, Δ*w_½_* = 200 Hz, CH_2_*P*(*i*Pr)_2_), −1.5 (br. s, Δ*w_½_* = 135 Hz, P(CH_3_)_3_), −17.5 (br. s, Δ*w_½_* = 240 Hz, *P’*(CH_3_)_3_). **Anal. Calcd. for C_26_H_54_BCoN_2_P_4_ (2a):** C, 53.08; H, 9.25; N, 4.76. Found: C, 52.84; H, 9.27; N, 5.13. **m.p.:** 160–163 °C.

#### 4.2.2. [(d(CH_2_P(*i*Pr)_2_)abB)Co(N_2_)(PMe_3_)] (**4a**)

Single crystals of **4a:** In a nitrogen-filled glovebox, **2a** (10 mg, 17 µmol, 1 equiv.) and triphenylborane (4.1 mg, 17 µmol, 1 equiv.) were dissolved in C_6_D_6_ (0.7 mL). After 3 d at room temperature, the solution was layered with *n*-pentane. Colourless crystals of Me_3_P–BPh_3_ separated. The supernatant solution was decanted, and the solvent was removed in vacuo. The residue was dissolved in toluene (0.5 mL), and the solution was layered with *n*-pentane and cooled to −40 °C. Colourless crystals formed overnight, from which the supernatant solution was decanted and cooled again to −40 °C. A few orange single crystals of **4a** suitable for x-ray diffraction were obtained from this solution. In situ IR characterisation of **4a** was as follows: in a nitrogen-filled glovebox, **2a** (10 mg, 17 µmol, 1 equiv.) and tris(pentafluorophenyl)borane (8.7 mg, 17 µmol, 1 equiv.) were dissolved in toluene (0.4 mL) and transferred into an IR cuvette. An IR spectrum of this solution was recorded. The reaction under an Ar atmosphere was conducted analogously in an Ar-filled glovebox. In situ NMR characterisation of **4a** was performed as follows: in a nitrogen-filled glovebox, **2a** (16.1 mg, 27 µmol, 1 equiv.) and tris(pentafluorophenyl)borane (14 mg, 27 µmol, 1 equiv.) were dissolved in toluene-d_8_ and filtered through a small pad of celite. NMR spectra of this solution were recorded.

**^1^H NMR (PhMe-d_8_**, **400.4 MHz**, **rt)** *δ* = 6.88 (br. s, 2 H, *H*C_Ar_), 6.66 (br. s, 2 H, *H*C_Ar_), 3.50 (br. s, 2 H, C*H*_2_), 3.34 (br. s, 2 H, C*H*_2_), 2.11 (overlapping with the residual solvent signal, C*H*(CH_3_)_2_), 1.48–0.65 (P(C*H*_3_)_3_) and CH(C*H*_3_)_2_). **^31^P{^1^H} NMR (PhMe-d_8_**, **162.1 MHz**, **rt)** *δ* = 93.5 (br. s, Δ*w_½_* = 211 Hz, CH_2_*P*(*i*Pr)_2_), −13.4 (br. s, Co–*P*(CH_3_)_3_). **^11^B{^1^H} NMR (PhMe-d_8_**, **128.5 MHz**, **rt)** *δ* 54.3 (br. s, Δ*w_½_* = 630 Hz). **^1^H NMR (PhMe-d_8_**, **400.4 MHz**, **−69 °C)** *δ* = 6.77 (br. s, 2 H, *H*C_Ar_), 3.35 (br. s, 2 H, C*H*_2_), 3.13 (br. d, 2 H, C*H*_2_), 1.95 (br. s, 4 H, C*H*(CH_3_)_2_), 1.40 (br. s, 6 H, CH(C*H*_3_)_2_), 1.19 (br. s, 6 H, CH(C*H*_3_)_2_), 1.04 (br. s, 15 H, CH(C*H*_3_)_2_) and P(C*H*_3_)_3_), 0.80 (br. s, 6 H, CH(C*H*_3_)_2_). **^31^P{^1^H} NMR (PhMe-d_8_**, **162.1 MHz**, **−80 °C)** *δ* = 94.6 (d, *^2^J_P-P_* = 74 Hz, CH_2_*P*(*i*Pr)_2_), −11.4 (t, *^2^J_P-P_* = 74 Hz, Co–*P*(CH_3_)_3_). 

#### 4.2.3. [(d(CH_2_P(*i*Pr)_2_)abB)Rh(PMe_3_)_2_] (**2b**)

The reaction was performed as described for **3b** on a 55 μmol scale (vide infra). After filtration, an excess of PMe_3_ (30 µL, 22 mg, 0.3 mmol, 5.5 equiv.) was added, and the resulting yellow solution was cooled to −40 °C. After 48 h, bright yellow crystals suitable for X-ray crystallography had formed. The supernatant solution was decanted, and the crystals were dried in vacuo (15 mg, 24 μmol, 43%). NMR spectra of the isolated material show an equilibrium among **2b**, **3b** and free PMe_3_ (Figures S17–S19). NMR spectra of **2b** were recorded from a solution of **3b** (15 mg, 28 μmol) in THF-d_8_ (0.7 mL) after the addition of PMe_3_ (3.7 µL, 2.7 mg, 37 μmol, 1.3 equiv.).

**^1^H NMR (THF-d_8_**, **400.4 MHz**, **−46° C)** *δ* 6.58–6.47 (m, 4 H, 2,3-*H*C_Ar_), 3.61–3.42 (m, 4 H, C*H*_2_), 2.12 (app. sept., *J* = 6.9 Hz, 2 H, C*H*(CH_3_)_2_), 1.64 (br. s, 2 H, Δ*w_½_* = 25 Hz, C*H*(CH_3_)_2_), 1.43 (d, *^2^J_H-P_* = 4.8 Hz, 9 H, P(C*H*_3_)_3_), 1.34–1.21 (m, 12 H, CH(C*H*_3_)_2_), 1.11 (d, *^2^J_H-P_* = 4.8 Hz, 9 H, P’(C*H*_3_)_3_), 1.03 (app. q, *J* = 5.6 Hz, 6 H, CH(C*H*_3_)_2_), 0.94 (d, *^2^J_H-P_* = 2 Hz, 2.9 H, free P(C*H*_3_)_3_), 0.7 (app. q, *J* = 7.1 Hz, 6 H, CH(C*H*_3_)_2_). **^11^B{^1^H} NMR (THF-d_8_**, **128.5 MHz**, **rt)** *δ* 55.4 (s, Δ*w_½_* = 365 Hz). **^31^P{^1^H} NMR (THF-d_8_**, **162.1 MHz**, **rt)** *δ* 76.6 (br. d, *^1^J_P-Rh_* = 153 Hz, Δ*w_½_* = 150 Hz, CH_2_*P*(*i*Pr)_2_), −26.4 (br. d, ^1^*J_P-Rh_* = 97 Hz, Δ*w_½_* = 105 Hz, *P*(CH_3_)_3_), −37 (br. s, Δ*w_½_* = 1000 Hz, *P*(CH_3_)_3_), −54 (br. s, Δ*w_½_* = 1600 Hz, free *P*(CH_3_)_3_). **^31^P{^1^H} NMR (THF-d_8_**, **162.1 MHz**, **−46 °C)** *δ* 75.5 (ddd, *^1^J_P-Rh_* = 157 Hz, *^2^J_P-P_* = 38, 103 Hz, CH_2_*P*(*i*Pr)_2_), −25.1 (app. dq, ^1^*J_P-Rh_* = 105 Hz, ^2^*J_P-P_* = 43, 38 Hz, *P_ap_*(CH_3_)_3_), −32.3 (dtd, ^1^*J_P-Rh_* = 157 Hz, ^2^*J_P-P_* = 103, 43 Hz, *P_eq_*(CH_3_)_3_). **Anal. Calcd. for C_26_H_54_BN_2_P_4_Rh (2b):** C, 49.39; H, 8.61; N, 4.30. Found: C, 48.91; H, 8.56; N, 4.47. 

#### 4.2.4. [(d(CH_2_P(*i*Pr)_2_)abB)Rh(PMe_3_)] (**3b**)

In a nitrogen-filled glovebox, d(CH_2_P(*i*Pr)_2_)abB–Bpin (**1**) (41 mg, 82 μmol, 1 equiv.) and [Rh(PMe_3_)_3_Cl] (30 mg, 82 μmol, 1 equiv.) were combined and dissolved in toluene (10 mL). A solution of KO*t*Bu (9 mg, 82 μmol, 1 equiv.) in THF (2 mL) was added, and the bright orange solution was stirred for 5 min at room temperature. The solvent was removed in vacuo. The residue was extracted with *n*-pentane (2 × 3.5 mL) and filtered through a pad of celite. The solvent was removed in vacuo. The orange residue was recrystallised from diethyl ether (3 mL) at −40 °C to give bright orange crystals of [(d(CH_2_P(*i*Pr)_2_)abB)Rh(PMe_3_)] (**3b**) (30 mg, 56 μmol, 70%).

**^1^H NMR (THF-d_8_**, **400.4 MHz**, **rt)** *δ* 6.72–6.67 (m, 2 H, *H*C_Ar_), 6.67–6.62 (m, 2 H, *H*C_Ar_), 3.64 (app. t, *J* = 2, 2 Hz, 4 H, NCH_2_P), 2.18 (app. sept., *J* = 7, 6, 1.5, 1.5 Hz, 4 H, C*H*(CH_3_)_2_), 1.39 (dd, ^2^*J*_H-P_ = 4.3, ^3^*J*_H-Rh_ = 0.6 Hz, 9 H, P(C*H*_3_)_3_), 1.19 (app. q, *J* = 7.0, 7.4, 7.4 Hz, 12 H, CH(C*H*_3_)_2_), 1.09 (app. q, *J* = 6.0, 6.3, 6.3 Hz, 12 H, CH(C*H*_3_)_2_). **^13^C{^1^H} NMR (THF-d_8_**, **101.7 MHz**, **rt)** *δ* 140.7 (app. td, *J_C-P_* = 9 Hz, *J_C-Rh_* = 1.5 Hz, 1-*C*_Ar_), 117.3 (s, H*C*_Ar_), 104.9 (s, H*C*_Ar_), 43.9 (m, *C*H_2_), 28.6 (app. t, *J_C-P_* = 8.5 Hz, *C*H(C*H*_3_)(*C’*H_3_)), 23.0 (app. dtd, *J_C-P_* = 13, 3 Hz, ^2^*J_C-Rh_* = 1 Hz, P(*C*H_3_)_3_), 20.8 (app. t, *J_C-P_* = 5 Hz, CH(*CH*_3_)(C’H_3_)), 20.8 (br. s, CH(C*H*_3_)(*C’*H_3_)). **^11^B{^1^H} NMR (THF-d_8_**, **128.5 MHz**, **rt)** *δ* 52.4 (s, Δ*w_½_* = 400 Hz). **^31^P{^1^H} NMR (THF-d_8_**, **162.1 MHz**, **rt)** *δ* 84.1 (dd, *^1^J_P-Rh_* = 173 Hz, *^2^J_P-P_* = 17 Hz, CH_2_*P*(*i*Pr)_2_), −27.1 (br. d, *^1^J_P-Rh_* = 111 Hz, Δ*w_½_* = 90 Hz, *P*(CH_3_)_3_). **^31^P{^1^H} NMR (THF-d_8_**, **162.1 MHz**, **−102 °C)** *δ* 83.8 (dd, *^1^J_P-Rh_* = 171 Hz, *^2^J_P-P_* = 17 Hz, CH_2_*P*(*i*Pr)_2_), −25.4 (dt, ^1^*J_P-Rh_* = 113 Hz, ^2^*J_P-P_* = 17 Hz, *P*(CH_3_)_3_). **Anal. Calcd. for C_23_H_45_BN_2_P_3_Rh (3b):** C, 49.66; H, 8.15; N, 5.04. Found: C, 49.43; H, 8.11; N, 5.38. **m.p.:** 199–200 °C.

#### 4.2.5. [(d(CH_2_P(*i*Pr)_2_)abB)Ir(PMe_3_)_2_] (**2c**))

In a nitrogen-filled glovebox, **3c** (30 mg, 46 μmol, 1 equiv.) was dissolved in THF (5 mL), and PMe_3_ (23.6 µL, 17.7 mg, 0.23 mmol, 5 equiv.) was added. The solvent was removed under in vacuo conditions. The light yellow residue was recrystallised from diethyl ether (2 mL) at −40 °C to give light yellow crystals of [(d(CH_2_P(*i*Pr)_2_)abB)Ir(PMe_3_)_2_] (**2c**) (7 mg, 9.7 µmol, 21%).

**^1^H NMR (PhMe-d_8_**, **400.4 MHz**, **rt)** *δ* 6.95–6.89 (m, 2 H, 3-*H*C_Ar_), 6.83–6.77 (m, 2 H, 2-*H*C_Ar_), 3.53–3.39 (m, 4 H, C*H*_2_), 1.96 (app. br. sept., *J* = 7 Hz, 2 H, C*H*(CH_3_)_2_), 1.63 (br. s, 2 H, Δ*w_½_* = 25 Hz, C’*H*(CH_3_)_2_), 1.51 (d, *^2^J_H-P_* = 5.9 Hz, 9 H, P(C*H*_3_)_3_), 1.24 (d, *^2^J_H-P_* = 6.3 Hz, 9 H, P’(C*H*_3_)_3_), 1.20–1.08 (m, 12 H, CH(C*H*_3_)_2_), 0.89 (app. q, *J* = 6.9 Hz, 6 H, C’H(C*H*_3_)(C’H_3_)), 0.64 (app. q, *J* = 7.1 Hz, 6 H, C’H(CH_3_)(C’*H*_3_)). **^11^B{^1^H} NMR (PhMe-d_8_**, **128.5 MHz**, **rt)** *δ* 55.2 (s, Δ*w_½_* = 475 Hz). **^31^P{^1^H} NMR (PhMe-d_8_**, **162.1 MHz**, **−35 °C)** *δ* 50.7 (dd, *^2^J_P-P_* = 27, 111 Hz, CH_2_*P*(*i*Pr)_2_), −65.7 (td, ^2^*J_P-P_* = 27, 111 Hz, *P’*(CH_3_)_3_), −69.9 (app br. q, ^2^*J_P-P_* = 27, 27 Hz, *P*(CH_3_)_3_). **^31^P{^1^H} NMR (PhMe-d_8_**, **162.1 MHz**, **rt)** *δ* 50.7 (dd, *^2^J_P-P_* = 27, 112 Hz, CH_2_*P*(*i*Pr)_2_), −65.6 (td, ^2^*J_P-P_* = 27, 112 Hz, *P’*(CH_3_)_3_), −69.9 (br. s, Δ*w_½_* = 95 Hz, *P*(CH_3_)_3_). **^31^P{^1^H} NMR (THF-d_8_**, **121.5 MHz**, **rt)** *δ* 50.6 (dd, *^2^J_P-P_* = 28, 111 Hz, CH_2_*P*(*i*Pr)_2_), −66.4 (td, ^2^*J_P-P_* = 27, 111 Hz, *P’*(CH_3_)_3_), −70.0 (br. s, Δ*w_½_* = 100 Hz, *P*(CH_3_)_3_). **^13^C{^1^H} NMR (PhMe-d_8_**, **100.7 MHz**, **rt)** *δ* = 141.2 (app. t, *J_C-P_* = 5 Hz, 1-*C*_Ar_), 117.3 (s, 3-H*C*_Ar_), 107.5 (s, 2-H*C*_Ar_), 47.7 (app. td, app. t, *J_C-P_* = 19, 10 Hz, *C*H_2_), 30.3 (overlapping m, *C’*H(CH_3_)_2_ and P(*CH*_3_)_3_), 28.9 (app. t, *J_C-P_* = 11 Hz, *C*H(CH_3_)_2_), 27.9 (br. d, *J_C-P_* = 19 Hz, P’(*CH*_3_)_3_), 21.2 (s, CH(C*H*_3_)_2_), 19.8 (s, CH(C*H*_3_)_2_), 19.7 (s, C’H(CH_3_)(*C*’H_3_)), 18.8 (s, C’H(*C*H_3_)(C’H_3_)). **Anal. Calcd. for C_26_H_54_BN_2_P_4_Ir (2c):** C, 43.27; H, 7.54; N, 3.88. Found: C, 42.79; H, 7.27; N, 4.02. 

#### 4.2.6. [(d(CH_2_P(*i*Pr)_2_)abB)Ir(PMe_3_)] (**3c**)

In a Schlenk-flask, **5c** (50 mg, 68 μmol, 1 equiv.) was dissolved in THF (5 mL). Trimethylphosphine (34.7 µL, 26 mg, 0.342 mmol, 5 equiv.) was added, and the solution was stirred for 5 min at room temperature. The solvent was removed in vacuo. The colourless residue was dissolved in THF (5 mL), and a solution of KO*t*Bu (7.6 mg, 68 μmol, 1 equiv.) in THF (1 mL) was added. The resulting red–green solution was stirred for 2 h at room temperature. The solvent was removed in vacuo, and the residue was extracted with *n*-pentane (2 × 5 mL). The extract was filtered through a pad of celite and stored at −40 °C. After 24 h, dark red crystals with a greenish hue had separated. The supernatant solution was decanted, and the residue was washed with cold *n*-pentane (2 × 1 mL) and dried in vacuo (22 mg, 34 μmol, 50%).

**^1^H NMR (PhMe-d_8_**, **400.4 MHz**, **rt)** *δ* 7.08–7.03 (m, 2 H, 3-*H*C_Ar_), 6.99–6.93 (m, 2 H, 2-*H*C_Ar_), 3.63 (app. t, *J_H-P_* = 2.1, 2.1 Hz, 4 H, C*H_2_*), 2.04 (app. sept. t, *^3^J_H-H_* = 7.0, 7.0 Hz, *J_H-P_* = 2.2, 2.2 Hz, 4 H, C*H*(CH_3_)_2_), 1.42 (d, ^2^*J_H-P_* = 5.7 Hz, 9 H, PMe_3_), 1.05 (app. q, *^3^J_H-H_* = 7.0 Hz, *J_H-P_* = 7.9, 7.9 Hz, 12 H, CH(C*H*_3_)(C’H_3_)), 0.97 (app. q, *^3^J_H-H_* = 7.0 Hz, *J_H-P_* = 6.1, 6.1 Hz, 12 H, CH(CH_3_)(C’*H*_3_)). **^1^H NMR (THF-d_8_**, **300.1 MHz**, **rt)** *δ* 6.73–6.66 (m, 2 H, *H*C_Ar_), 6.66–6.58 (m, 2 H, *H*C_Ar_), 3.74 (app. t, *J_H-P_* = 2.0, 2.0 Hz, 4 H, C*H_2_*), 2.32 (app. sept. t, *^3^J_H-H_* = 7.0, 7.0 Hz, *J_H-P_* = 2.2, 2.2 Hz, 4 H, C*H*(CH_3_)_2_), 1.57 (d, ^2^*J_H-P_* = 5.7 Hz, 9 H, PMe_3_), 1.17 (app. q, *^3^J_H-H_* = 7.0 Hz, *J_H-P_* = 7.9, 7.9 Hz, 12 H, CH(C*H*_3_)(C’H_3_)), 1.11 (app. q, *^3^J_H-H_* = 7.0 Hz, *J_H-P_* = 6.1, 6.1 Hz, 12 H, CH(CH_3_)(C’*H*_3_)). **^13^C{^1^H} NMR (PhMe-d_8_**, **100.7 MHz**, **rt)** *δ* 140.1 (app. t, *J_C-P_* = 8 Hz, *C*_Ar_), 117.7 (s, 3-H*C*_Ar_), 108.6 (s, 2-H*C*_Ar_), 44.4 (app. td, *J_C-P_* = 21, 12 Hz, N*C*H_2_P), 28.6 (app. t, *J_C-P_* = 12 Hz, *C*H(CH_3_)_2_), 24.8 (app. dt, *J_C-P_* = 24, 2 Hz, PMe_3_), 20.2 (app. dt, *J_C-P_* = 4 Hz, CH(*CH*_3_)(C’H_3_)), 19.3 (br. s, CH(CH_3_)(*C*’*H*_3_)). **^11^B{^1^H} NMR (PhMe-d_8_**, **128.5 MHz**, **rt)** *δ* 57.5 (s, Δ*w_½_* = 430 Hz). **^11^B{^1^H} NMR (THF-d_8_**, **96.3 MHz**, **rt)** *δ* 55.3 (s, Δ*w_½_* = 380 Hz). **^31^P{^1^H} NMR (PhMe-d_8_**, **162.1 MHz**, **rt)** *δ* 81.5 (d, *J_P–P_* = 5 Hz, CH_2_P(*i*Pr)_2_), −18.6 (br s, P(CH_3_)_3_). **^31^P{^1^H} NMR (THF-d_8_**, **121.5 MHz**, **rt)** *δ* 80.0 (d, *J_P–P_* = 5 Hz, CH_2_*P*(*i*Pr)_2_), −20.1 (br s, P(CH_3_)_3_). **Anal. Calcd. for C_23_H_45_BIrN_2_P_3_ (3c)**: C, 42.79; H, 7.03; N, 4.34. Found: C, 43.22; H, 7.25; N, 4.59. **m.p.:** 204–206 °C.

#### 4.2.7. [(d(CH_2_P(*i*Pr)_2_)abB)IrCl(Bpin)] (**5c**)

In a Schlenk-flask, **1** (100 mg, 0.198 mmol, 1 equiv.) and [Ir(cod)Cl]_2_ (66.5 mg, 99 μmol, 1 equiv. Ir) were combined in *n*-pentane (50 mL). The yellow suspension was stirred at room temperature overnight before all volatiles were removed in vacuo. The bright yellow residue was recrystallised from *n*-pentane (20 mL) at −40 °C to give **5c** as bright yellow crystals (107 mg, 0.146 mmol, 74%). 

**^1^H NMR (THF-d_8_**, **400.4 MHz**, **rt)** *δ* 6.77–6.71 (m, 2 H, 2-*H*C_Ar_), 6.69–6.64 (m, 2 H, 3-*H*C_Ar_), 3.87 (app. dt, *^2^J_H-H_* = 11.6 Hz, *J_H-P_* = 2.0, 2.0 Hz, 2 H, C*H*H’), 3.77 (app. dt, *^2^J_H-H_* = 11.3 Hz, *J_H-P_* = 3.0, 3.0 Hz, 2 H, CH*H*’), 3.02 (m, *^3^J_H-H_* = 7.2, 7.1 Hz, *J_H-P_* = 3.4, 2.2 Hz, 2 H, C*H*(CH_3_)_2_), 2.99 (m, *^3^J_H-H_* = 7.6, 7.3 Hz, *J_H-P_* = 4.8, 5.8 Hz, 2 H, C’*H*(CH_3_)_2_), 1.47 (m, *^3^J_H-H_* = 7.6 Hz, *J_H-P_* = 7.9, 9.0 Hz, 6 H, C’H(C*H*_3_)(C’H_3_)), 1.45 (app. q, *^3^J_H-H_* = 7.2 Hz, *J_H-P_* = 7.4, 7.4 Hz, 6 H, CH(C*H*_3_)(C’H_3_)), 1.39 (app. q, *^3^J_H-H_* = 7.3 Hz, *J_H-P_* = 6.5, 6.5 Hz, 6 H, C’H(CH_3_)(C’*H*_3_)), 1.14 (app. q, *^3^J_H-H_* = 7.1 Hz, *J_H-P_* = 7.0, 7.0 Hz, 6 H, CH(CH_3_)(C’*H*_3_)), 0.81 (s, 12 H, OC(C*H*_3_)_2_). **^13^C{^1^H} NMR (THF-d_8_**, **100.7 MHz**, **rt)** *δ* 140.8 (app. t, *J_C-P_* = 7 Hz, *C*_Ar_), 118.3 (s, 3-H*C*_Ar_), 108.2 (s, 2-H*C*_Ar_), 83.2 (s, OC*(*CH_3_)_2_), 45.7 (app. t, *J_C-P_* = 22 Hz, N*C*H_2_P), 29.8 (app. t, *J_C-P_* = 12 Hz, *C*H(CH_3_)_2_), 28.9 (app. t, *J_C-P_* = 12 Hz, *C’*H(CH_3_)_2_), 24.8 (s, OC(*C*H_3_)_2_), 20.4 (app. t, *J_C-P_* = 3 Hz, C’H(*C*H_3_)(C’H_3_)), 19.7 (s, CH(CH_3_)(*C*’H_3_)), 18.7(s, CH(*C*H_3_)(C’H_3_)), 18.2 (s, C’H(CH_3_)(*C*’H_3_)). **^11^B{^1^H} NMR (THF-d_8_**, **128.5 MHz**, **rt)** *δ* 39.7 (s, Δ*w_½_* = 340 Hz), 19.9 (s, Δ*w_½_* = 330 Hz). **^31^P{^1^H} NMR (THF-d_8_**, **162.1 MHz**, **rt)** *δ* 66.4 (s). **Anal. Calcd. for C_26_H_48_B_2_N_2_O_2_P_2_IrCl (5c):** C, 42.67; H, 6.61; N, 3.83. Found: C, 42.48; H, 6.41; N, 4.06. **m.p.:** 232–234 °C.

#### 4.2.8. [(d(CH_2_P(*i*Pr)_2_)abB)IrCl(Bpin)(PMe_3_)] (**6c**)

In a nitrogen-filled glovebox, **5c** (15 mg, 20 μmol, 1 equiv.) was dissolved in *n*-pentane (5 mL), and trimethylphosphine (10.4 µL, 7.8 mg, 0.102 mmol, 5 equiv.) was added. The solvent was removed after 5 min at room temperature to give **6c** as a colourless solid. Single crystalline **6c** was obtained from the above mixture upon crystallisation at −40 °C (6 mg, 7 μmol, 37%).

**^1^H NMR (THF-d_8_**, **400.4 MHz**, **rt)** *δ* 6.74–6.69 (m, 2 H, 2-*H*C_Ar_), 6.67–6.62 (m, 2 H, 3-*H*C_Ar_), 3.88 (app. dt, *^2^J_H-H_* = 11.0 Hz, *J_H-P_* = 2.2, 2.2 Hz, 2 H, C*H*H’), 3.64 (app. dt, *^2^J_H-H_* = 11.0 Hz, *J_H-P_* = 2.2, 2.2Hz, 2 H, CH*H*’), 3.06 (m, *^3^J_H-H_* = 7.1, 7.1 Hz, *J_H-P_* = 3.5, 3.5 Hz, 2 H, C*H*(CH_3_)_2_), 2.61 (m, *^3^J_H-H_* = 7.2, 7.2 Hz, *J_H-P_* = 3.7, 3.7 Hz, 2 H, C’*H*(CH_3_)_2_), 1.68 (d, ^2^*J_H-P_* = 7.2 Hz, 9 H, PMe_3_), 1.42 (app. q, *^3^J_H-H_* = 7.1 Hz, *J_H-P_* = 7.0, 7.0 Hz, 6 H, CH(C*H*_3_)(C’H_3_)), 1.41 (app. q, *^3^J_H-H_* = 7.1 Hz, *J_H-P_* = 7.0, 7.0 Hz, 6 H, CH(CH_3_)(C’*H*_3_)), 1.38 (app. q, *^3^J_H-H_* = 7.2 Hz, *J_H-P_* = 7.0, 7.0 Hz, 6 H, C’H(C*H*_3_)(C’H_3_)), 1.31 (app. q, *^3^J_H-H_* = 7.2 Hz, *J_H-P_* = 7.1, 7.1 Hz, 6 H, C’H(CH_3_)(C’*H*_3_)), 0.66 (s, 12 H, OC(C*H*_3_)_2_). **^13^C{^1^H} NMR (THF-d_8_**, **100.7 MHz**, **rt)** *δ* 142.4 (app. td, *J_C-P_* = 8, 2 Hz, *C*_Ar_), 117.9 (s, 3-H*C*_Ar_), 108.5 (s, 2-H*C*_Ar_), 81.7 (s, OC*(*CH_3_)_2_), 46.3 (app. td, *J_C-P_* = 20, 7 Hz, N*C*H_2_P), 31.1 (app. t, *J_C-P_* = 14 Hz, *C*H(CH_3_)_2_), 27.6 (app. td, *J_C-P_* = 11, 2 Hz, *C’*H(CH_3_)_2_), 25.7 (s, OC(*C*H_3_)_2_), 20.5 (d, ^2^*J_C-P_* = 24 Hz, PMe_3_), 21.1 (s, C’H(CH_3_)(*C*’H_3_)), 20.0 (s, C’H(*C*H_3_)(C’H_3_)), 19.6 (s, CH(*CH*_3_)(C’H_3_)), 19.0 (s, CH(CH_3_)(*C*’*H*_3_)). **^11^B{^1^H} NMR (THF-d_8_**, **128.5 MHz**, **rt)** *δ* 48.8 (s, Δ*w_½_* = 460 Hz), 26.6 (s, Δ*w_½_* = 450 Hz). **^31^P{^1^H} NMR (THF-d_8_**, **162.1 MHz**, **rt)** *δ* 41.4 (d, *J_P–P_* = 12 Hz, CH_2_*P*(*i*Pr)_2_), −51.9 (br. s, Δ*w_½_* = 70 Hz, PMe_3_). **Anal. Calcd. for C_29_H_57_B_2_N_2_O_2_P_3_IrCl (6c):** C, 43.11; H, 7.11; N, 3.47. Found: C, 42.83; H, 7.03; N, 3.58. **m.p.:** 182–184 °C (decomp).

## Data Availability

The data presented in this study are available in the article or Supplementary Materials.

## Supplementary Materials

The following supporting information can be downloaded at: https://www.mdpi.com/article/10.3390/molecules28176191/s1, additional spectroscopic and experimental details, crystallographic and computational details [44,45,46,47,48,49]. Crystallographic data (including structure factors) for the structures reported in this paper have been deposited with the Cambridge Crystallographic Data Centre. CCDC 2269774–2269781 contains the supplementary crystallographic data for this paper. These data can be obtained free of charge from The Cambridge Crystallographic Data Centre via www.ccdc.cam.ac.uk/structures (accessed on 28 June 2023).

## Sample Availability

Not available.
