# Supplementary material for: Synthesis, Reactivity and Coordination Chemistry of Group 9 PBP Boryl Pincer Complexes: [(PBP)M(PMe3)n] (M = Co, Rh, Ir; n = 1, 2)
Source: Molecules. 2023 Aug 22;28(17):6191. doi: 10.3390/molecules28176191 (PMC10488511; doi:10.3390/molecules28176191)
Supplement: Supplementary file 1 [file molecules-28-06191-s001.zip › molecules-2504652-SI.pdf]

<u>Supplementary Information</u>

Synthesis, Reactivity and Coordination Chemistry of Group 9 PBP Boryl Pincer complexes: [(PBP)M(PMe$_3$)$_n$] (M = Co, Rh, Ir; n = 1, 2)

Philipp M. Rutz [1], Jörg Grunenberg [2] and Christian Kleeberg [1,*]

[1] Institute of Inorganic and Analytical Chemistry, Technische Universität Braunschweig, Hagenring 30, 38106 Braunschweig, Germany
[2] Institute of Organic Chemistry, Technische Universität Braunschweig, Hagenring 30, 38106 Braunschweig, Germany
* Correspondence: ch.kleeberg@tu-braunschweig.de; Tel.: +49-531-391-5392

Contents

1. Additional Experimental and Spectroscopic Data
   a. [(d(CH$_2$P(*i*Pr)$_2$)abB)Co(PMe$_3$)$_2$] (**2a**) S2
   b. [(d(CH$_2$P(*i*Pr)$_2$)abB)Co(N$_2$)(PMe$_3$)] (**4a**) (*in situ* NMR data) S6
   c. [(d(CH$_2$P(*i*Pr)$_2$)abB)Rh–PMe$_3$] (**3b**) S8
   Reaction of **1** with [(Me$_3$P)$_3$Rh–Cl] and KO*t*Bu S12
   d. [(d(CH$_2$P(*i*Pr)$_2$)abB)Rh(PMe$_3$)$_2$] (**2b**) S14
   Reaction of **3b** with Me$_3$P to **2b** – NMR experiments S17
   Reaction of **3b** with Me$_3$P to **2b** – UV-Vis experiments S20
   e. [(d(CH$_2$P(*i*Pr)$_2$)abB)IrCl(Bpin)] (**5c**) S22
   f. [(d(CH$_2$P(*i*Pr)$_2$)abB)IrCl(Bpin)(PMe$_3$)] (**6c**) S24
   g. [(d(CH$_2$P(*i*Pr)$_2$)abB)Ir(PMe$_3$)$_2$] (**2c**) S26
   Reaction of **3c** with Me$_3$P to **2c** – UV-Vis experiments S29
   h. [(d(CH$_2$P(*i*Pr)$_2$)abB)Ir–PMe$_3$] (**3c**) S31
   Reaction of **6c** with KO*t*Bu S33

2. Crystallographic Data
   a. Crystallographic Data Collection Parameters S36
   b. Overlay of **2a** with mirror image of **2a** S39
   c. Overlay of **2a**, **2b** and **2c** S40
   d. Overlay of **3b** and **3c** S41
   e. Overlay of the two independent molecules in **4a** S42

3. Computational Data
   a. [(d(CH$_2$P(*i*Pr)$_2$)abB)M(PMe$_3$)$_n$] (M = Co, Rh, Ir; n = 1,2) S43

4. References S46

1. Additional Experimental and Spectroscopic Data

a. [(d(CH_2P(iPr)$_2$)abB)Co(PMe_3)$_2$] (2a)

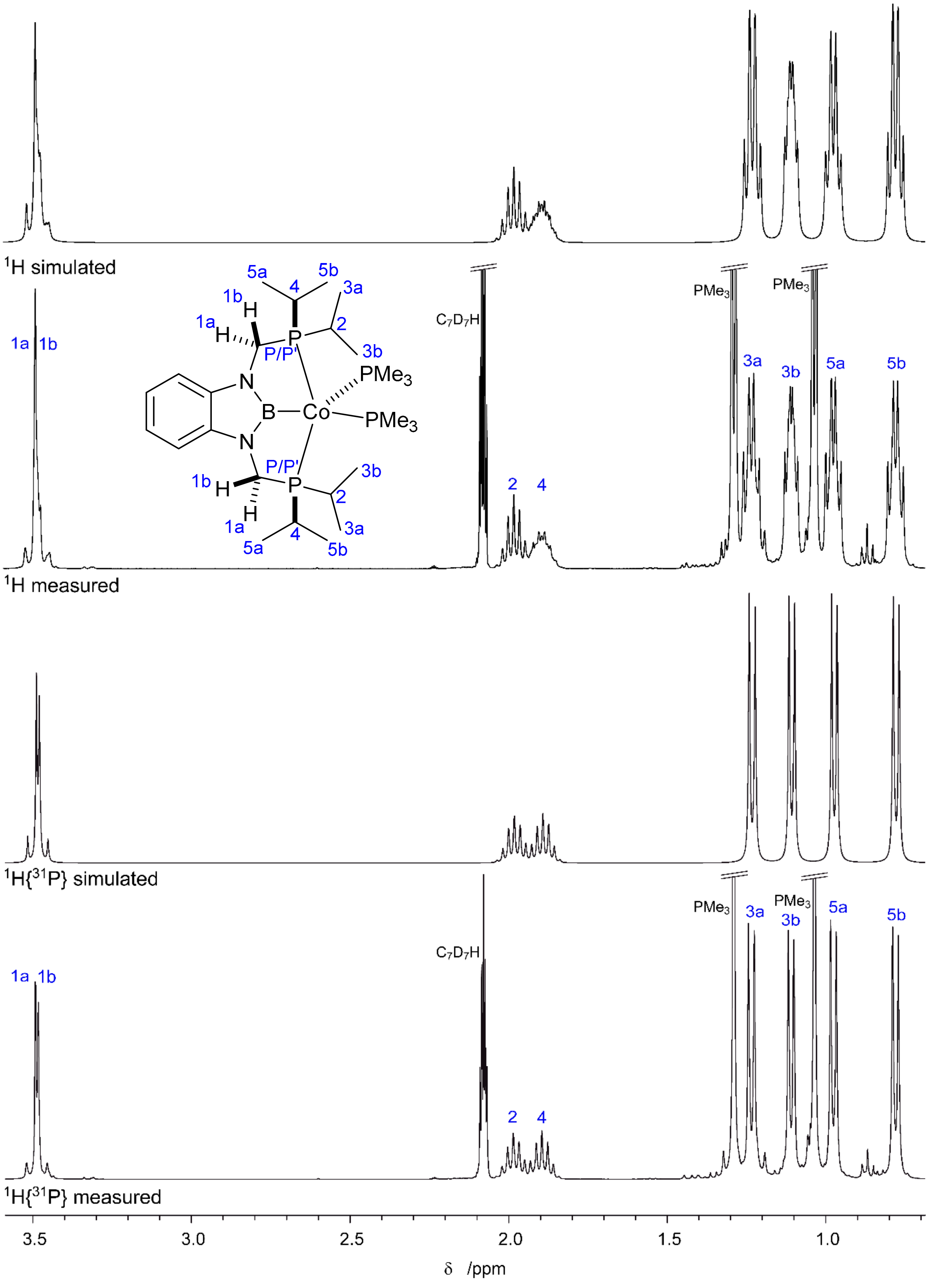

Figure S1: Section of the ^{1}H{^{31}P} and ^{1}H NMR spectrum of **2a** and simulation of selected signals (400.4 MHz, PhMe-d$_8$, rt).

Table S1: Coupling constants (Hz) used in the simulation of the ^{1}H{^{31}P} and ^{1}H NMR spectrum of **2a** (Figure S1).

Signal (δ/ppm)	1a (3.50)	1b (3.47)	2 (1.98)	4 (1.89)	3a (1.23)	3b (1.11)	5a (0.98)	5b (0.78)	P/P' (83.0)
1a (3.50)	X	11.02							0
1b (3.47)	11.02	X							4
2 (1.98)			X		7.56	6.97			0.0
4 (1.89)				X			6.97	7.36	2.5/4.0
3a (1.23)			7.56		X				6.8/6.0
3b (1.11)			6.97			X			5.7/3.6
5a (0.98)				6.97			X		6.9/5.6
5b (0.78)				7.36				X	6.5/5.7
P/P' (83.0)	0	4	0.0	2.5/4.0	6.8/6.0	5.7/3.6	6.9/5.6	6.5/5.7	X

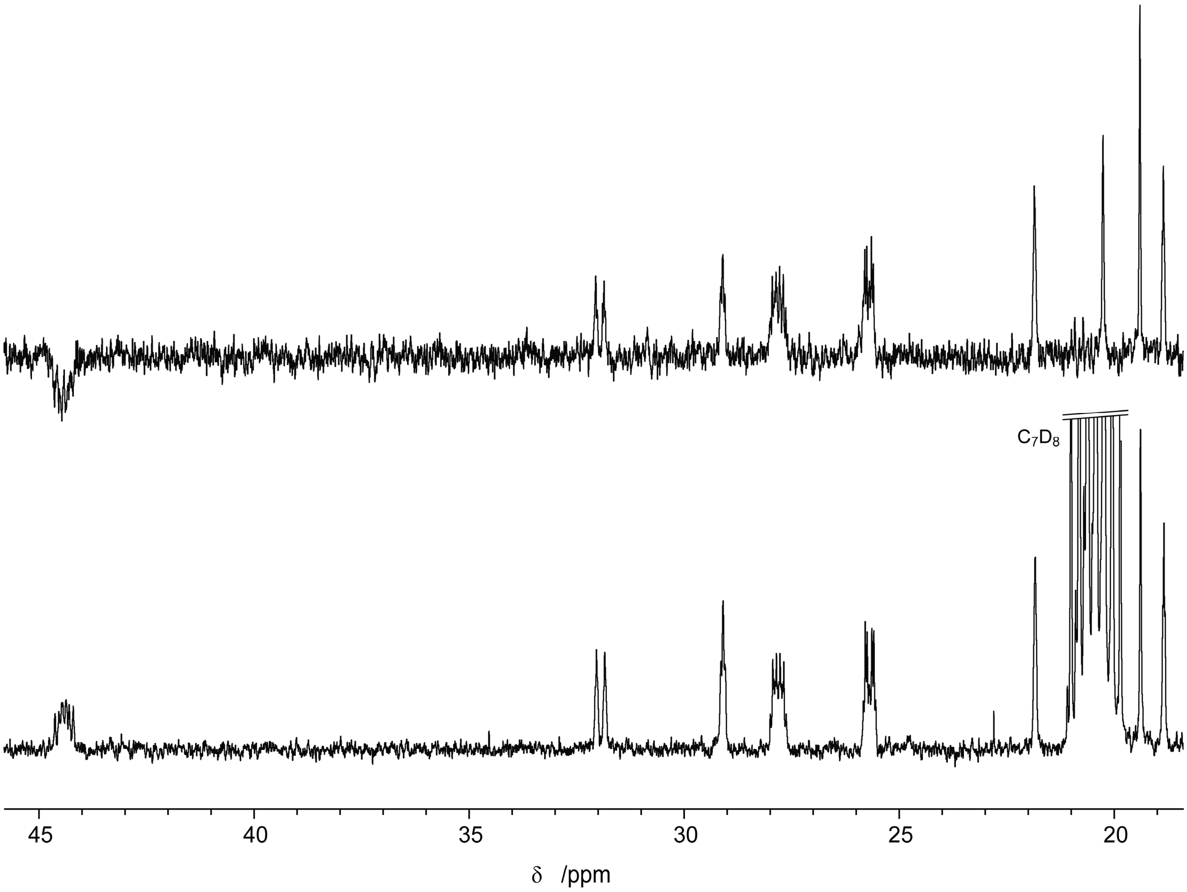

Figure S2: Section of the ^{13}C{^{1}H} (bottom) and ^{13}C{^{1}H}-DEPT NMR (top) spectrum of **2a** (100.7 MHz, PhMe-d_8, rt).

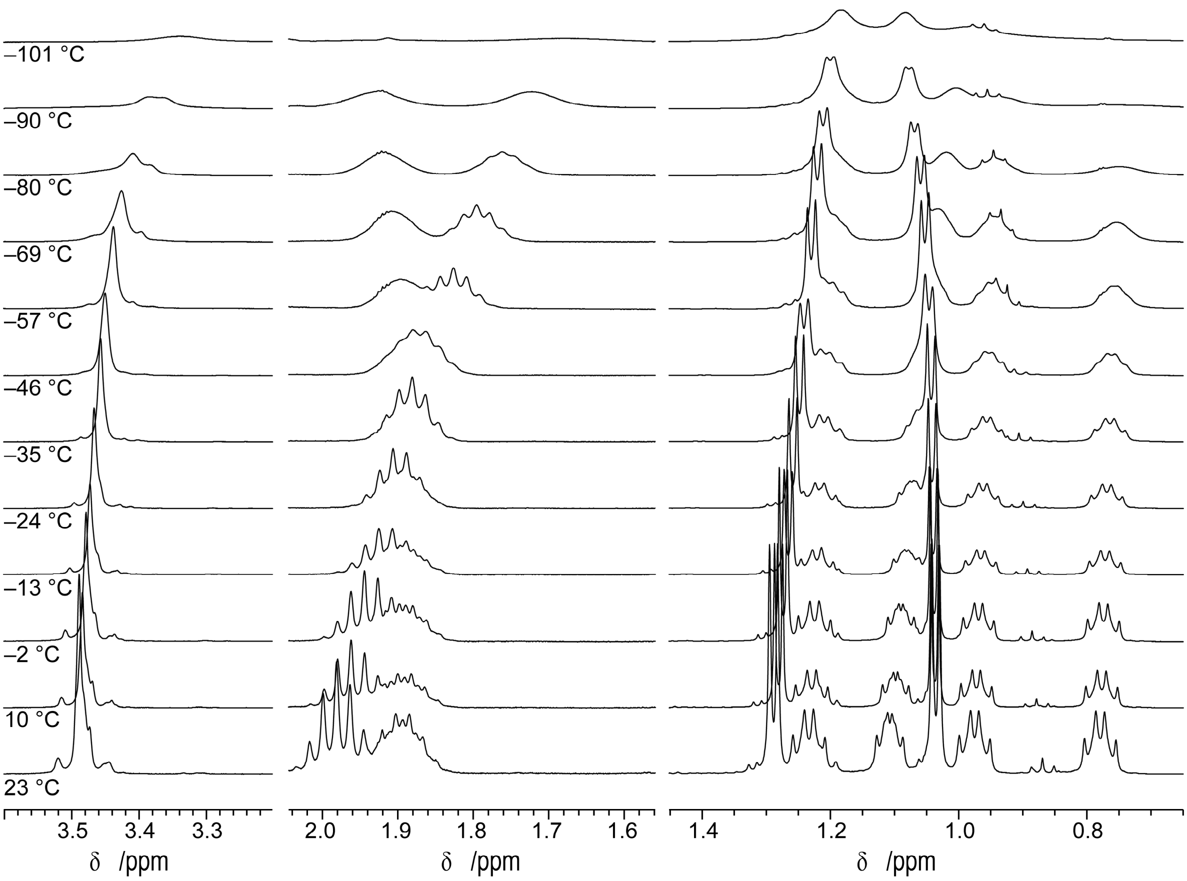

Figure S3: Section of the VT-^{1}H spectra of **2a** (400.4 MHz, PhMe-d_8).

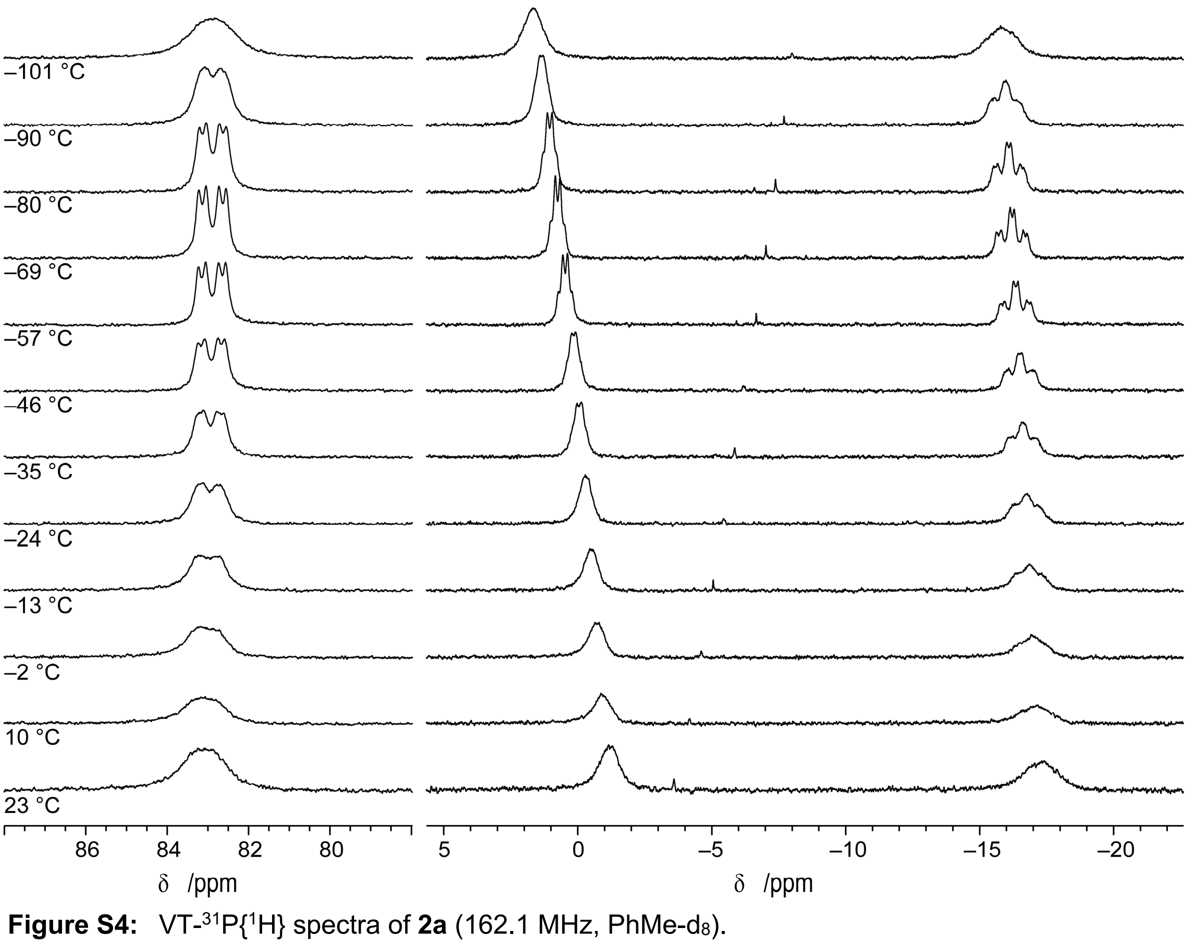

Figure S4: VT-$^{31}P\{^{1}H\}$ spectra of **2a** (162.1 MHz, PhMe-d_8).

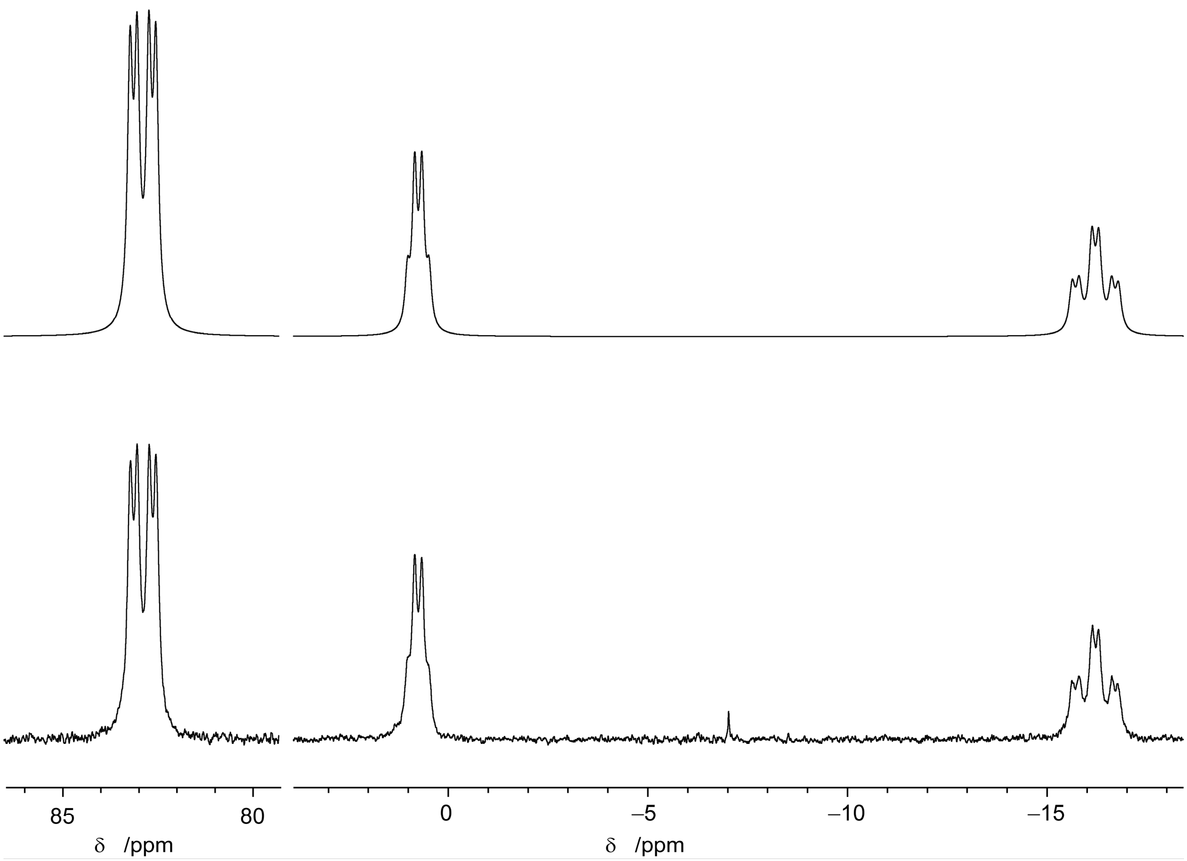

Figure S5: Measured $^{31}P\{^{1}H\}$ spectrum of **2a** at –69 °C (bottom) and simulated spectrum (top) (162.1 MHz, PhMe-d_8).

b. [(d(CH$_2$P(iPr)$_2$)abB)Co(N$_2$)(PMe$_3$)] (4a) (*in situ* NMR data)

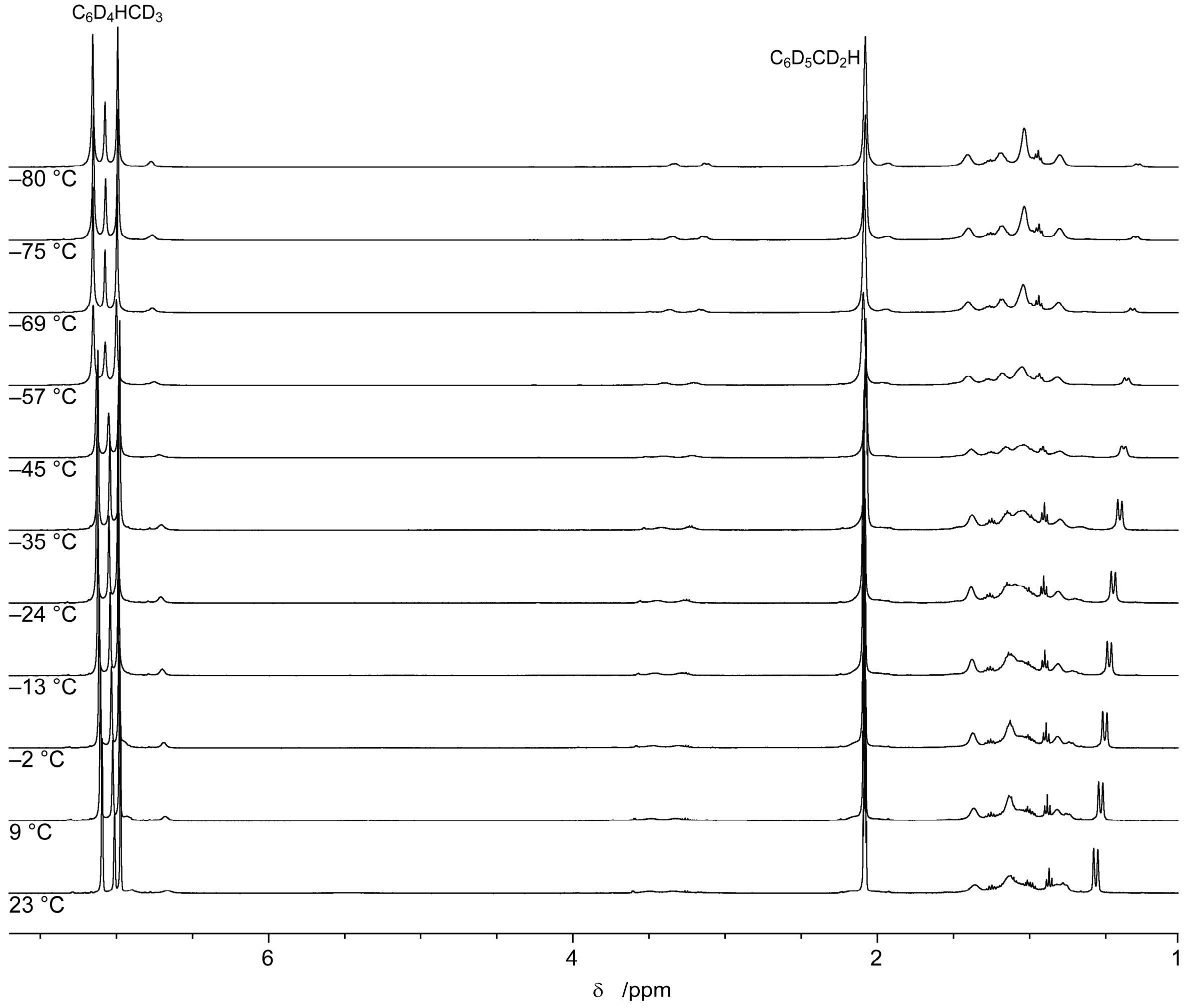

Figure S6: VT-^{1}H spectra of **2a** + B(C$_6$F$_5$)$_3$ (400.4 MHz, PhMe-d$_8$).

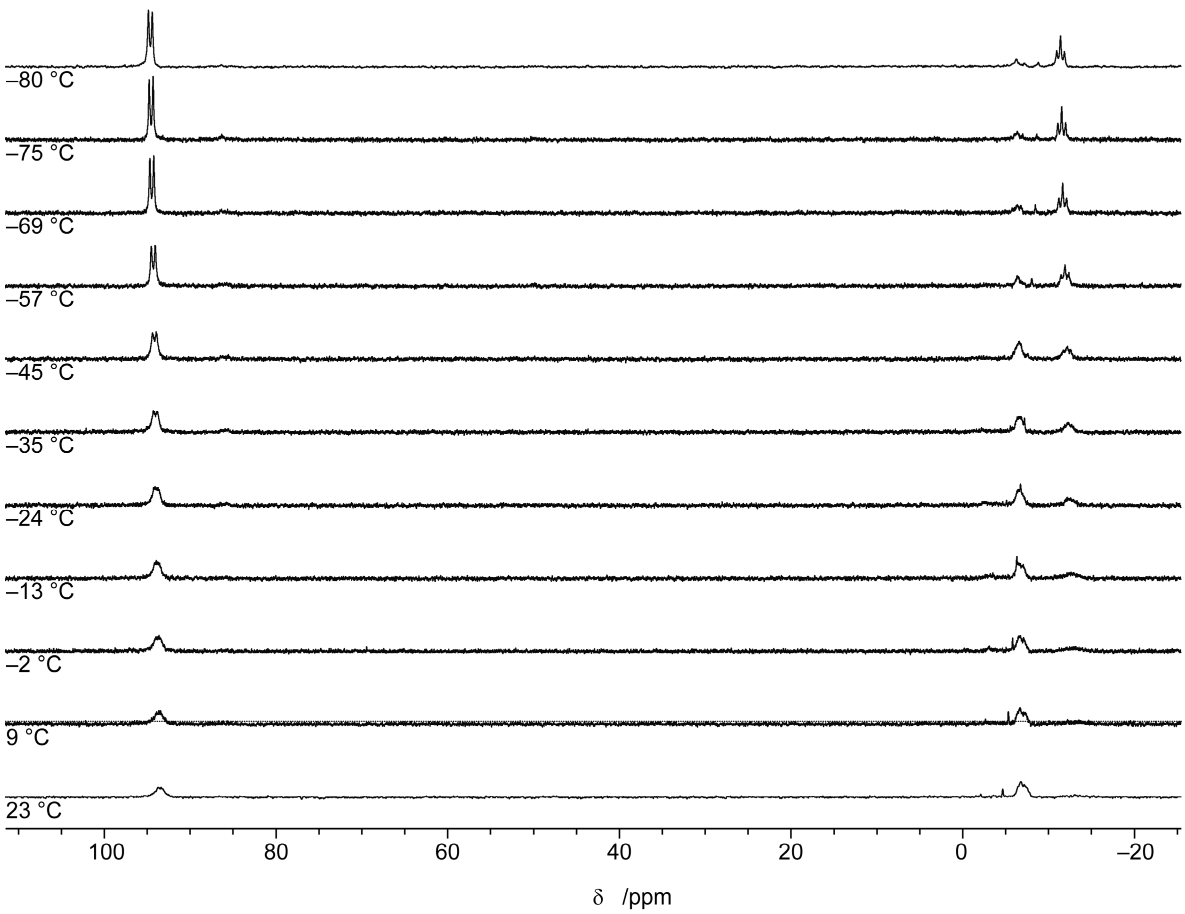

Figure S7: VT-$^{31}P\{^1H\}$ spectra of **2a** + $B(C_6F_5)_3$ (162.1 MHz, PhMe-d_8).

c. [(d(CH2P(*i*Pr)2)abB)Rh–PMe3] (3b)

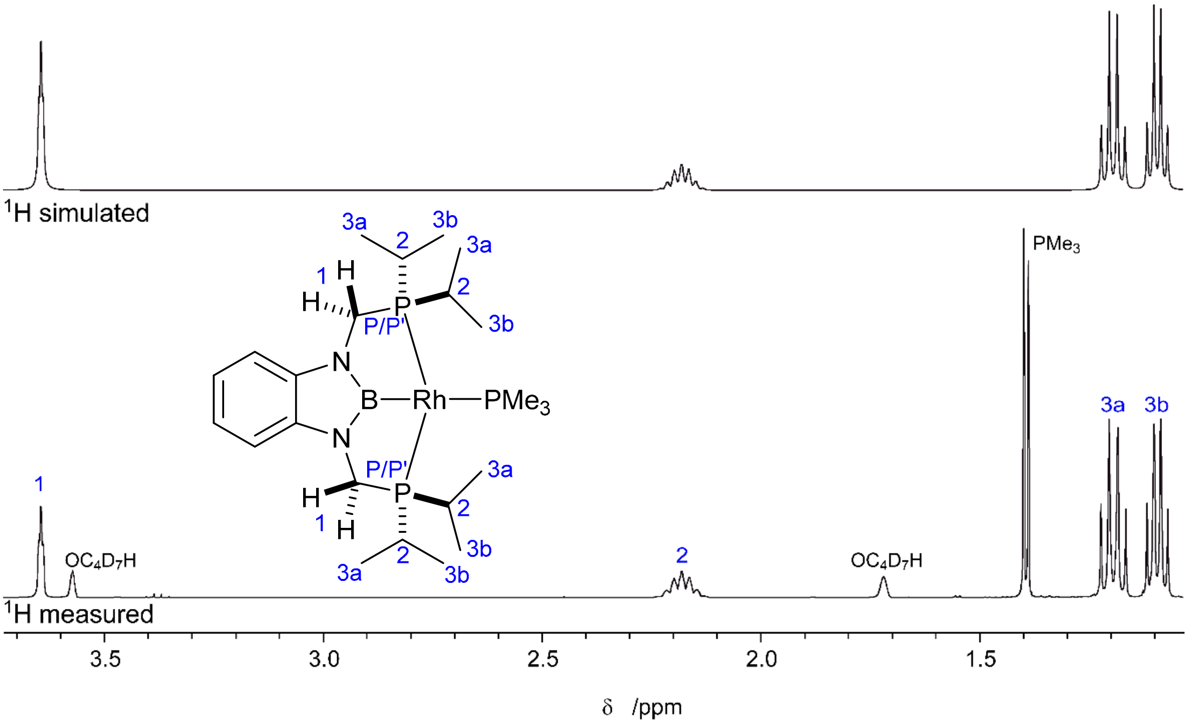

Figure S8: Section of the ^1H spectrum of **3b** and simulation of selected signals (400.4 MHz, THF-d_8, rt).

Table S2: Coupling constants (Hz) used in the simulation of the ^1H NMR spectrum of **3b** (Figure S6).

Signal (δ/ppm)	1 (3.64)	2 (2.18)	3a (1.19)	3b (1.09)	P/P'/Rh
1 (3.64)	X				2/2
2 (2.18)		X	7.0	6.0	1.5/1.5
3a (1.19)		7.0	X		7.4/7.4
3b (1.09)		6.0		X	6.3/6.3
P/P'/Rh	2/2	1.5/1.5	7.4/7.4	6.3/6.3	X

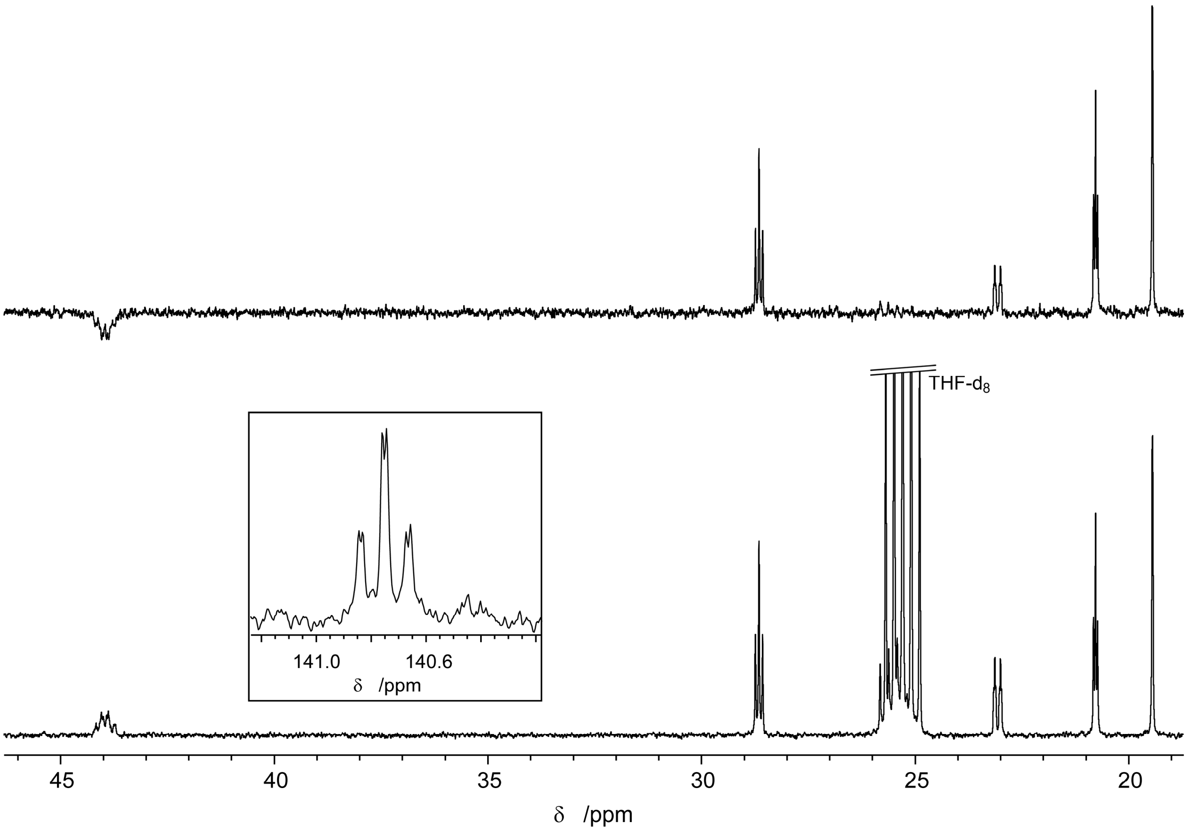

Figure S9: Section of the $^{13}C\{^{1}H\}$ (bottom) and $^{13}C\{^{1}H\}$-DEPT NMR (top) spectrum of **3b** (100.7 MHz, THF-d_8, rt).

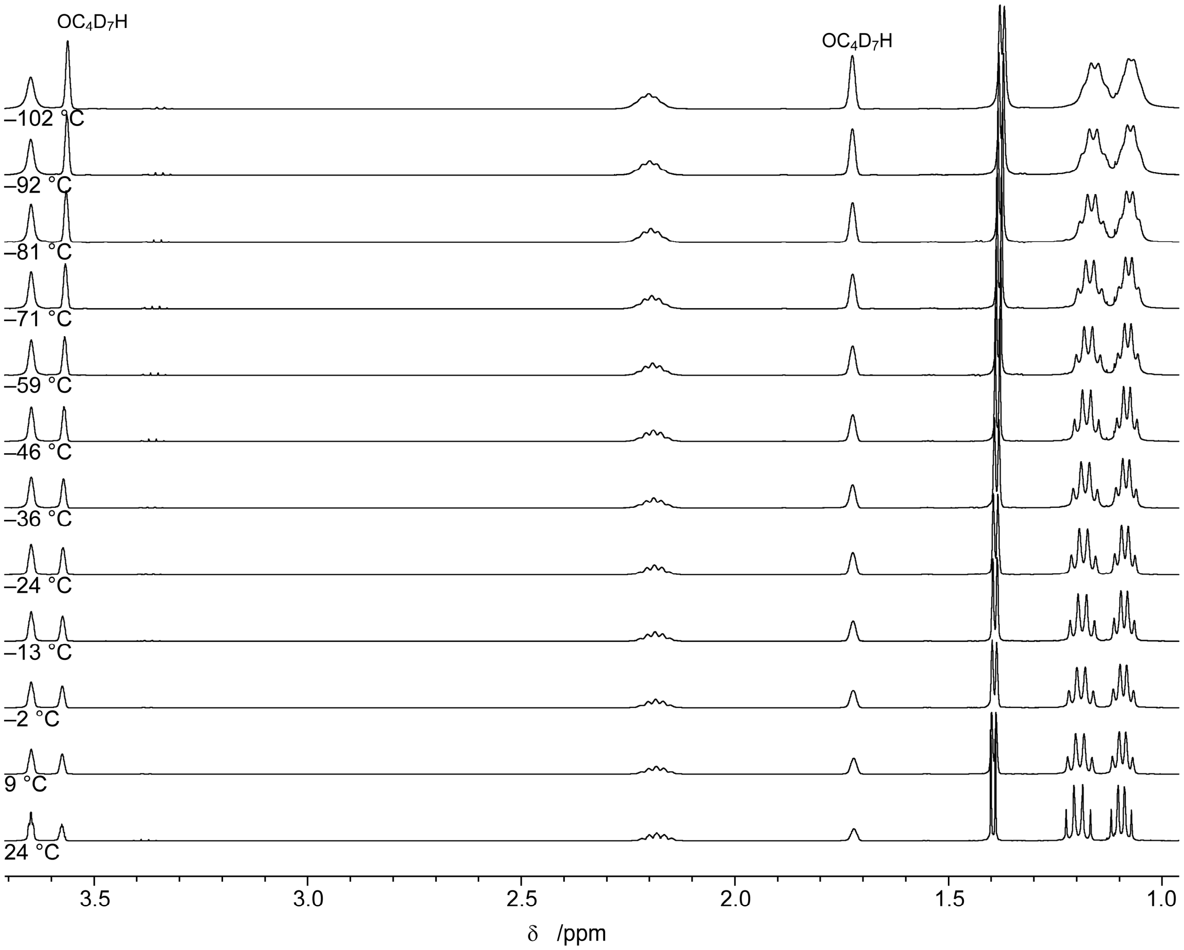

Figure S10: Section of the VT-^{1}H spectra of **3b** (400.4 MHz, THF-d_8).

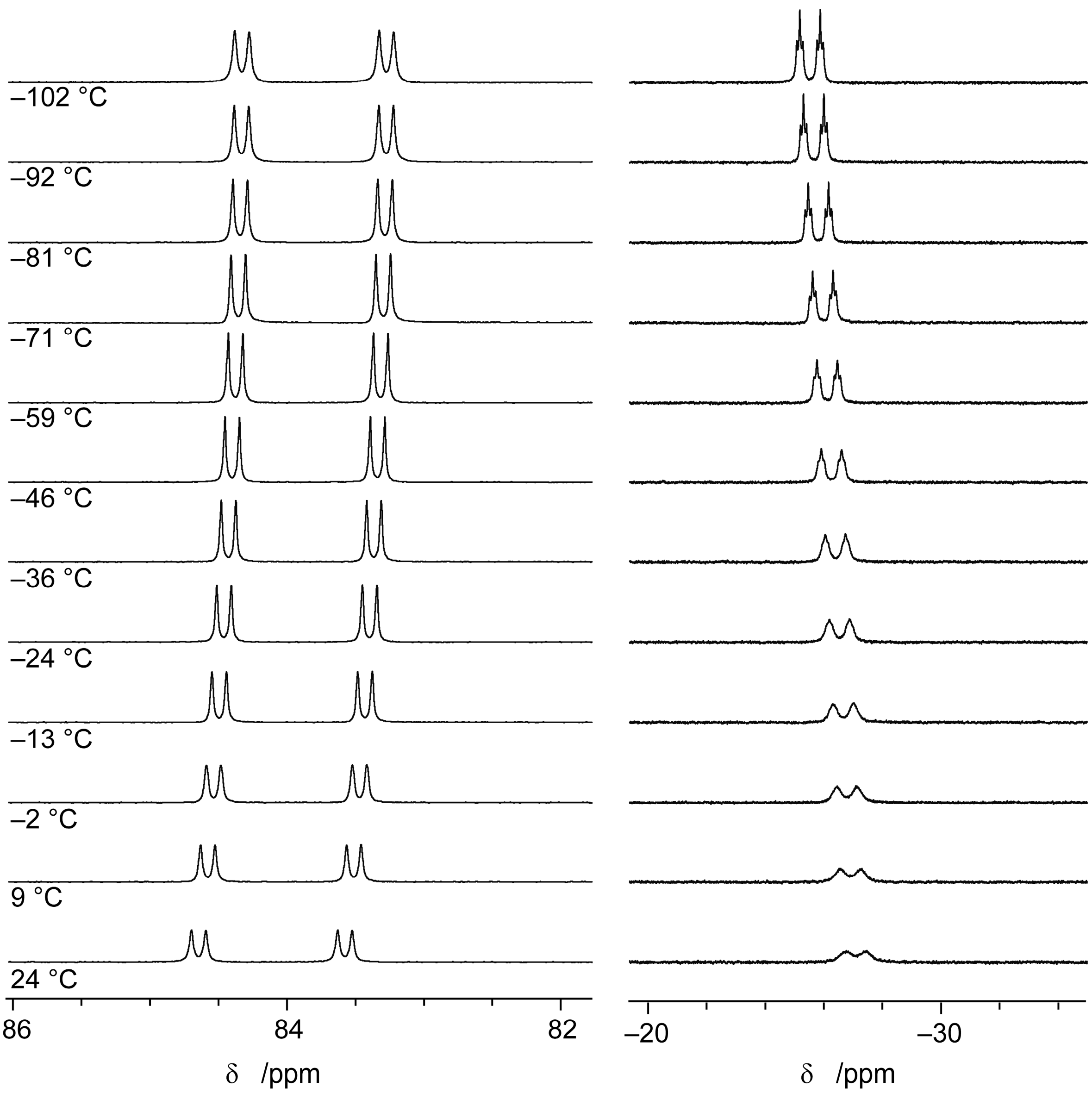

Figure S11: VT-$^{31}P\{^{1}H\}$ spectra of **3b** (162.1 MHz, THF-d_8).

Reaction of **1** with [(Me$_3$P)$_3$Rh–Cl] and KO*t*Bu

In a nitrogen filled glovebox, [(Me$_3$P)$_3$Rh–Cl] (12.1 mg, 33 µmol, 1 equiv.) and **1** (20.2 mg, 40 µmol, 1.2 equiv.) were dissolved in C_6D_6 (0.7 mL) and of NMR spectra were recorded after 20 min, 1.5 h, 3.5 h and 20 h. KO*t*Bu (3.7 mg, 33 µmol, 1 equiv) and THF-d$_8$ (0.1 mL) were added to the solution and a series of NMR spectra were recorded after 30 min.

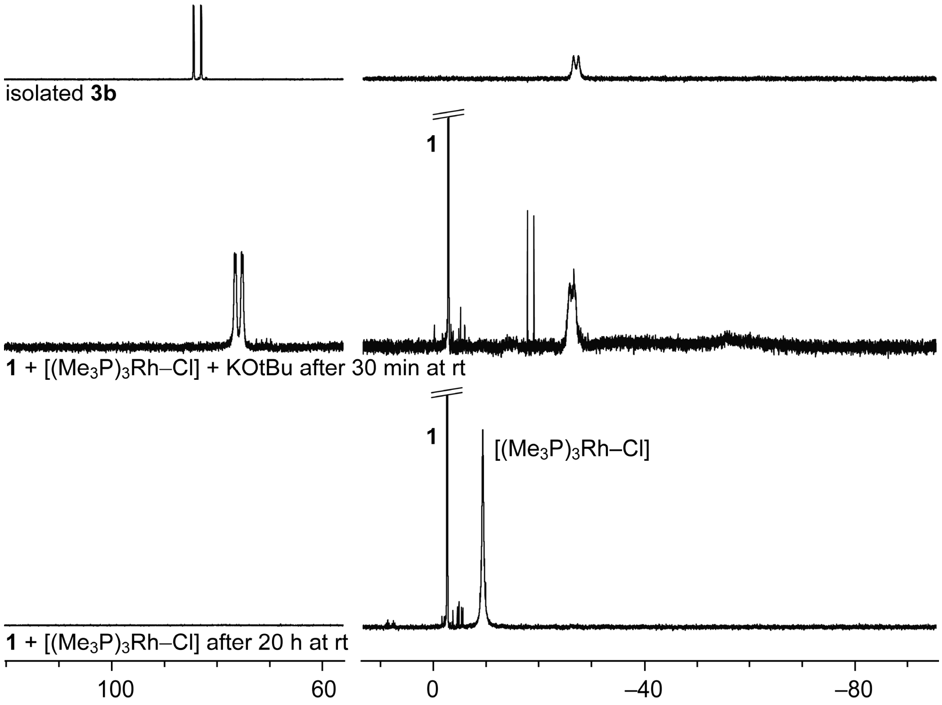

Figure S12: *In situ* $^{31}P\{^1H\}$ spectra of the reaction of **1** with [(Me$_3$P)$_3$Rh–Cl] (121.6 MHz, C_6D_6, rt).

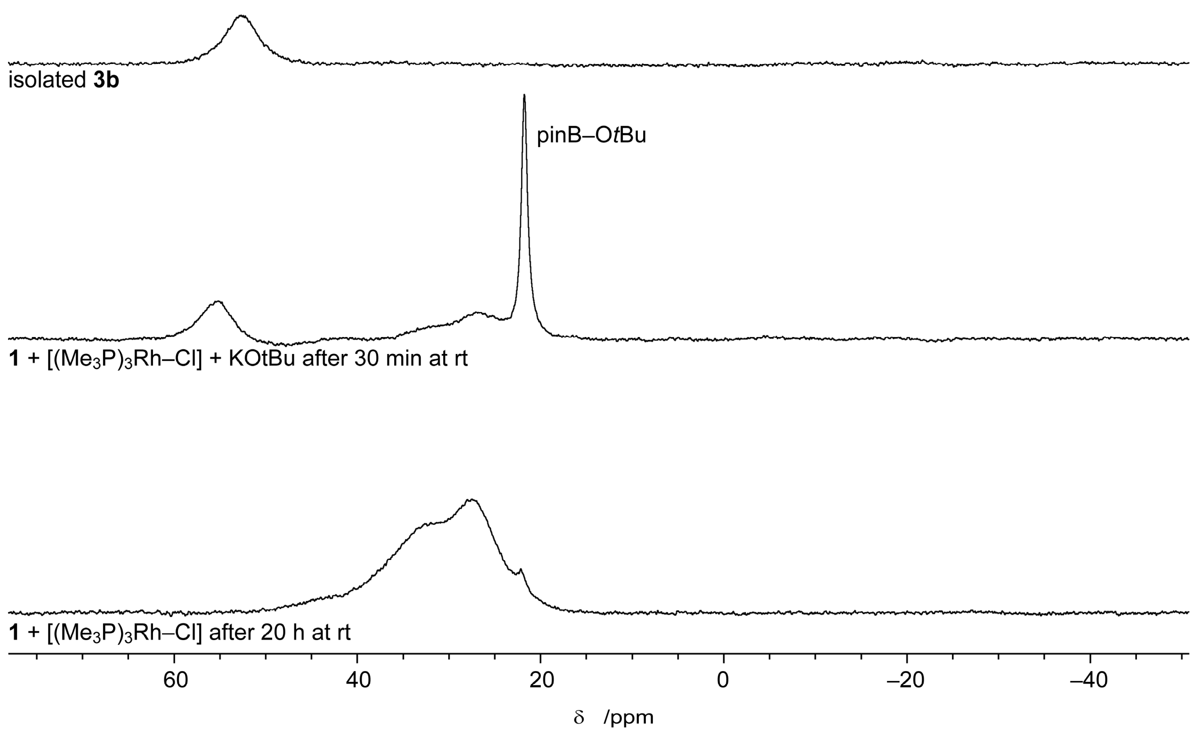

Figure S13: *In situ* $^{11}B\{^{1}H\}$ spectra of the reaction of excess **1** with [($Me_3P)_3Rh$–Cl] (96.3 MHz, C_6D_6, rt).

d. [(d(CH2P(*i*Pr)2)abB)Rh(PMe3)2] (2b)

The NMR spectra of **2b** (Figures S11 and S12) were recorded from a solution of **3b** (15 mg, 28 µmol) in THF-d8 (0.7 mL) after addition of PMe3 (3.7 µL, 2.7 mg, 0.037 mmol, 1.3 equiv.).

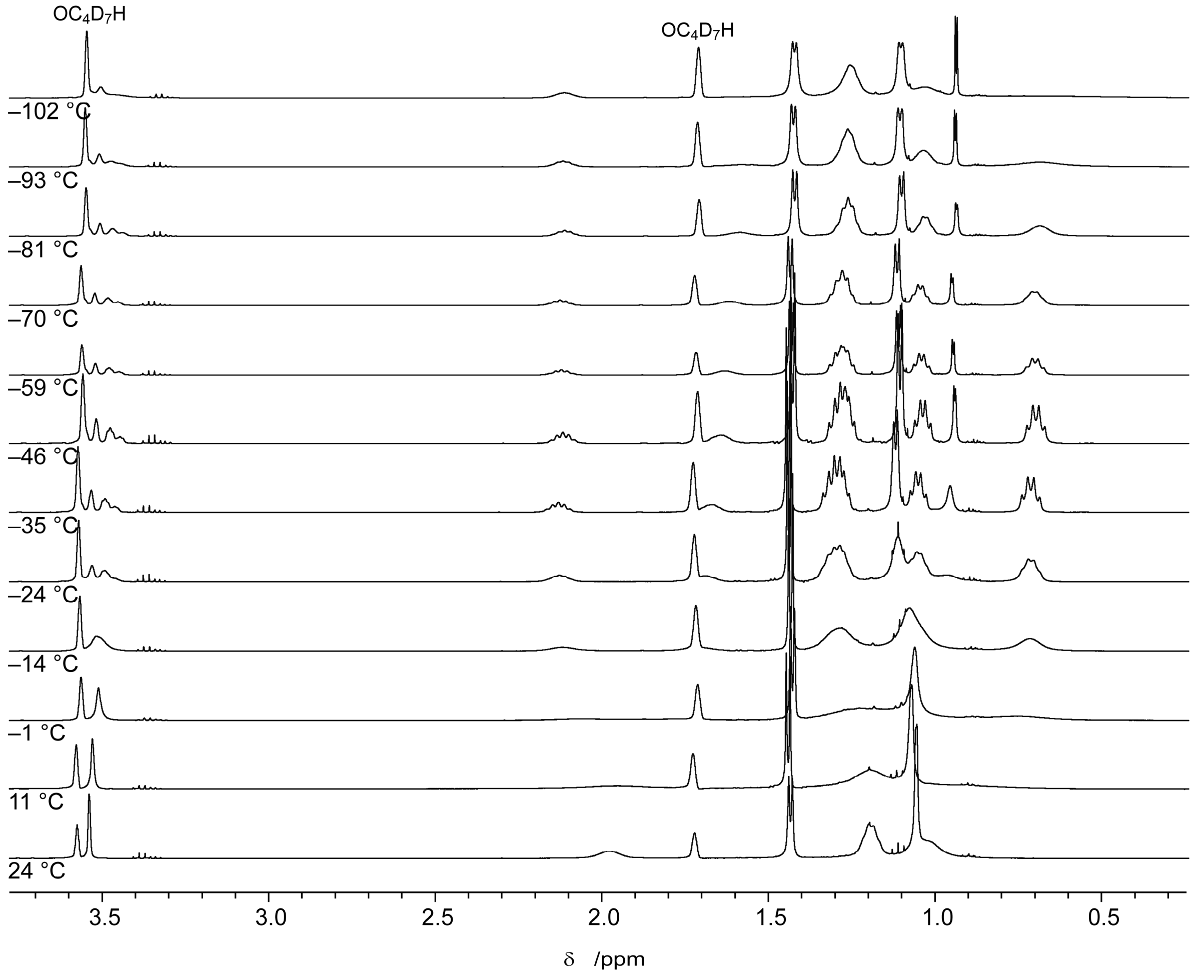

Figure S14: Section of the VT-^{1}H spectra of **2b** (400.4 MHz, THF-d8).

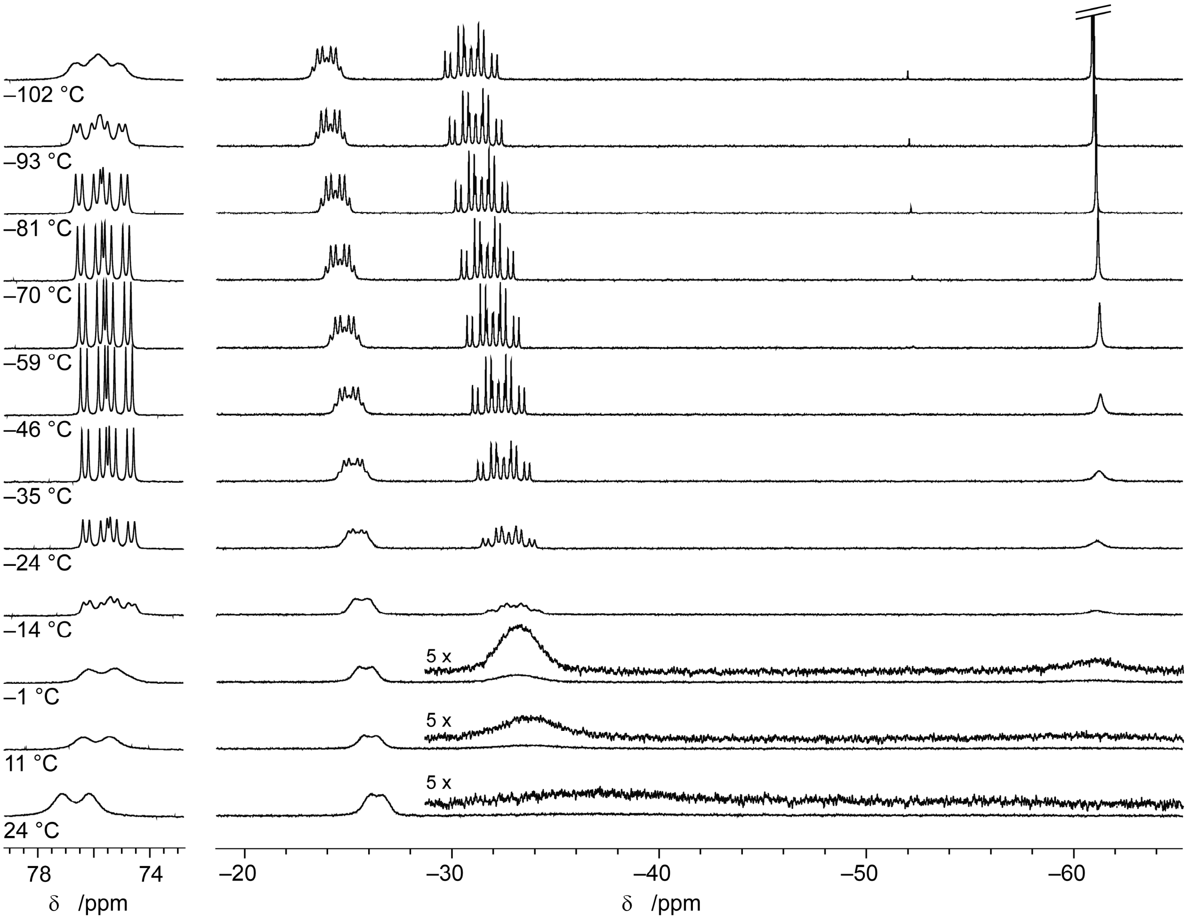

Figure S15: Sections of the VT-$^{31}P\{^1H\}$ spectra of **2b** (162.1 MHz, THF-d8).

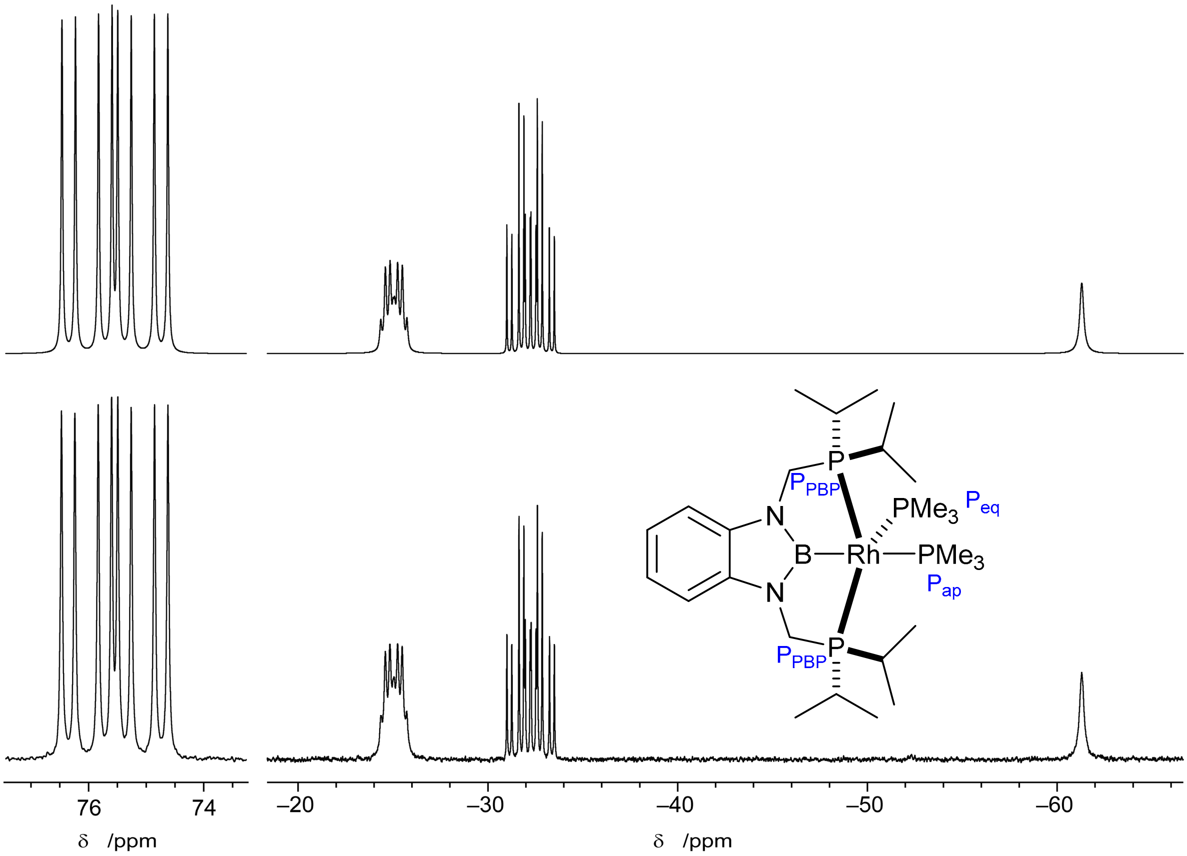

Figure S16: Measured $^{31}P\{^1H\}$ spectrum of **2b** at –46 °C (bottom) and simulated spectrum (top) (162.1 MHz, THF-d$_8$).

Table S3: Coupling constants (Hz) used in the simulation of the $^{31}P\{^1H\}$ NMR spectrum of **2b** (Figure S13).

Signal (δ/ppm)	P_{PBP} (75.5)	P_{ap} (–25.1)	P_{eq} (–32.3)	Rh
P_{PBP} (75.5)	X	38	103	157
P_{ap} (–25.1)	38	X	43	105
P_{eq} (–32.3)	103	43	X	157
Rh	157	105	157	X

Reaction of **3b** with Me_3P to **2b** – NMR experiments

In a nitrogen filled glovebox [(d(CH_2P(*i*Pr)$_2$)abB)Rh(PMe_3)] (**3b**) (10 mg, 18 µmol, 1 equiv.) was dissolved in C_6D_6 (ca. 0.65 mL) and a series of NMR experiments were performed. A solution of PMe_3 (92.7 µL, 69.3 mg, 0.91 mmol, 50 equiv.) in C_6D_6 (500 µL total volume of solution) was prepared. 5 µL of the PMe_3 solution (i.e. 0.5 equiv. PMe_3) was added to the solution of **3b** and the NMR experiments were repeated. Subsequently additional 5, 10, 30 and 50 µL of the PMe_3 solution (10, 20, 50 and 100 µL in total respectively, i.e. 1, 2, 5 and 10 equiv. PMe_3) were added and the NMR experiments were repeated after each step. At last, PMe_3 (73.2 µL, 54.8 mg, 0.72 mmol, 40 equiv.; i.e. 50 equiv. in total) was added to the reaction solution and the NMR experiments were repeated once more.

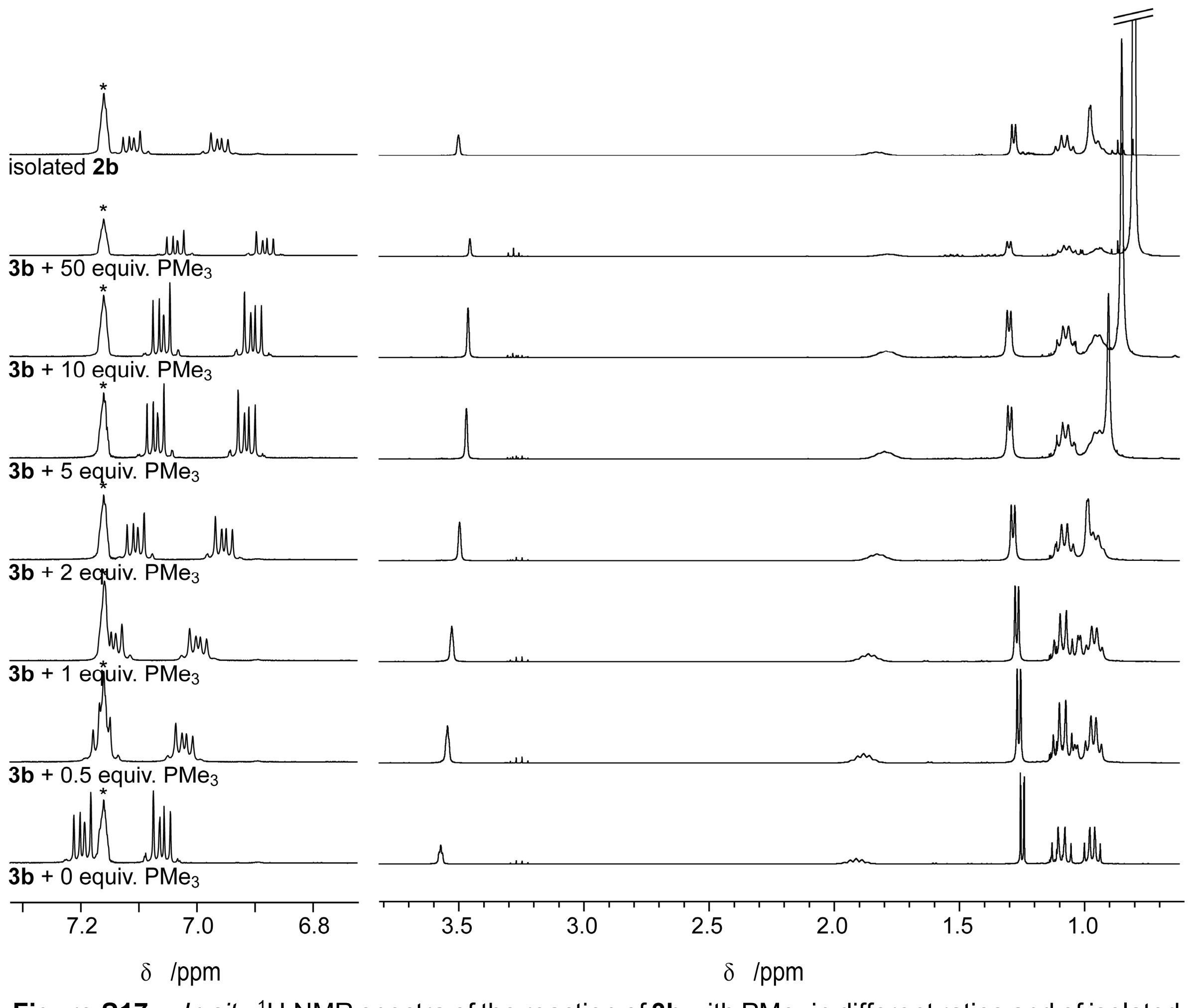

Figure S17: *In situ* ^{1}H NMR spectra of the reaction of **3b** with PMe_3 in different ratios and of isolated **2b** [(Me_3P)$_3$Rh–Cl] (300.3 MHz, C_6D_6, rt, * C_6D_5H).

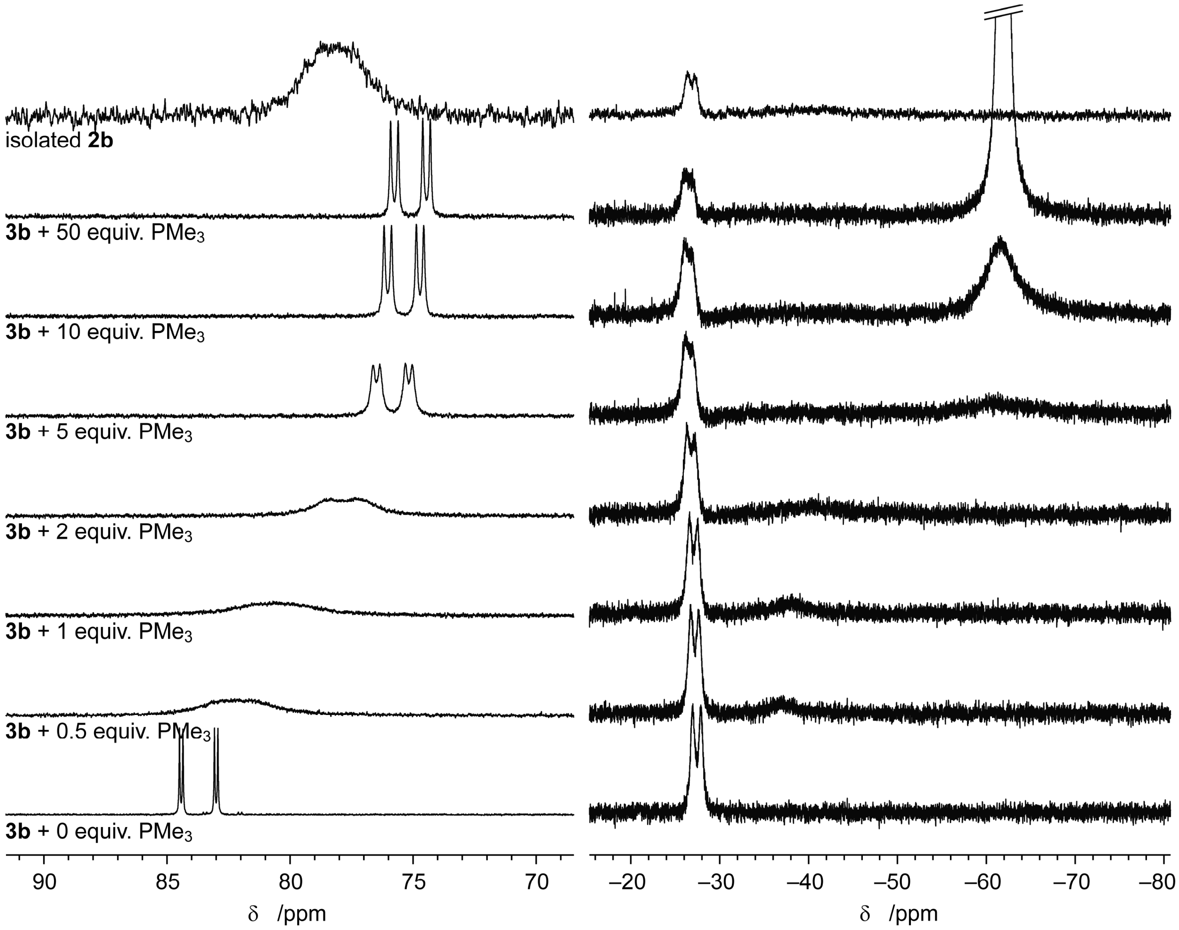

Figure S18: *In situ* $^{31}P\{^{1}H\}$ NMR spectra of the reaction of **3b** with PMe_3 in different ratios and of isolated **2b** (121.6 MHz, C_6D_6, rt).

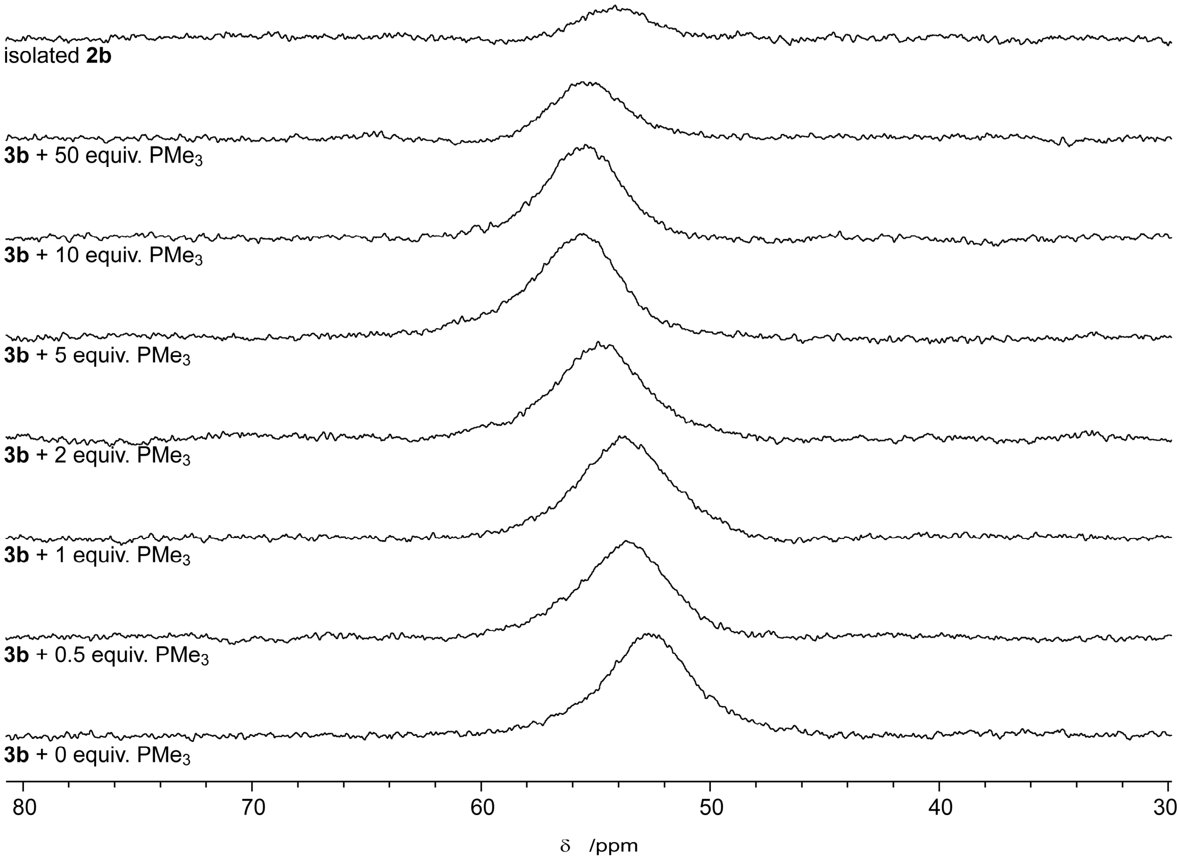

Figure S19: *In situ* $^{11}B\{^1H\}$ NMR spectra of the reaction of **3b** with PMe_3 in different ratios and of isolated **2b** (96.3 MHz, C_6D_6, rt).

Reaction of **3b** with Me_3P to **2b** – UV-Vis experiments

In a nitrogen filled glovebox [(d(CH_2P(*i*Pr)$_2$)abB)Rh(PMe_3)] (**3b**) (1 mg, 1.8 µmol, 1 equiv.) was dissolved in *n*-pentane (50 mL) and an UV-Vis spectrum was recorded (10 mm optical length). A solution of PMe_3 (45.5 µL, 34.0 mg, 0.45 mmol, 250 equiv.) in *n*-pentane (15 mL total volume) was prepared. A portion of 30 µL of the PMe_3 solution (i.e. 0.5 equiv. PMe_3) was added to the solution of **3b** and a UV/Vis spectrum was recorded. Subsequently additional 30, 60, 180, 300, 2400 and 3000 µL of the PMe_3 solution (i.e. 1, 2, 5, 10, 50 and 100 equiv. PMe_3) were added to the complex solution and UV-Vis spectra were recorded. Finally pure PMe_3 (18.2 µL, 13.6 mg, 0.18 mmol, 100 equiv.; i.e. 200 equiv. in total) was added to the reaction solution and a UV-Vis spectrum was recorded (Figure S20).

Data Analysis: To estimate the amounts of **2b** and **3b** present in the reaction mixtures depending on the amount of PMe_3 added we chose an approximate approach: Having the UV-vis spectrum of pure **3b**, from isolated material, and of supposedly pure **2b** from the spectra with the maximum amount of PMe_3 added (**2b**: 200 equiv., *m* molar equivalents with respect to **3b** + **2b**) under the assumption of complete conversion to **2b** and after abstraction of the spectrum for the respective amount of free PMe_3 present (199 equiv.), we were able to simulate all intermediate spectra reasonably well as weighted sum of the spectra for **2b** (1–*n* molar equivalents), **3b** (*n* molar equivalents, initial value *n* = 1) and free PMe_3 (*m*–(1–*n*) molar equivalents) (Figure S21).

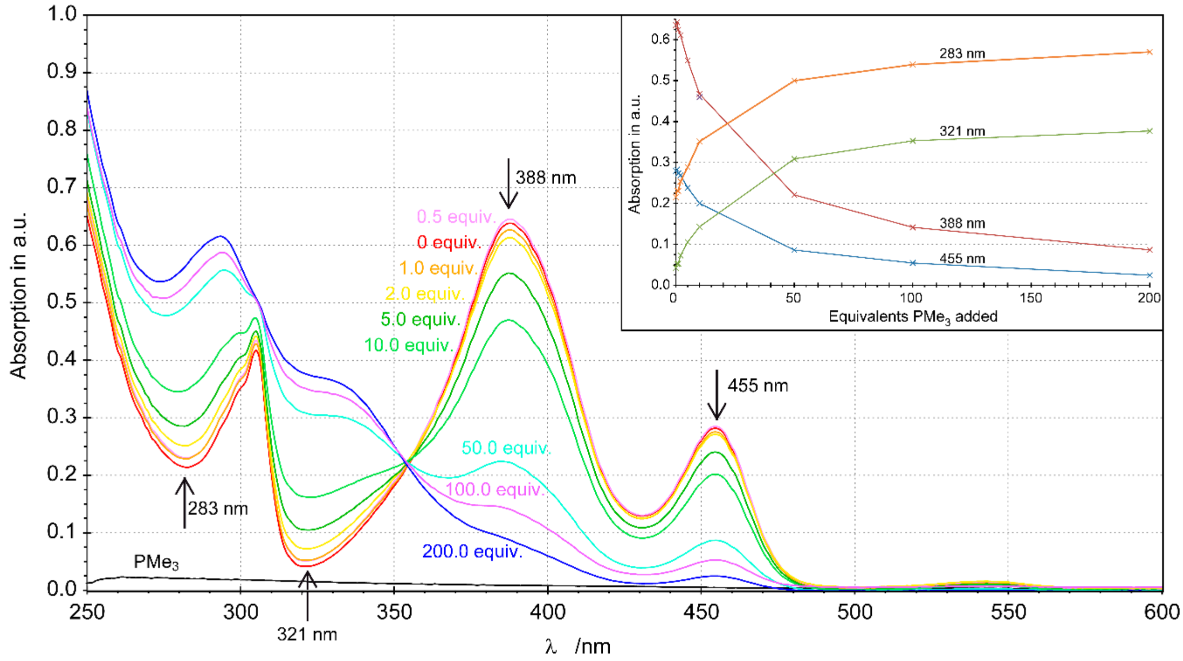

Figure S20: *In situ* UV-Vis spectra of the reaction of **3b** with different amounts of added PMe_3 (*n*-pentane, rt).

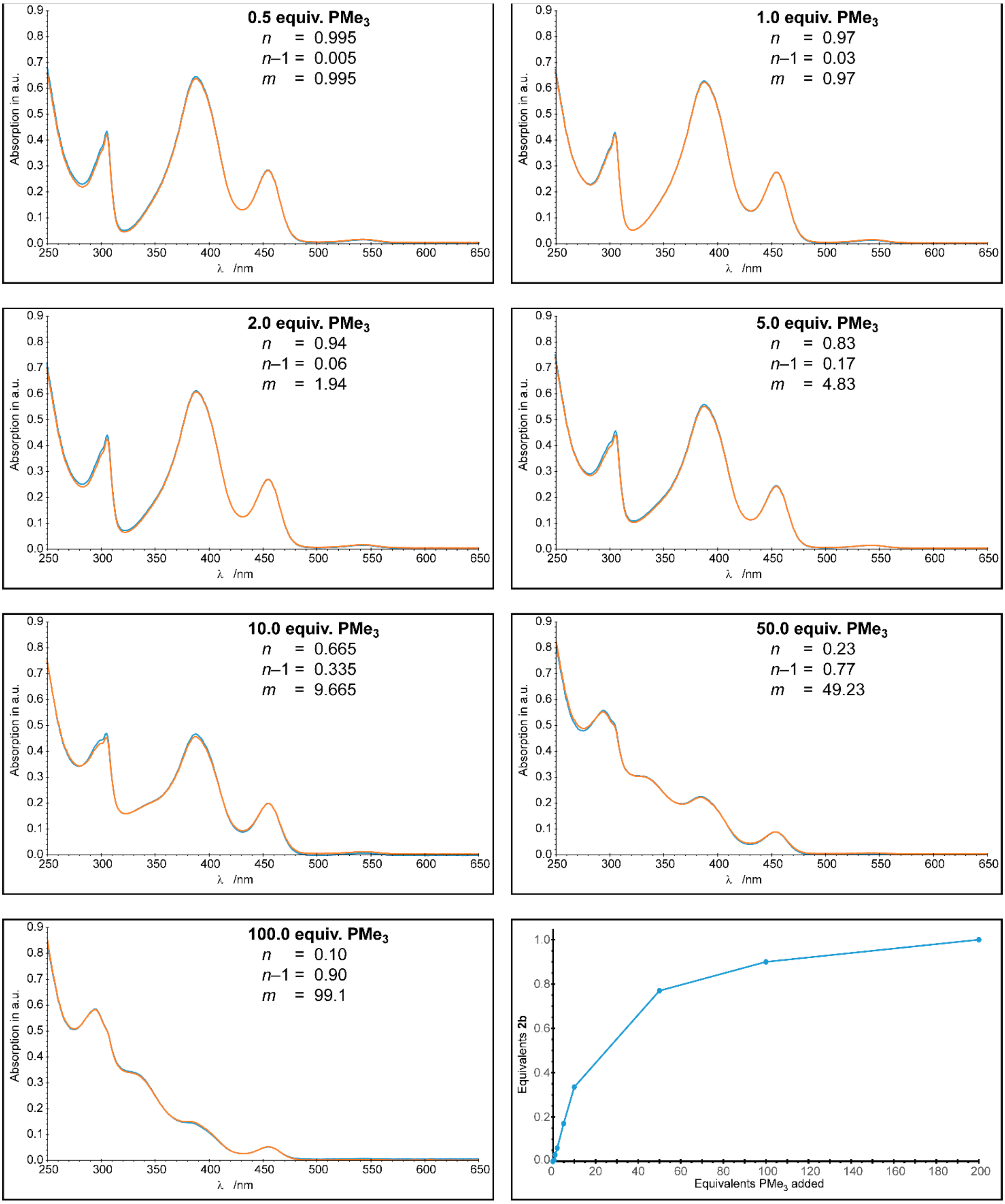

Figure S21: Experimental (blue) and simulated (orange, see above) UV-Vis spectra and amount of **2b** formed for the reaction of **3b** with different amounts of added PMe_3.

e. [(d(CH2P(*i*Pr)2)abB)IrCl(Bpin)] (5c)

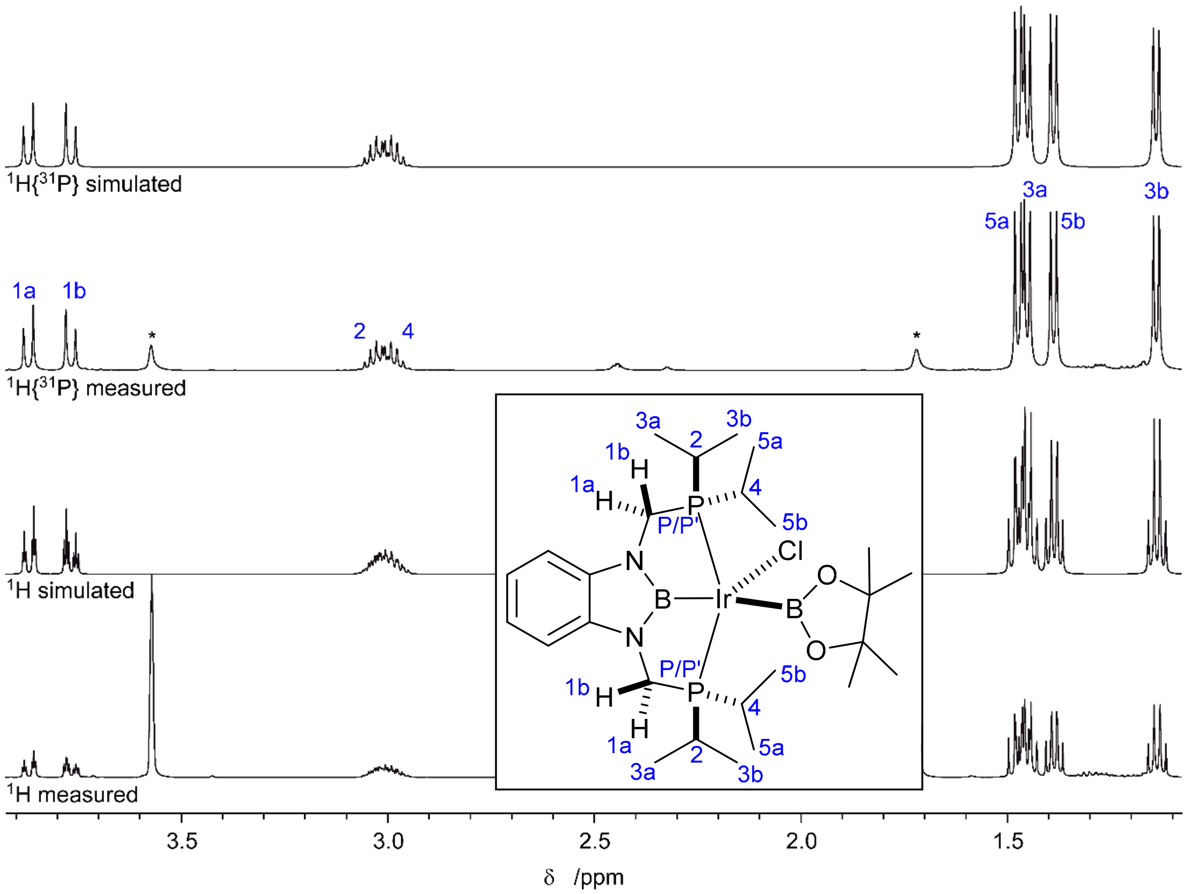

Figure S22: Section of the ^{1}H and $^{1}H\{^{31}P\}$ spectra of **5c** and simulation of selected signals (500.3 MHz, THF-d_8 (*), rt).

Table S4: Coupling constants (Hz) used in the simulation of the ^{1}H NMR and $^{1}H\{^{31}P\}$ spectrum of **5c** (Figure S18).

Signal (δ/ppm)	1a (3.87)	1b (3.77)	2 (3.03)	4 (2.99)	3a (1.45)	3b (1.14)	5a (1.47)	5b (1.39)	P/P'
1a (3.87)	X	11.60							2.0/2.0
1b (3.77)	11.60	X							3.0/3.0
2 (3.02)			X		7.17	7.07			3.4/2.2
4 (2.99)				X			7.59	7.26	4.8/5.8
3a (1.45)			7.17		X				7.4/7.4
3b (1.14)			7.07			X			7.0/7.0
5a (1.47)				7.59			X		7.9/9.0
5b (1.39)				7.26				X	6.5/6.5
P/P'	2.0/2.0	3.0/3.0	3.4/2.2	4.8/5.8	7.4/7.4	7.0/7.0	7.9/9.0	6.5/6.5	X

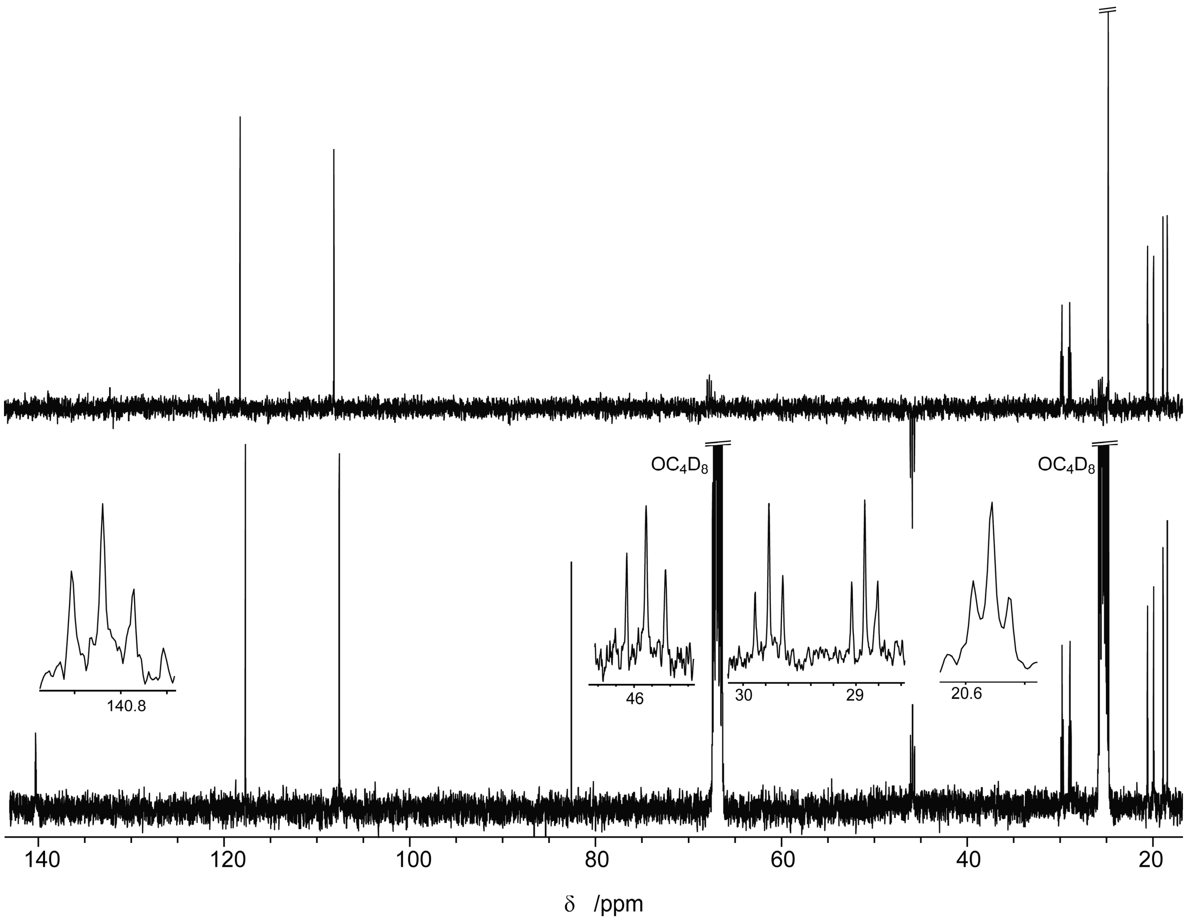

Figure S23: $^{13}C\{^1H\}$ (bottom) and $^{13}C\{^1H\}$-DEPT NMR (top) spectrum of **5c** (100.7 MHz, THF-d_8, rt).

f. [(d(CH2P(*i*Pr)2)abB)IrCl(Bpin)(PMe3)] (6c)

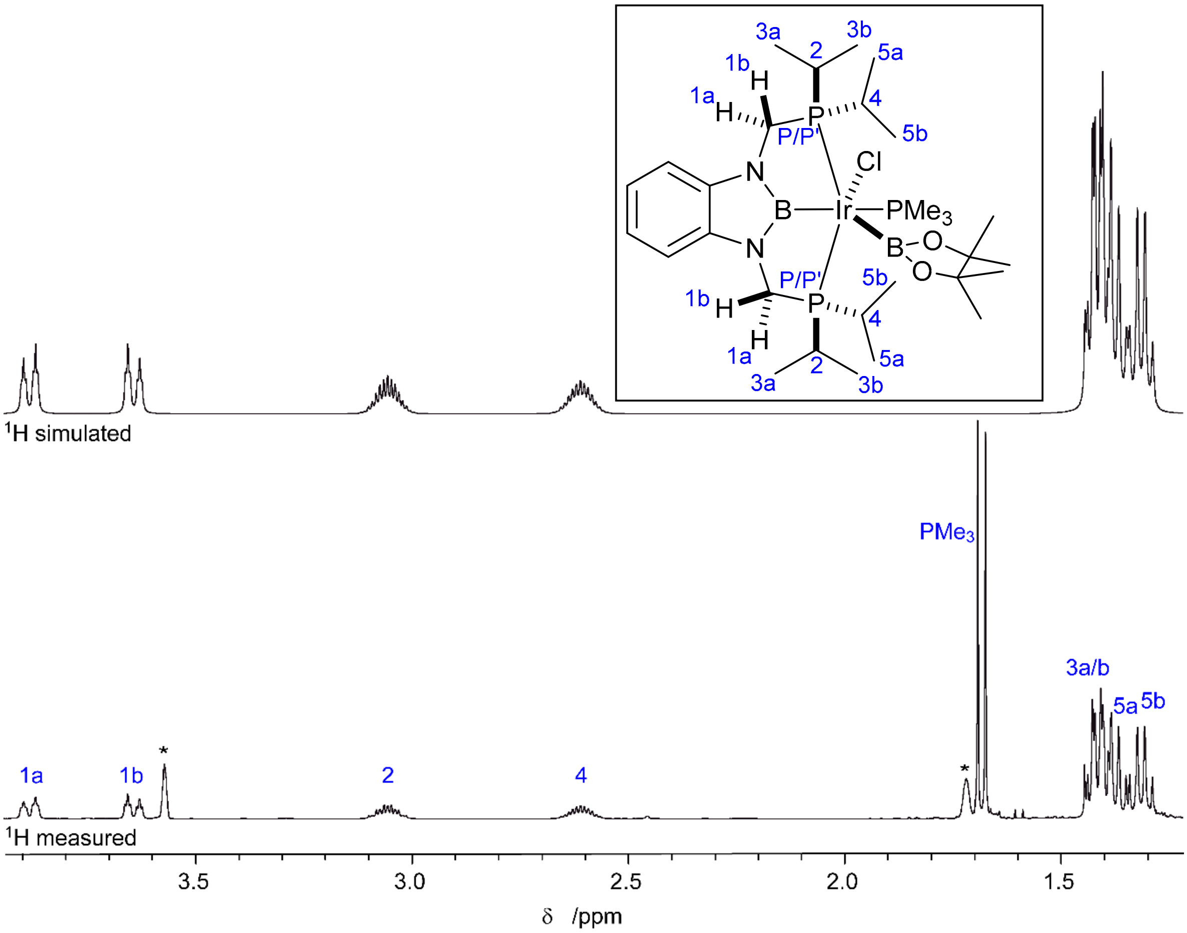

Figure S24: Section of the ^{1}H spectra of **6c** and simulation of selected signals (400.4 MHz, THF-d_8, rt,* OC_4D_7H).

Table S5: Coupling constants (Hz) used in the simulation of the ^{1}H NMR spectrum of **6c** (Figure S20).

Signal (δ/ppm)	1a (3.88)	1b (3.64)	2 (3.06)	4 (2.61)	3a (1.42)	3b (1.41)	5a (1.38)	5b (1.31)	P/P'
1a (3.88)	X	11.00							2.2/2.2
1b (3.64)	11.00	X							2.2/2.2
2 (3.06)			X		7.05	7.05			3.5/3.5
4 (2.61)				X			7.20	7.20	3.7/3.7
3a (1.42)			7.05		X				7.0/7.0
3b (1.41)			7.05			X			7.0/7.0
5a (1.38)				7.20			X		7.0/7.0
5b (1.31)				7.20				X	7.1/7.1
P/P'	2.2/2.2	2.2/2.2	3.5/3.5	3.7/3.7	7.0/7.0	7.0/7.0	7.0/7.0	7.1/7.1	X

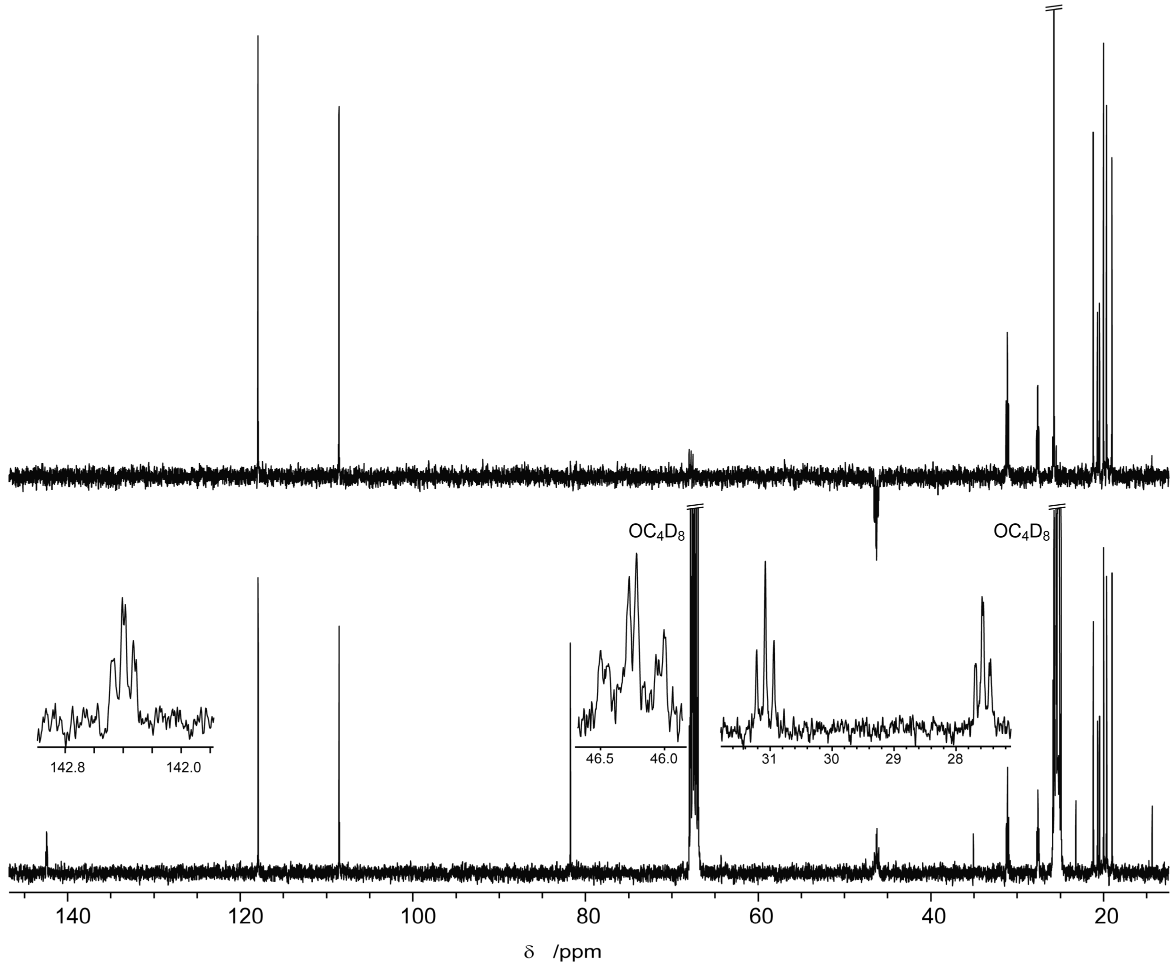

Figure S25: $^{13}C\{^{1}H\}$ (bottom) and $^{13}C\{^{1}H\}$-DEPT NMR (top) spectrum of **6c** (100.7 MHz, THF-d_8, rt).

g. [(d(CH2P(*i*Pr)2)abB)Ir(PMe3)2] (2c)

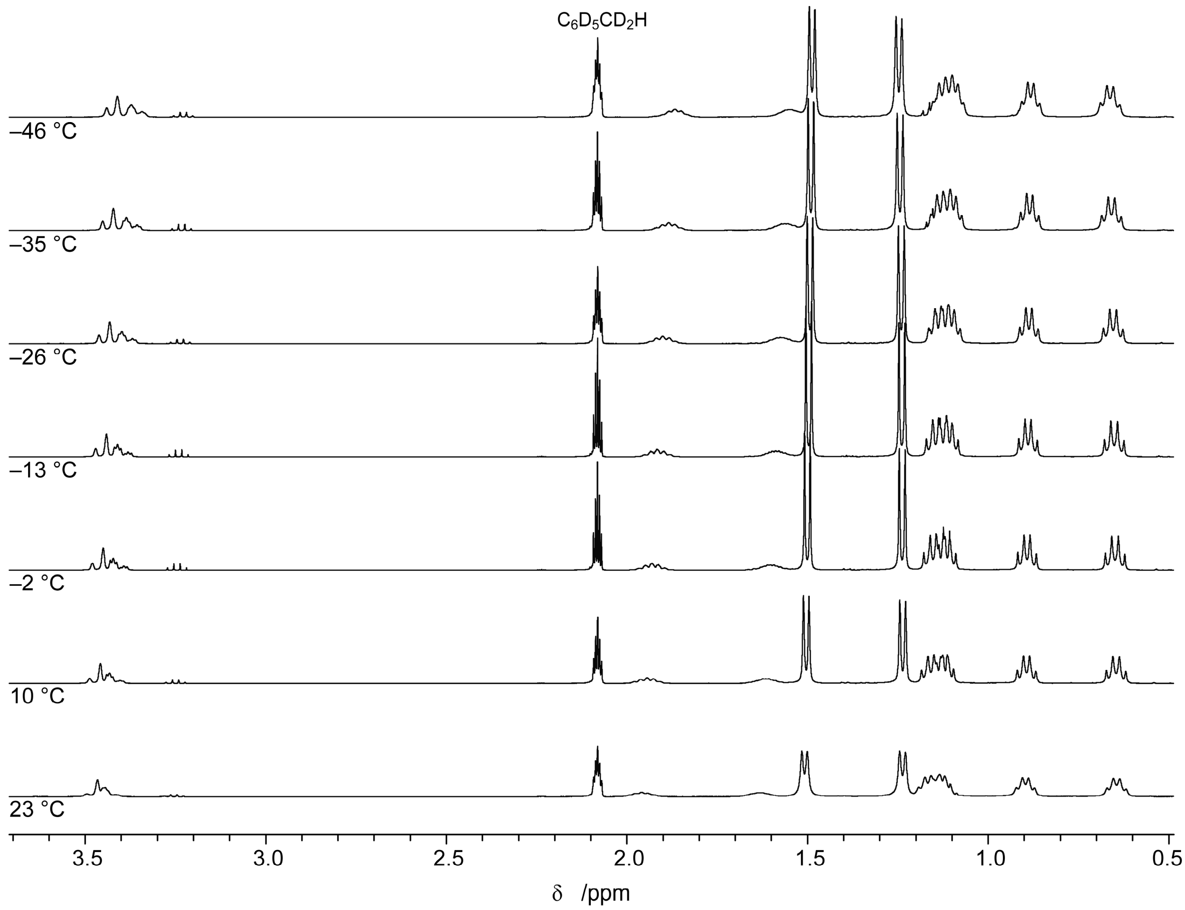

Figure S26: Section of the VT-^{1}H spectra of **2c** (400.4 MHz, PhMe-d_8).

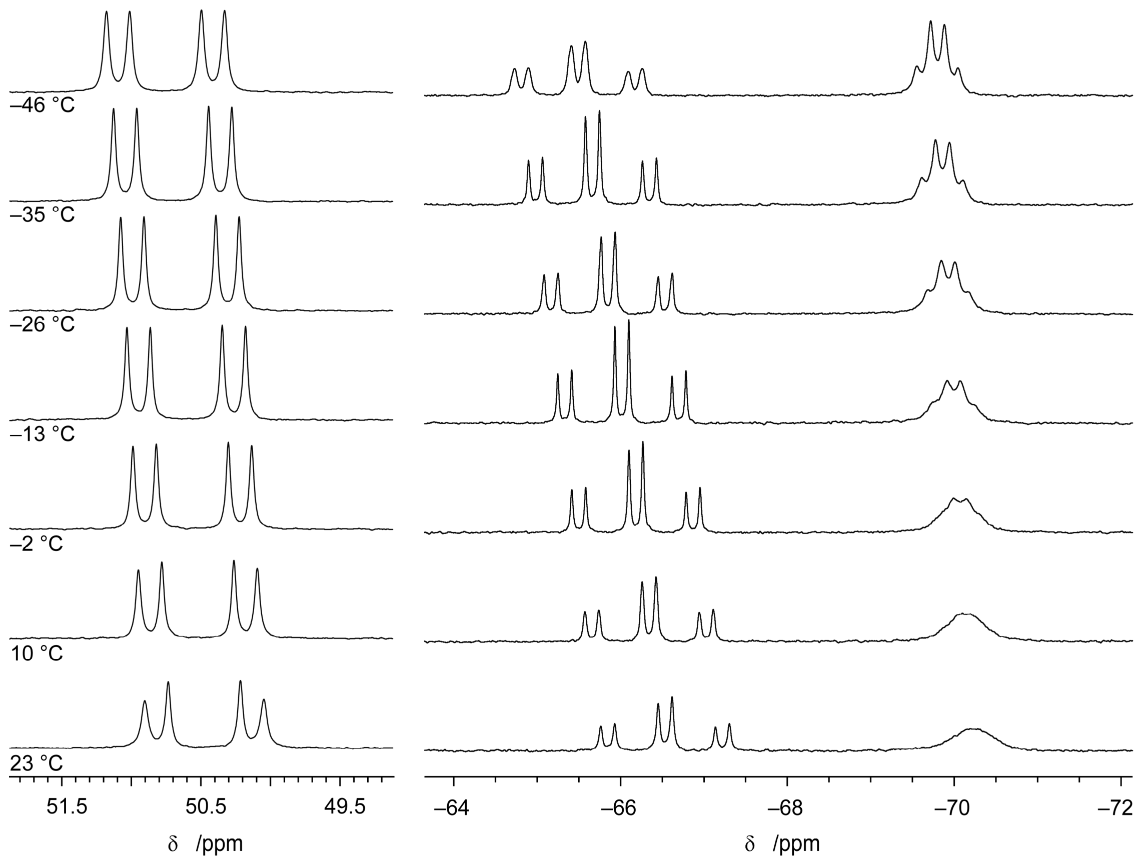

Figure S27: Sections of the VT-$^{31}P\{^{1}H\}$ spectra of **2c** (162.1 MHz, PhMe-d_8).

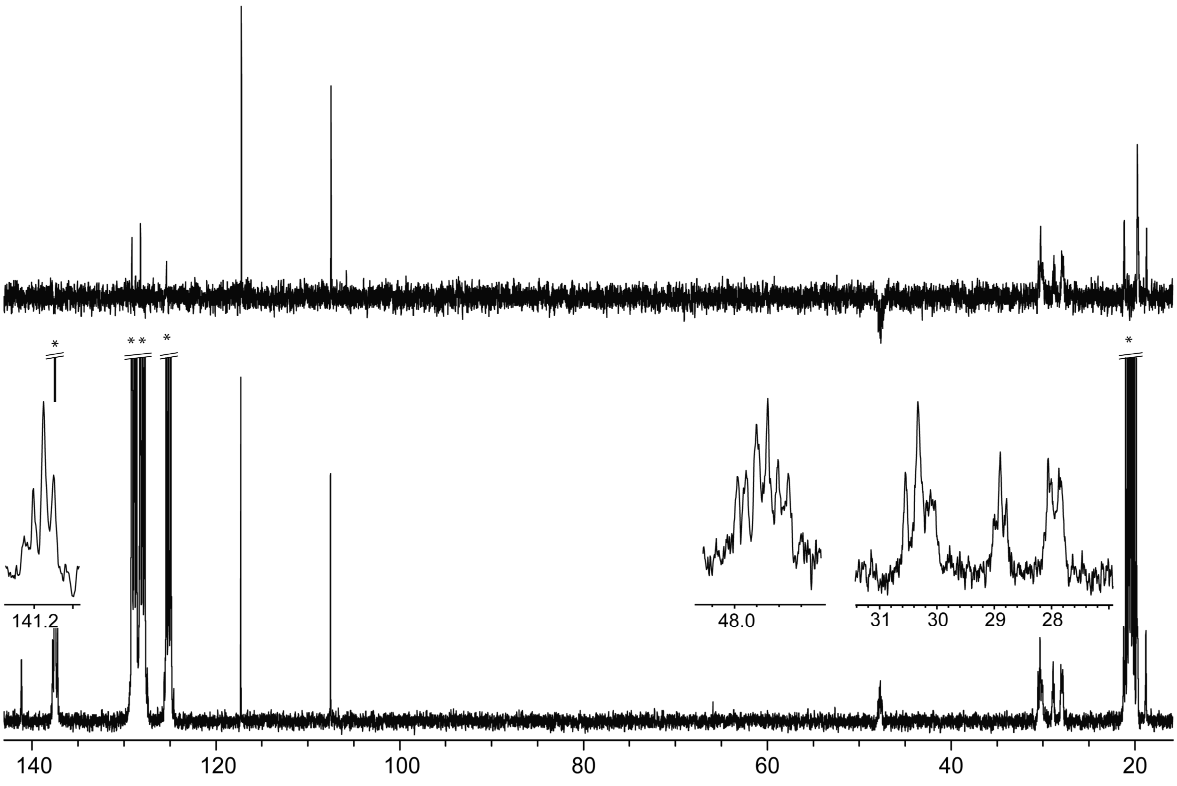

Figure S28: $^{13}C\{^{1}H\}$ (bottom) and $^{13}C\{^{1}H\}$-DEPT NMR (top) spectrum of **2c** (100.7 MHz, PhMe-d_8 (*), rt).

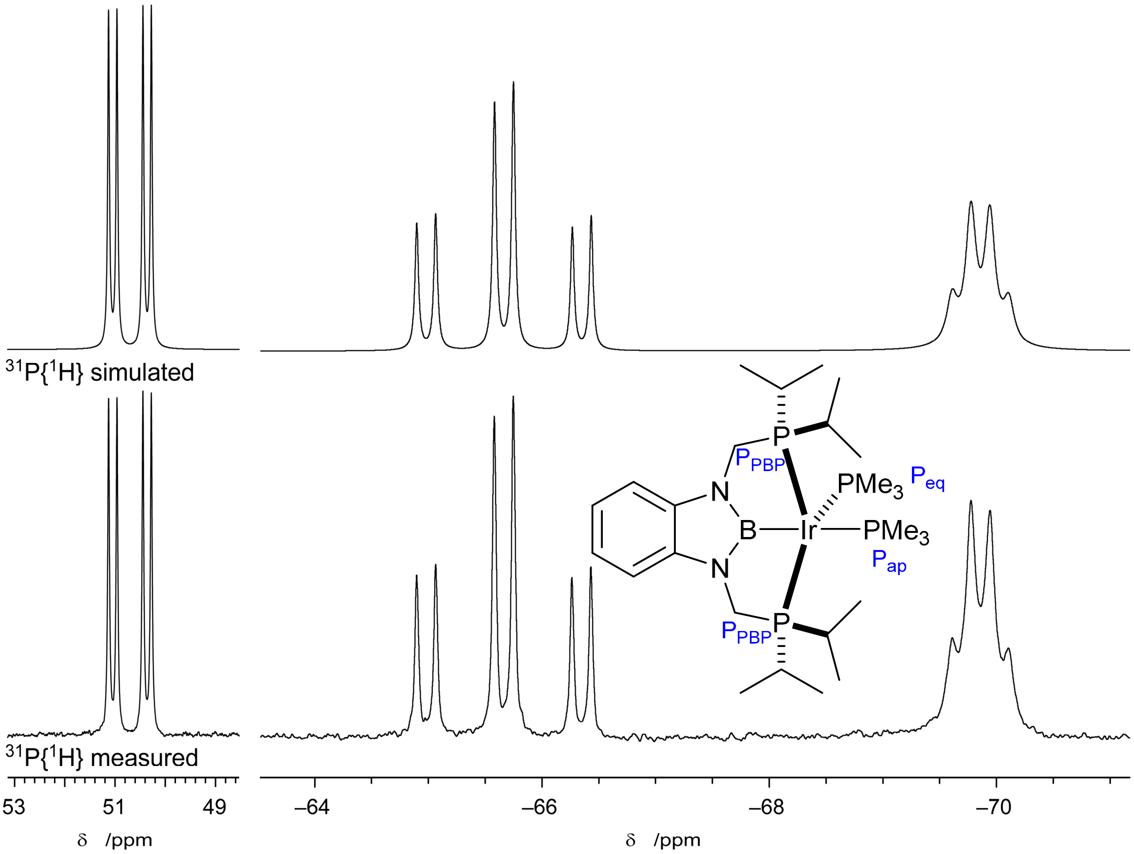

Figure S29: Measured ^{31}P{^{1}H} spectrum of **2c** at –35 °C (bottom) and simulated spectrum (top) (162.1 MHz, PhMe-d8).

Table S6: Coupling constants (Hz) used in the simulation of the ^{31}P{^{1}H} NMR spectrum of **2c** (Figure S22).

Signal (δ/ppm)	P_{PBP} (50.7)	P' (–65.6)	P (–69.9)
P_{PBP} (50.7)	X	112	27
P' (–65.6)	112	X	27
P (–69.9)	27	27	X

Reaction of **3c** with Me_3P to **2c** – UV-Vis experiments

In a nitrogen filled glovebox [(d(CH_2P(*i*Pr)$_2$)abB)Ir(PMe_3)] (**3c**) (1 mg, 1.6 µmol, 1 equiv.) was dissolved in *n*-pentane (50 mL) and an UV-Vis spectrum was recorded (10 mm optical length). A solution of PMe_3 (39.4 µL, 29.5 mg, 0.39 mmol, 250 equiv.) in *n*-pentane (15 mL total volume) was prepared. A portion of 30 µL of the PMe_3 solution (i.e. 0.5 equiv. PMe_3) was added to the solution of **3c** and a UV-Vis spectrum was recorded. Subsequently additional 30, 60, 180, 300 and 2400 µL of the PMe_3 solution (i.e. 1, 2, 5, 10 and 50 equiv. PMe_3) were added to the complex solution and UV-Vis spectra were recorded (Figure S30).

Data Analysis: To estimate the amounts of **2c** and **3c** present in the reaction mixtures depending on the amount of PMe_3 added we chose an approximate approach: Having the UV-vis spectrum of pure **3c**, from isolated material, and of supposedly pure **2c** from the spectra with the maximum amount of PMe_3 added (**2c**: 50 equiv., *m* molar equivalents with respect to **3c** + **2c**) under the assumption of complete conversion to **2c** and after abstraction of the spectrum for the respective amount of free PMe_3 present (49 equiv.), we were able to simulate all intermediate spectra reasonably well as weighted sum of the spectra for **2c** (1–*n* molar equivalents), **3c** (*n* molar equivalents, initial value *n* = 1) and free PMe_3 (*m*–(1–*n*) molar equivalents) (Figure S31).

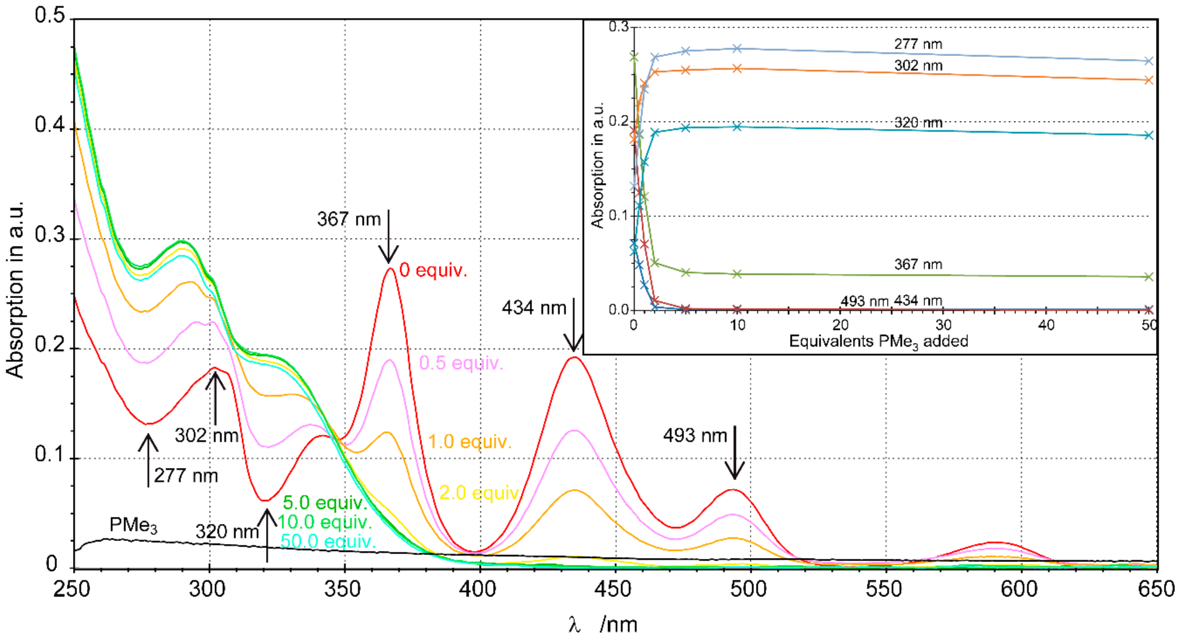

Figure S30: *In situ* UV-Vis spectra of the reaction of **3c** with different amounts of added PMe_3 (*n*-pentane, rt).

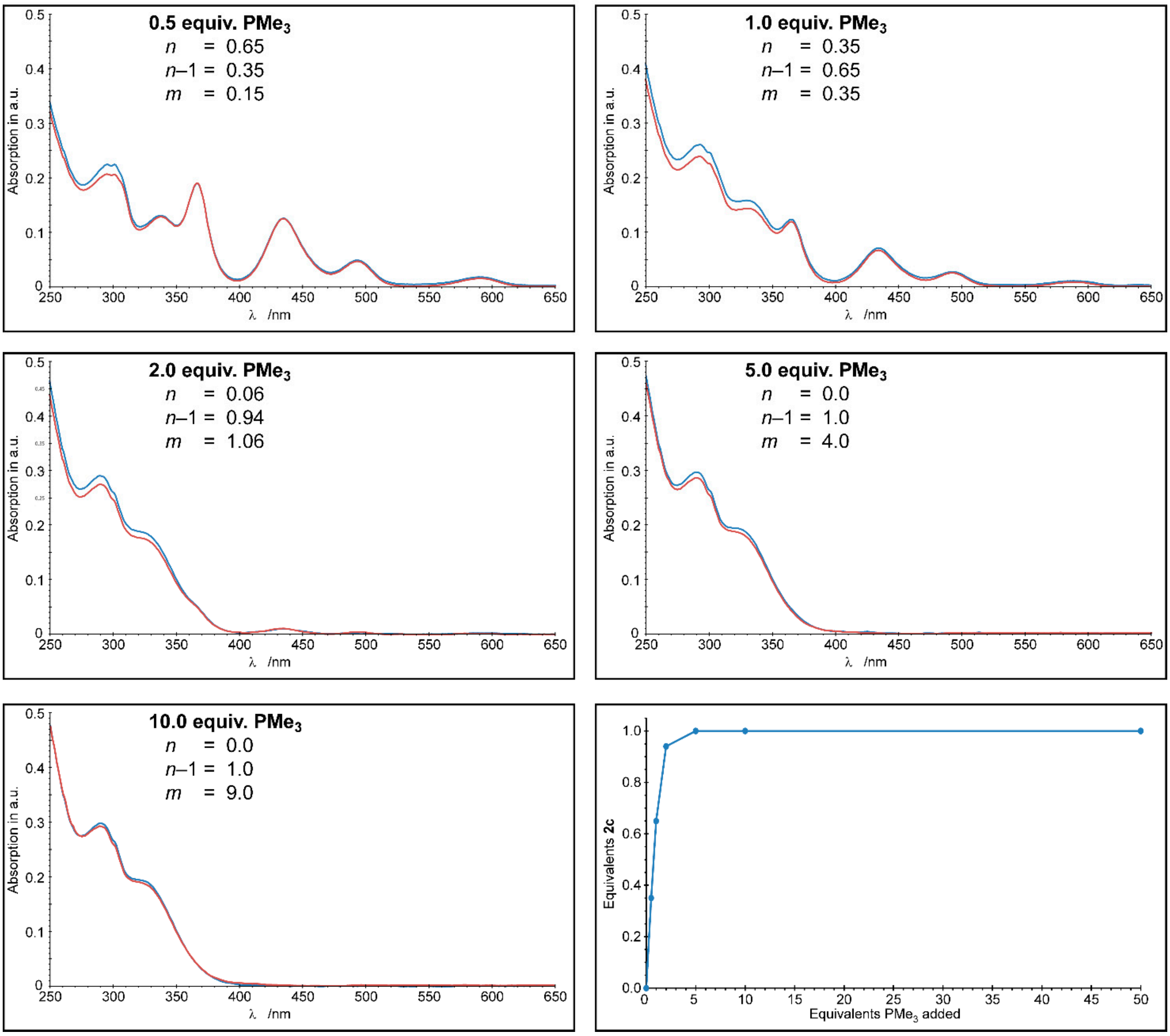

Figure S31: Experimental (blue) and simulated (orange, see above) UV-Vis spectra and amount of **2c** formed for the reaction of **3c** with different amounts of added PMe_3.

h. [(d(CH_2P(*i*Pr)$_2$)abB)Ir–PMe_3] (3c)

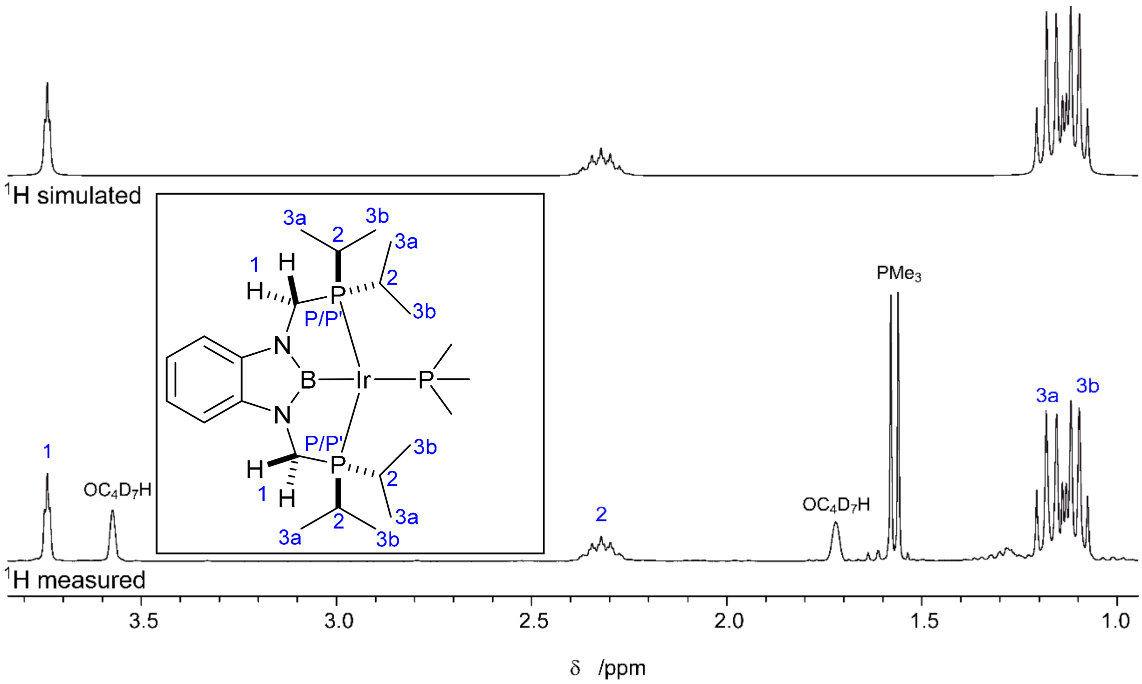

Figure S32: Section of the ^1H and $^1H\{^{31}P\}$ spectra of **3c** and simulation of selected signals (300.1 MHz, rt, THF-d_8).

Table S7: Coupling constants (Hz) used in the simulation of the ^1H NMR and $^1H\{^{31}P\}$ spectrum of **3c** (Figure S23).

Signal (δ/ppm)	1 (3.74)	2 (2.32)	3a (1.17)	3b (1.11)	P/P’
1 (3.74)	X				2.0/2.0
2 (2.32)		X	7.00	7.00	2.2/2.2
3a (1.17)		7.00	X		7.9/7.9
3b (1.11)		7.00		X	6.1/6.1
P/P’	2.0/2.0	2.2/2.2	7.9/7.9	6.1/6.1	X

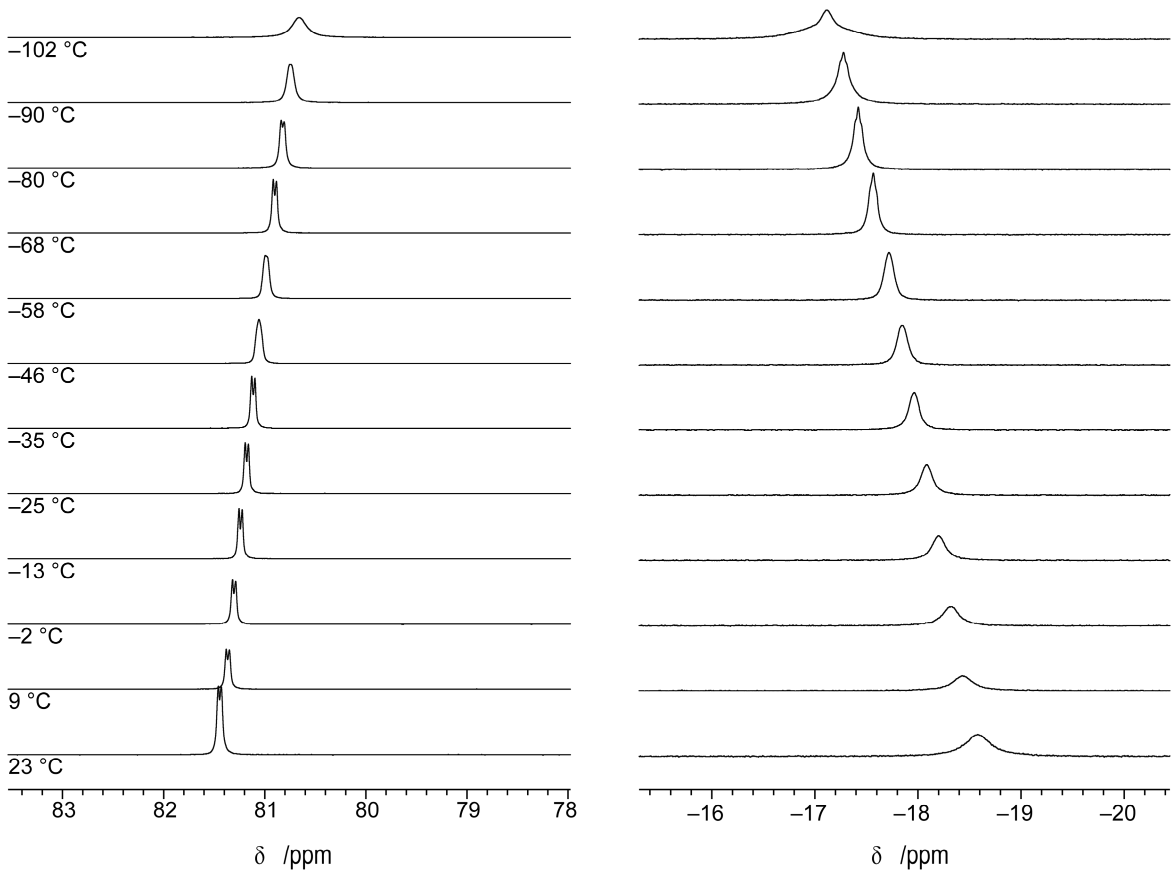

Figure S33: Sections of the VT-$^{31}P\{^{1}H\}$ spectra of **3c** (162.1 MHz, PhMe-d_8).

Reaction of **6c** with KO*t*Bu

In a nitrogen filled glovebox, **6c** (10 mg, 12 μmol, 1 equiv.) was dissolved in THF-d_8 (0.7 mL) and KO*t*Bu (1.4 mg, 12 μmol, 1 equiv.) was added. A series of NMR spectra was recorded after 5 min, 2 h, 6 h and 3 d.

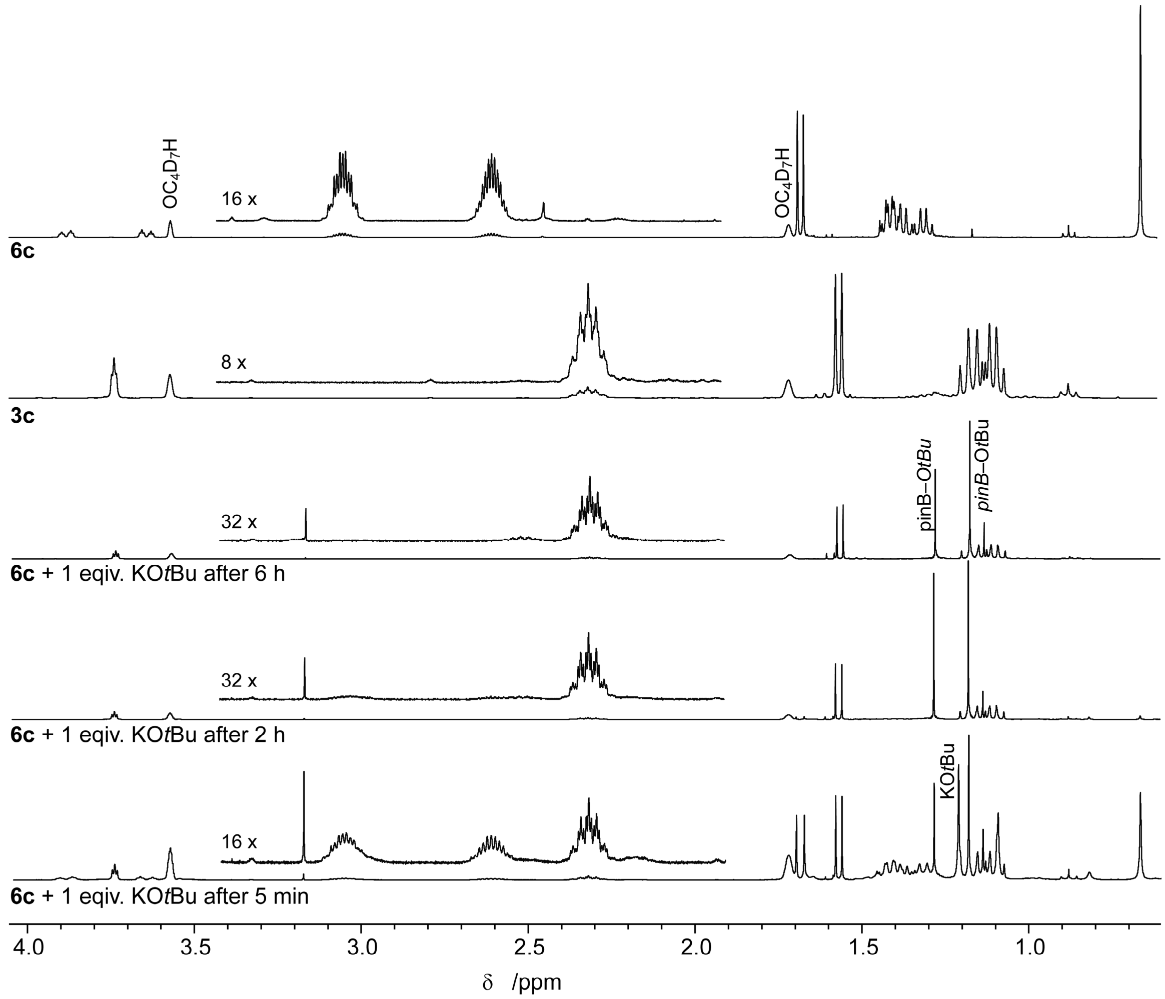

Figure S34: *In situ* ^{1}H spectra of the reaction **6c** with KO*t*Bu (300.3 MHz, THF-d_8, rt).

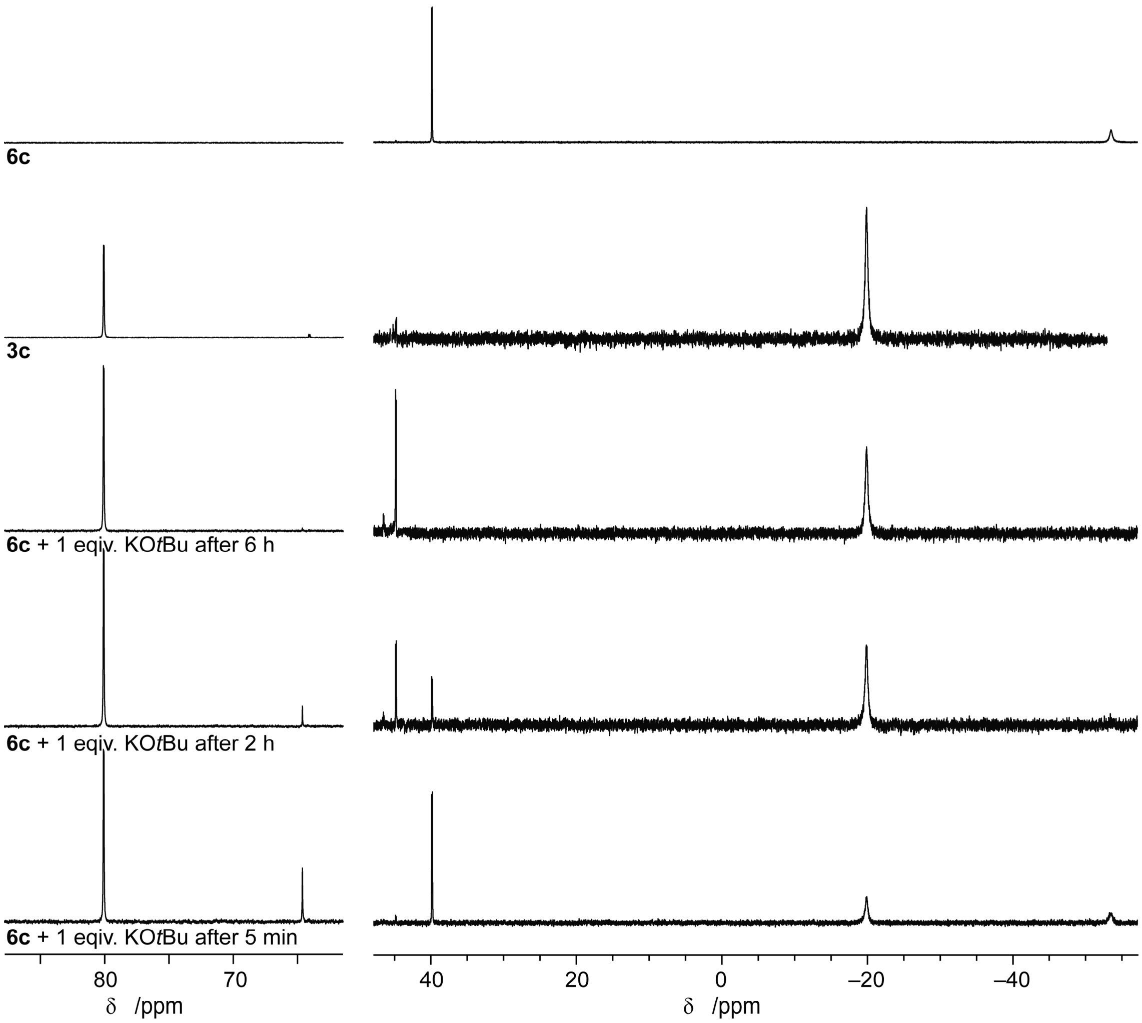

Figure S35: *In situ* $^{31}P\{^1H\}$ spectra of the reaction **6c** with KO*t*Bu (121.6, THF-d_8, rt).

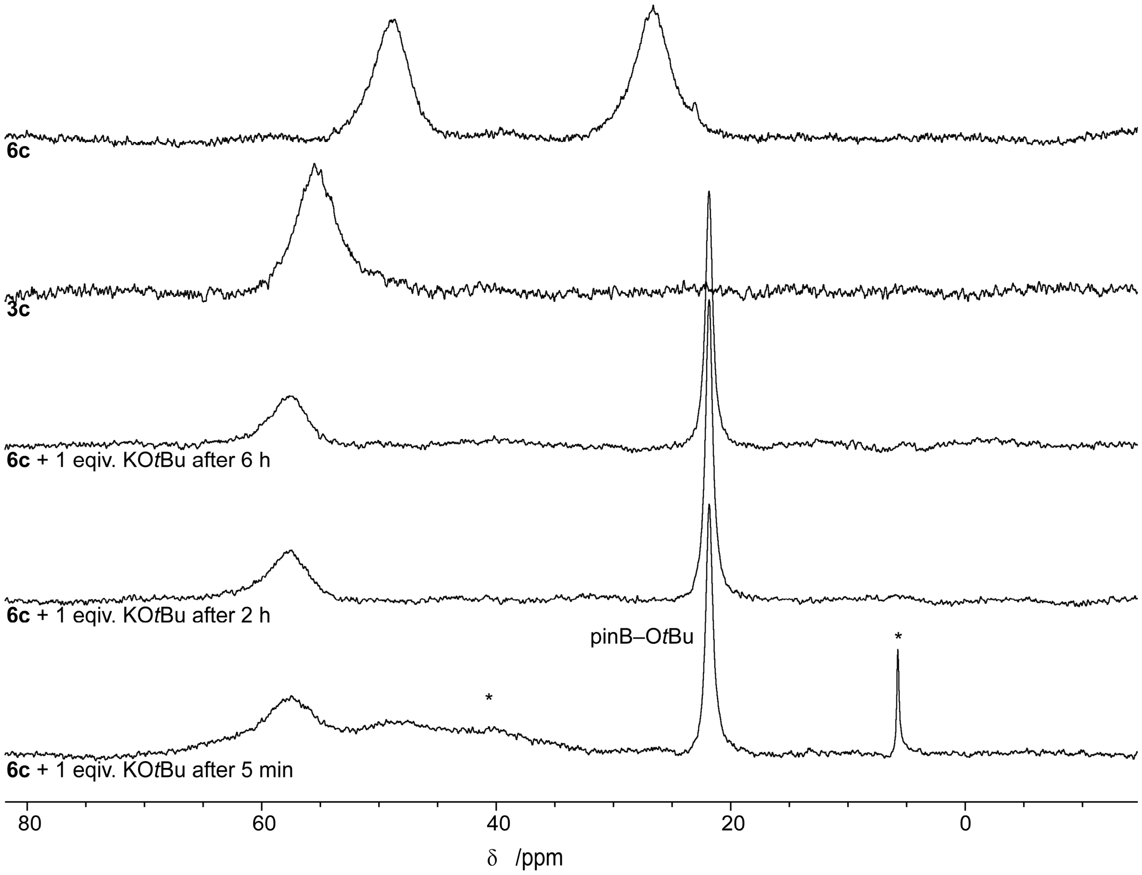

Figure S36: *In situ* $^{11}B\{^1H\}$ spectra of the reaction **6c** with KO*t*Bu (96.3 MHz, THF-d$_8$, rt, * unidentified species, see main text).

2. Crystallographic Data

2.a. Crystallographic Data Collection Parameters

Table S8 Crystallographic data collection parameters for [(d(CH$_2$P(*i*Pr)$_2$)abB)Co–(PMe$_3$)$_2$] (**2a**), [(d(CH$_2$P(*i*Pr)$_2$)abB)Co(N$_2$)(PMe$_3$)] (**4a**) and [(d(CH$_2$P(*i*Pr)$_2$)abB)Rh–(PMe$_3$)$_2$] (**2b**).

	**2a**	**4a**[a]	**2b**
Chemical Formula	$C_{26}H_{54}BCoN_2P_4$	$C_{23}H_{45}BCoN_4P_3$	$C_{26}H_{54}BN_2P_4Rh$
Formula mass (g mol^{-1})	588.33	540.28	632.31
Crystal shape	fragment	fragment	fragment of rhomboid
Crystal color	clear bronze	clear brown	clear colourless
Crystal size (mm^3)	0.221 × 0.171 × 0.040	0.098 × 0.076 × 0.045	0.384 × 0.220 × 0.111
Temperature	100(2) K	100(2) K	100(2) K
Radiation	MoKα, 0.71073 Å	CuKα, 1.54184 Å	MoKα, 0.71073 Å
Abs. coefficient (corr.)	0.772 mm^{-1} (multi-scan)	6.502 mm^{-1} (gaussian)	0.766 mm^{-1} (gaussian)
Crystal system	orthorhombic	monoclinic	monoclinic
Space group type (no.)	$Pca2_1$ (29)	$P2_1/c$	$P2_1/n$ (14)
Z, Z'	4, 1	8, 2	4, 1
a (Å)	16.1193(2)	32.8145(14)	10.0365(1)
b (Å)	10.7243(2)	8.8973(3)	18.8007(2)
c (Å)	18.0581(3)	20.1123(7)	16.6707(2)
α	90°	90°	90°
β	90°	106.013(4)°	93.945(1)°
γ	90°	90°	90°
Volume (Å^3)	3121.67(9)	5644.2(4)	3138.19(6)
Refl. collected	255992	12815	237026
unique	16845	12815	16729
observed [$I>2\sigma(I)$]	15083	12414	15153
Data collection ranges	2.26° < θ < 38.38°	4.40° < θ < 78.99°	2.17° < θ < 38.26°
	–28 ≤ h ≤ 27	–41 ≤ h ≤ 41	–17 ≤ h ≤ 16
	–17 ≤ k ≤ 18	–11 ≤ k ≤ 11	–31 ≤ k ≤ 32
	–31 ≤ l ≤ 31	–25 ≤ l ≤ 25	–29 ≤ l ≤ 28
Completeness (to 2θ)	97.8% (76.76°)	98.9% (67.68°)	96.3% (76.52°)
Data / restr. / param.	16845 / 1 / 321	12815	16729 / 0 / 321
R_{int}	0.0505	n/a	0.0365
R_1 [$I > 2\sigma(I)$]	0.0277	0.0625	0.0183
wR_2 (all data)	0.0654	0.1582	0.0475
GoF on F^2	1.048	1.077	1.017
Largest peak/hole (Å^{-3})	0.504 / –0.254	0.790 / –0.995	0.639 / –0.433
Abs. struct. par. (Friedel cov.)	0.000(3) (92.9%)	n/a	n/a
CCDC no.	2269779	2269774	2269781

a The crystal is twinned by 180° rotation about the a* axis (0.91 0 0.41 direct space). The data were integrated to give a data set containing the intensities of both separated components and all overlapping reflection. These data were used for refinement, including a twin factor (HKLF5/BASF). The twin factor refined to 0.368(1).

Table S8 cond. Crystallographic data collection parameters for [(d($CH_2P(iPr)_2$)abB)Rh–PMe_3] (**3b**), [(d($CH_2P(iPr)_2$)abB)Ir–(PMe_3)$_2$] (**2c**) and [(d($CH_2P(iPr)_2$)abB)Ir–PMe_3] (**3c**).

	**3b**	**2c**[a]	**3c**[b]
Chemical Formula	$C_{23}H_{45}BN_2P_3Rh$	$C_{26}H_{54}BN_2P_4Ir$	$C_{23}H_{45}BN_2P_3Ir$
Formula mass (g mol^{-1})	556.24	721.60	645.53
Crystal shape	fragment of block	prism	fragment
Crystal color	clear orange	clear colourless	dark orange
Crystal size (mm^3)	0.544 × 0.341 × 0.165	0.185 × 0.104 × 0.072	0.270 × 0.130 × 0.090
Temperature	100(2) K	100(2) K	100(2) K
Radiation	MoKα, 0.71073 Å	MoKα, 0.71073 Å	MoKα, 0.71073 Å
Abs. coefficient (corr.)	0.816 mm^{-1} (gaussian)	4.516 mm^{-1} (multi-scan)	5.095 mm^{-1} (gaussian)
Crystal system	monoclinic	monoclinic	monoclinic
Space group type (no.)	$P2_1/c$ (14)	$P2_1/n$ (14)	$P2_1/c$ (14)
Z, Z'	4, 1	4, 1	4, 1
a (Å)	9.3811(1)	10.0321(2)	9.3839(1)
b (Å)	18.9012(2)	18.6653(4)	18.8845(3)
c (Å)	15.4230(1)	16.6510(4)	15.4150(1)
α	90°	90°	90°
β	95.066(1)°	93.950(2)°	94.864(1)°
γ	90°	90°	90°
Volume (Å^3)	2724.03(4)	3111.53(12)	2721.86(6)
Refl. collected	440454	168804	267125
unique	22243	13573	17988
observed [$I>2\sigma(I)$]	20179	10934	14787
Data collection ranges	2.17° < θ < 38.26°	2.18° < θ < 34.97°	2.16° < θ < 41.42°
	–18 ≤ h ≤ 18	–16 ≤ h ≤ 16	–17 ≤ h ≤ 17
	–37 ≤ k ≤ 37	–30 ≤ k ≤ 29	–35 ≤ k ≤ 34
	–30 ≤ l ≤ 30	–26 ≤ l ≤ 26	–28 ≤ l ≤ 28
Completeness (to 2θ)	99.8% (55.83°)	99.4% (69.94°)	100.0% (81.0°)
Data / restr. / param.	22243 / 0 / 282	13573 / 0 / 321	17988 / 0 / 282
R_{int}	0.0359	0.0952	0.0619
R_1 [$I > 2\sigma(I)$]	0.0168	0.0356	0.0293
wR_2 (all data)	0.0444	0.0890	0.0677
GoF on F^2	1.054	1.040	1.046
Largest peak/hole (Å^{-3})	0.680 / –0.318	3.746 / –3.477	5.381 / –0.849
Abs. struct. par. (Friedel cov.)	n/a	n/a	n/a
CCDC no.	2269777	2269775	2269776

a The structure contains considerable residual electron density in the proximity of the Ir atom (distance <0.85 Å) that is presumably associated with absorption effects and/or limitations of the IAM in combination with high theta data.

b The structure contains considerable residual electron density that is presumably associated with absorption effects and/or limitations of the IAM in combination with high theta data. Using data only up to a lower resolution the following data are obtained:

Resolution	0.54 (82.8° 2θ)	0.71 (60° 2θ)	0.84 (50° 2θ)
$I/I(\sigma)$	39.2	67.1	79.7
R_{int}	6.19%	4.77%	4.11%
R_1	2.93%	1.71%	1.50%
wR_2	6.77%	4.44%	3.84%
e$^-$-peak/hole	5.4/–0.8	2.4/–0.7	1.2/–0.4

Table S8 cond. Crystallographic data collection parameters for [(d(CH_2P(*i*Pr)$_2$)abB)Ir(Bpin)(Cl)] (**5c**) and [(d(CH_2P(*i*Pr)$_2$)abB)Ir(Bpin)(PMe_3)(Cl)] (**6c**).

	**5c**[a]	**6c**[b]
Chemical Formula	$C_{26}H_{48}B_2N_2O_2P_2IrCl$	$C_{29}H_{57}B_2N_2O_2P_3RhCl$
Formula mass (g mol^{-1})	731.87	807.94
Crystal shape	hexagonal prism	fragment of lath
Crystal color	clear light yellow	clear colourless
Crystal size (mm^3)	0.190 × 0.090 × 0.070	0.332 × 0.059 × 0.031
Temperature	100(2) K	100(2) K
Radiation	MoKα, 0.71073 Å	MoKα, 0.71073 Å
Abs. coefficient (corr.)	4.417 mm^{-1} (gaussian)	3.985 mm^{-1} (gaussian)
Crystal system	monoclinic	monoclinic
Space group type (no.)	$P2_1/c$ (14)	$P2_1/c$ (14)
Z, Z'	4, 1	8, 2
a (Å)	11.2226(2)	21.4999(4)
b (Å)	13.5579(2)	17.7270(3)
c (Å)	21.2351(3)	19.1333(3)
α	90°	90°
β	100.837(1)°	102.270(2)°
γ	90°	90°
Volume (Å^3)	3173.40(9)	7125.7(2)
Refl. collected	170646	353796
unique	14111	26582
observed [$I>2\sigma(I)$]	12268	21120
Data collection ranges	2.28° < θ < 36.00°	2.18° < θ < 33.14°
	–18 ≤ h ≤ 16	–33 ≤ h ≤ 33
	–22 ≤ k ≤ 21	–27 ≤ k ≤ 27
	–34 ≤ l ≤ 34	–26 ≤ l ≤ 29
Completeness (to 2θ)	99.6% (69.00°)	97.9% (66.28°)
Data / restr. / param.	14111 / 13 / 370	26582 / 30 / 867
R_{int}	0.0418	0.0750
R_1 [$I > 2\sigma(I)$]	0.0260	0.0448
wR_2 (all data)	0.0544	0.0881
GoF on F^2	1.051	1.025
Largest peak/hole (Å^{-3})	2.162 / –1.077	7.157 / –3.202
Abs. struct. par. (Friedel cov.)	n/a	n/a
CCDC no.	2269780	2269778

a A disordered pinacol moiety was refined using a split atom model (0.464(4) SOF), applying similarity restraints on the C–C distances (SAME). A common ADP was refined for each disordered atom pair. A disordered *i*Pr group was refined using a split atom model (0.846(6) SOF), applying similarity restraints on the C–C distances (SAME). The minor component was refined only isotropically.

b The structure contains two independent molecules, one of the contains disordered pinacol, PMe_3 and P*i*Pr_2 moieties that were refined using split atom models applying similarity restraints (SAME). For some atom pairs a common ADP was refined (EADP). The structure contains considerable residual electron density that is presumably associated with absorption effects and/or limitations of the IAM in combination with high theta data. Using data only up to a resolution of 0.84 the following data are obtained:

Resolution	0.64 (66.3° 2θ)	0.84 (50° 2θ)
$I/I(\sigma)$	21.5	54.2
R_{int}	7.50%	5.49%
R_1	4.48%	2.69%
wR_2	8.81%	5.96%
e^--peak/hole	7.2/–3.2	3.6/–2.1

2.b. Overlay of 2a with mirror image of 2a

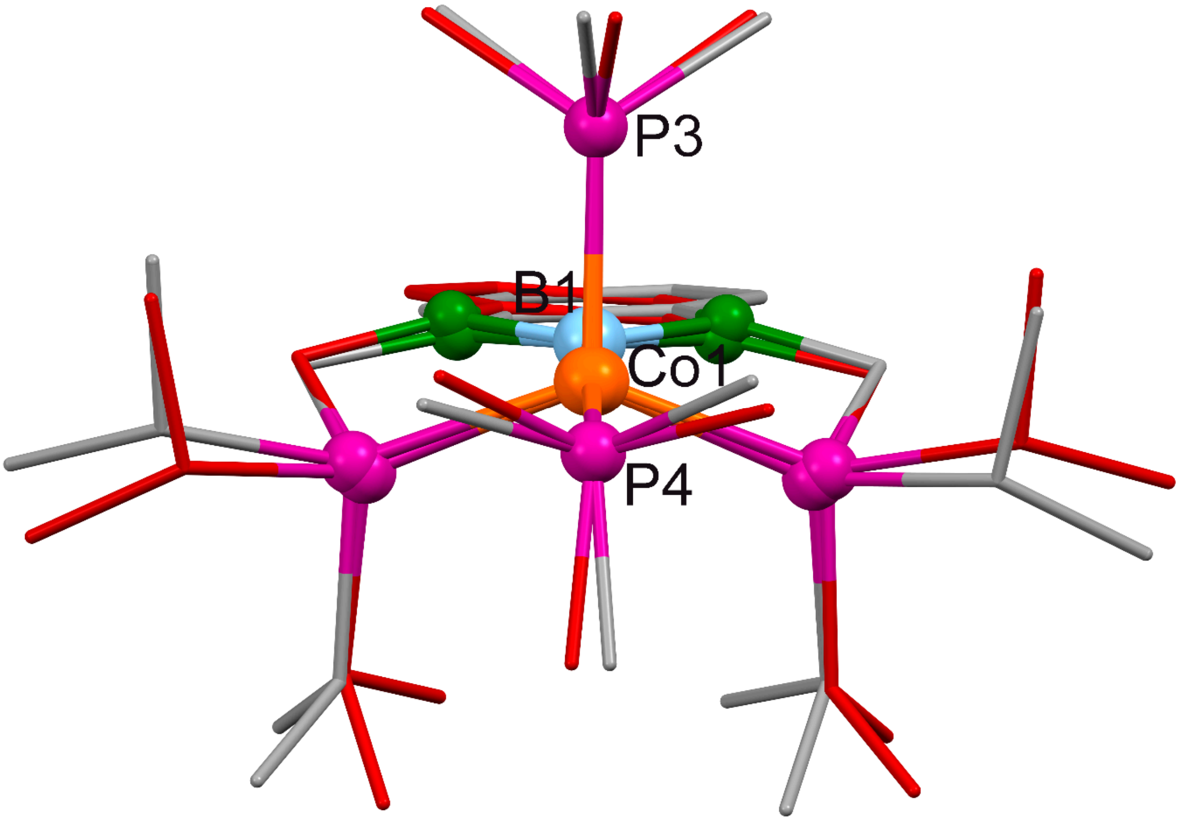

Figure S37: Overlay of the molecular structure of **2a** and its mirror image; B1, P3 P4 on mirror plane.

2.c. Overlay of 2a, 2b and 2c

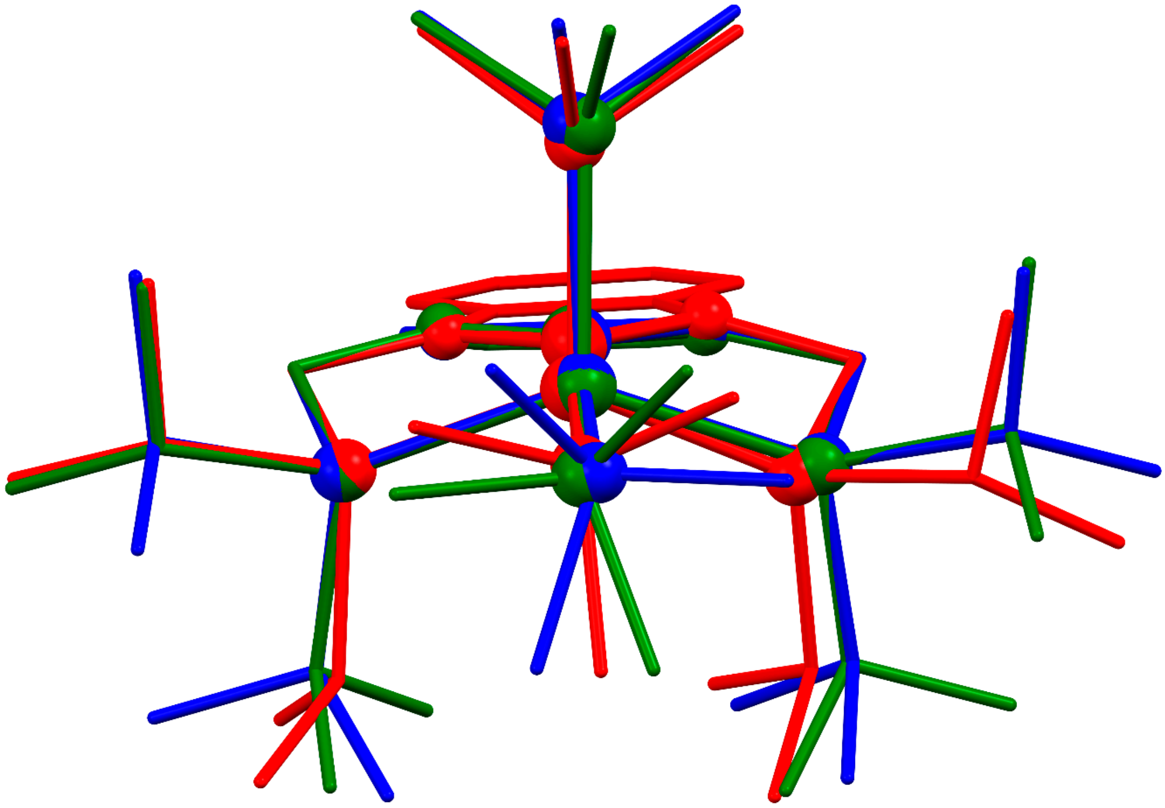

Figure S38: Overlay of the molecular structure of **2a** (red), **2b** (blue) and **2c** (green), best fit Co1/Rh1/Ir1, B1 and pincer P atoms.

2.d. Overlay of 3b and 3c

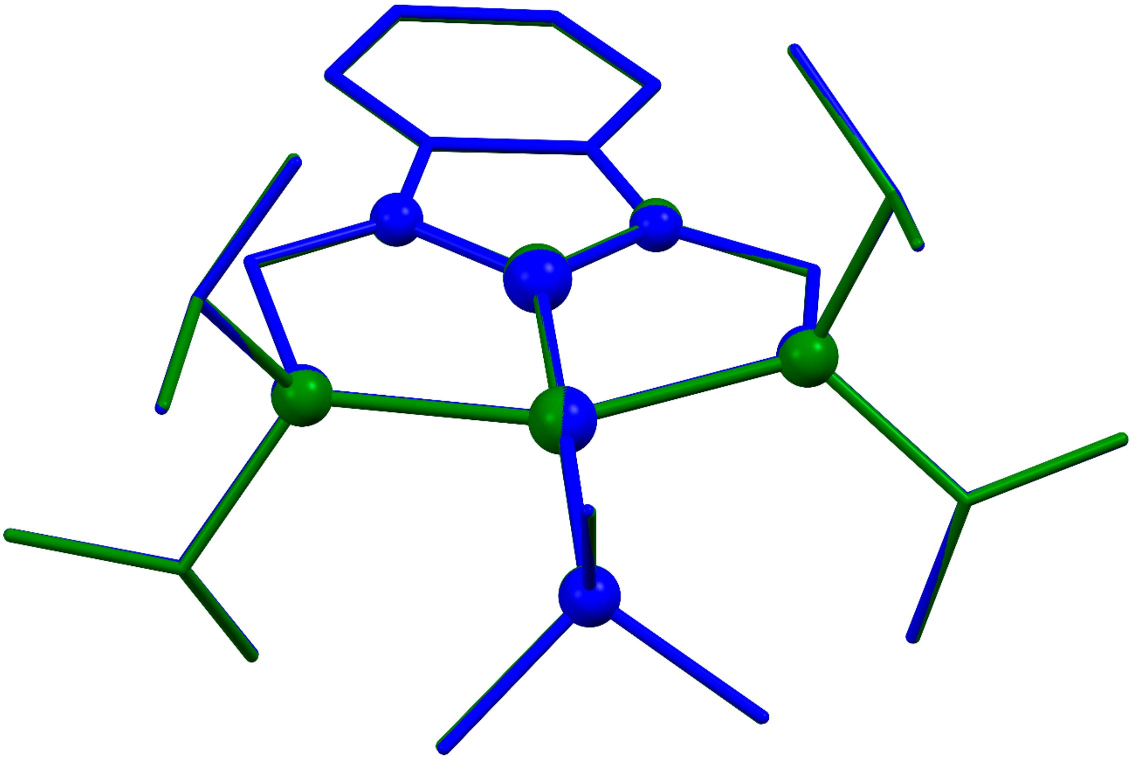

Figure S39: Overlay of the molecular structure of **3b** (blue) and **3c** (green), best fit Rh1/Ir1, B1 and pincer P atoms.

2.e. Overlay of the two independent moelcules in 4a

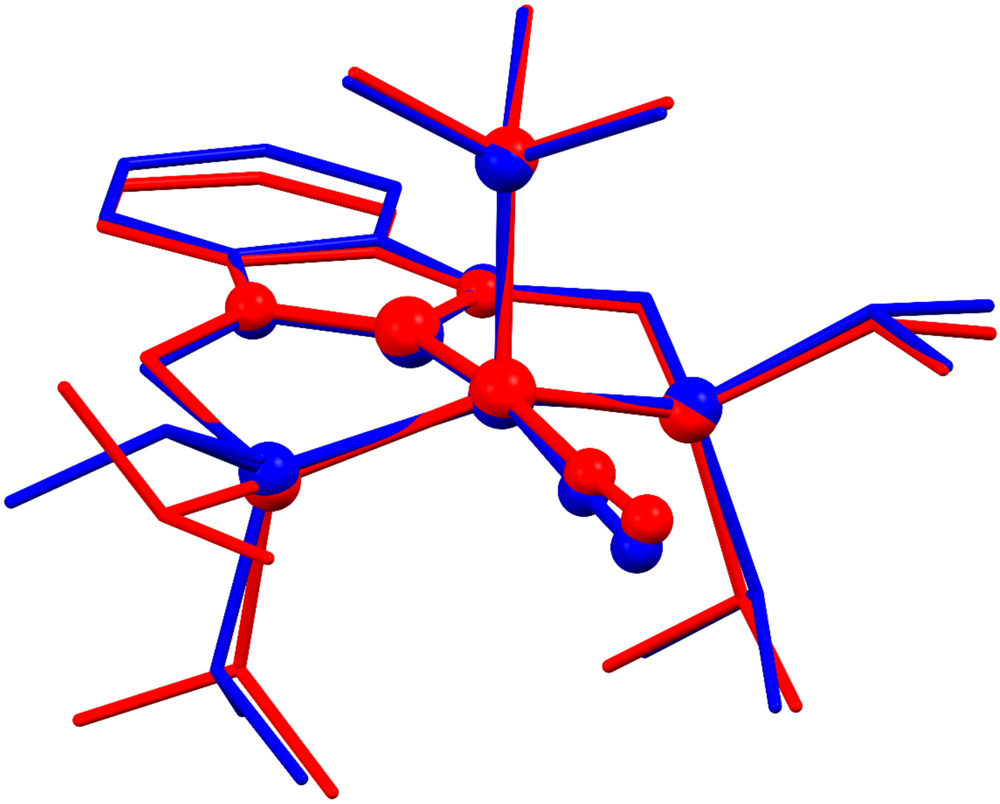

Figure S40: Overlay of the two independent molecules in the solid-state structure of **4a**, best fit of Co, B and P atoms.

3. Computational Data

All quantum chemical calculations have been performed with the Gaussian 09 set of programs [1]. The nonempirical meta-Generalized Gradient Approximation (meta-GGA) hybrid functional by *Tao*, *Perdew*, *Staroverov* and *Scuseria* (TPSSh) was employed for all calculations [2,3]. All atoms were described by a Karlsruhe-type effective core potential basis set of triple zeta quality with one set of polarization functions (def2TZVP) [4]. A closed-shell low-spin configuration (S = 0) was assumed in all calculations. Whenever available the geometry optimizations started from structures obtained from X-ray diffraction data experiments. Unless noted, all geometry optimisations were performed without any imposed symmetry constraints and have been performed with the same combination of electronic structure method and basis set, as the subsequent frequency calculations. The relevant stationary points have been characterised as (local) minima by normal mode analysis based on analytical energy second derivatives; none of them exhibited any imaginary frequencies. Standard convergence criteria as implemented in Gaussian 09 have always been met. Enthalpic and entropic contributions were calculated by means of statistical thermodynamics as implemented in the Gaussian 09 set of programs. Compliance matrices/submatrices were calculated using the Compliance 3.0.2. software by *Grunenberg et al.* from the previously geometry optimised structures [5,6].

a. [(d(CH$_2$P(*i*Pr)$_2$)abB)M(PMe$_3$)$_n$] (2a–c (*n* = 2), 3a–c (*n* = 1)) and [(d(CH$_2$P(*i*Pr)$_2$)abB)M(N$_2$)(PMe$_3$)] (4a–c)

Table S9. Energies of the calculated stationary points for [(d(CH$_2$P(*i*Pr)$_2$)abB)M(PMe$_3$)$_n$] (**2a–c** (*n* = 2), **3a–c** (*n* = 1)) and [(d(CH$_2$P(*i*Pr)$_2$)abB)M(N$_2$)(PMe$_3$)] (**4a–c**).

**Compound**			E_{el} / hartree (kJ mol^{-1})	$E_{el} + E_0$ / hartree (kJ mol^{-1})	G_{298} / hartree (kJ mol^{-1})
**M = Co**	***n* = 2**	**2a**	–3906.68189689 (–1.03·10^7)	–3905.917613 (–1.03·10^7)	–3905.988962 (–1.03·10^7)
	***n* = 1**	**3a**	–3445.47227338 (–9.05·10^6)	–3444.825994 (–9.04·10^6)	–3444.895673 (–9.04·10^6)
		**4a**	–3555.07644836 (–9.33·10^6)	–3554.420547 (–9.33·10^6)	–3554.490308 (–9.33·10^6)
**M = Rh**	***n* = 2**	**2b**	–2634.46055851 (–6.92·10^6)	–2633.698896 (–6.91·10^6)	–2633.773826 (–6.91·10^6)
	***n* = 1**	**3b**	–2173.26687319 (–5.71·10^6)	–2172.620763 (–5.70·10^6)	–2172.690043 (–5.70·10^6)
		**4b**	–2282.84011246 (–5.99·10^6)	–2282.185314 (–5.99·10^6)	–2282.256626 (–5.99·10^6)
**M = Ir**	***n* = 2**	**2c**	–2628.28112715 (–6.90·10^6)	–2627.517936 (–6.90·10^6)	–2627.591817 (–6.90·10^6)
	***n* = 1**	**3c**	–2167.08595054 (–5.69·10^6)	–2166.439282 (–5.69·10^6)	–2166.508382 (–5.69·10^6)
		**4c**	–2276.65933913 (–5.98·10^6)	–2276.003737 (–5.98·10^6)	–2276.074926 (–5.98·10^6)
		**N$_2$**	–109.57319636 (–2.88·10^5)	–109.567672 (–2.88·10^5)	–109.586104 (–2.88·10^5)
		**PMe$_3$**	–461.18445590 (–1.21·10^6)	–461.072446 (–1.21·10^6)	–461.101796 (–1.21·10^6)

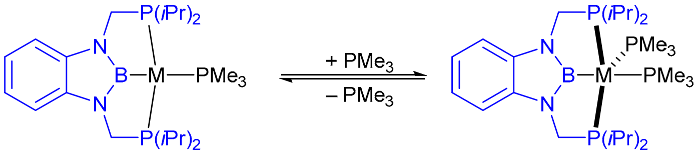

3a,b,c **2a,b,c**

	M = Co	M = Rh	M = Ir
$\Delta(E_{el} + E_0)$ /kJ mol^{-1}	–50.3	–14.9	–16.3
ΔG_{298} /kJ mol^{-1}	22.3	47.3	48.2

Table S10. Relative energies of the addition of PMe$_3$ to [(d(CH$_2$P(*i*Pr)$_2$)abB)M(PMe$_3$)] (**3a–c**) (M = Co, Rh, Ir).

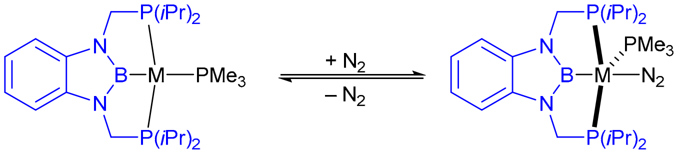

3a,b,c **4a,b,c**

	M = Co	M = Rh	M = Ir
$\Delta(E_{el} + E_0)$ /kJ mol^{-1}	–70.6	8.2	8.4
ΔG_{298} /kJ mol^{-1}	–22.4	51.3	51.4

Table S11. Relative energies of the addition of N_2 to [(d($CH_2P(iPr)_2$)abB)M(PMe_3)] (**3a–c**) (M = Co, Rh, Ir).

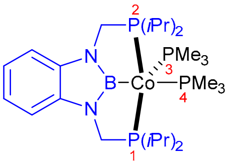

2a

Table S12. Compliance Matrix of [(d($CH_2P(iPr)_2$)abB)Co(PMe_3)$_2$] (**2a**) (Submatrix containing selected relevant coordinates in cm N^{-1})

**STRE**	Co–P1	Co–P2	Co–P3	Co–P4	Co–B
Co–P1	0.860				
Co–P2	–0.140	0.772			
Co–P3	–0.081	–0.066	0.736		
Co–P4	–0.011	–0.008	–0.007	0.733	
Co–B	0.001	0.011	0.010	–0.089	0.431

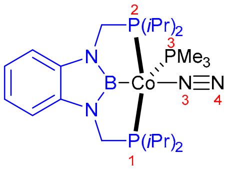

4a

Table S13. Compliance Matrix of [(d($CH_2P(iPr)_2$)abB)Co(N_2)(PMe_3)] (**4a**) (Submatrix containing selected relevant coordinates in cm N^{-1})

**STRE**	Co–P1	Co–P2	Co–P3	Co–N3	Co–B	N3–N4
Co–P1	0.743					
Co–P2	–0.142	0.789				
Co–P3	–0.046	–0.065	0.771			
Co–N3	–0.007	–0.003	–0.018	0.429		
Co–B	0.005	0.003	0.009	–0.063	0.439	
N3–N4	0.002	0.002	0.003	–0.019	0.004	0.052

4. References

1. Frisch, M.J.; Trucks, G.W.; Schlegel, H.B.; Scuseria, G.E.; Robb, M.A.; Cheeseman, J.R.; Scalmani, G.; Barone, V.; Mennucci, B.; Petersson, G.A.; et al. *Gaussian 09, Revision D.01;* Gaussian, Inc.: Wallingford, CT, USA, 2013.
2. Tao, J.; Perdew, J. P.; Staroverov, V. N.; Scuseria, G. E. Climbing the Density Functional Ladder: Nonempirical Meta-Generalized Gradient Approximation Designed for Molecules and Solids. *Phys. Rev. Lett.* **2003**, *91*, 146401-1–146401-4. https://doi.org/10.1103/PhysRevLett.91.146401.
3. Staroverov, V. N.; Scuseria, G.E.; Tao, J.; Perdew, J.P. Comparative assessment of a new nonempirical density functional: Molecules and hydrogen-bonded complexes. *J. Chem. Phys.* **2003**, *119*, 12129–12137. https://doi.org/10.1063/1.1626543.
4. Weigend, F.; Ahlrichs, R. Balanced basis sets of split valence, triple zeta valence and quadruple zeta valence quality for H to Rn: Design and assessment of accuracy. *Phys. Chem. Chem. Phys.* **2005**, *7*, 3297–3305. http://dx.doi.org/10.1039/b508541a.
5. Brandhorst, K.; Grunenberg, J. Efficient computation of compliance matrices in redundant internal coordinates from Cartesian Hessians for nonstationary points. *J. Chem. Phys.* **2010**, *132*, 184101-1–184101-7. http://dx.doi.org/10.1063/1.3413528.
6. Brandhorst, K.; Grunenberg, J. How strong is it? The interpretation of force and compliance constants as bond strength descriptors. *Chem. Soc. Rev.* **2008**, *37*, 1558–1567. https://doi.org/10.1039/B717781J.
